# Fe_3_O_4_@SiO_2_ core/shell functionalized by gallic acid: a novel, robust, and water-compatible heterogeneous magnetic nanocatalyst for environmentally friendly synthesis of acridine-1,8-diones†

**DOI:** 10.1039/d4ra00629a

**Published:** 2024-04-03

**Authors:** Zahra Firoozi, Dariush Khalili, Ali Reza Sardarian

**Affiliations:** a Department of Chemistry, College of Sciences, Shiraz University Shiraz 71467-13565 Iran khalili@shirazu.ac.ir

## Abstract

In this study, we conveniently prepared a novel robust heterogeneous magnetic nanocatalyst using a Fe_3_O_4_@SiO_2_ core/shell stabilized by gallic acid. The catalyst was completely characterized by various physicochemical techniques, including infrared spectroscopy (FT-IR), X-ray diffraction (XRD), dynamic light scattering (DLS), transmission electron microscopy (TEM), field emission scanning electron microscopy (FE-SEM), thermogravimetric analysis (TGA), potentiometric titration, energy dispersive X-ray microanalysis (EDX), vibrating sample magnetometer (VSM), zeta potential analysis, and BET. The potential ability of the newly developed sulfonated nanocatalyst was then exploited in the multicomponent synthesis of acridine-1,8-dione derivatives by considering the green chemistry matrix and under mild conditions. Various aldehydes and amines were smoothly reacted with dimedone, affording the desired products in good to excellent yields. The introduction of sulfonic groups using gallic acid allowed the development of a water-compatible and highly recyclable catalytic system for reactions in an aqueous environment. The prepared catalyst can be readily magnetically separated and reused eight times without significant loss of activity. High synthetic efficiency, using a recyclable and eco-sustainable catalyst under mild conditions, and easy product isolation are salient features of this catalytic system, which makes this protocol compatible with the demands of green chemistry.

## Abstract 2

An efficient route for the construction of acridine-1,8-diones using Fe_3_O_4_@SiO_2_ core/shell functionalized by sulfonated gallic acid as a robust heterogeneous catalyst.

## Graphical abstract

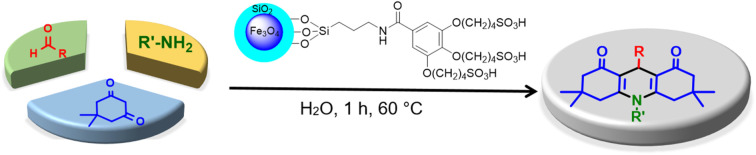

## Introduction

Nowadays, nanoscience has become one of the most essential areas in science and technology that has played an integral role in efficient organic transformations over the past few decades. In this regard, nanomaterials have gained great attention, especially in chemistry and medical research.^1^ Among the nanosupports, magnetic nanoparticles (MNPs) have been widely used in memory storage devices,^2^ biological separation,^3^ biomedical applications,^4^ and catalytic processes,^5^ mainly owing to their versatile physical surface, and inherent adsorptive properties, and active sites. On the basis of its great biocompatibility, low toxicity, ease of synthesis, and recycling, magnetite (Fe_3_O_4_), the most popular magnetic support, has drawn a lot of interest.^6^ The easy separation of iron oxide NPs through magnetic decantation makes it a more sustainable catalyst.^7^ Despite the monodispersion of particle size, hydrophobicity has limited the use of magnetite for biological applications.^8^ To remove this obstacle, it is necessary to transfer the hydrophobic NPs to aqueous media by surface modification using polymer or silica coating methods.^9^ Among these techniques, silica coating with the ability to be conjugated with various functional groups and nontoxicity has shown promise.^10^ Recently, in conjunction with silica-supported materials, gallic acid (3,4,5-trihydroxybenzoic acid, GA), a natural plant triphenol, has been used as a valuable motif for functionalizing the coated magnetic nanoparticles.^11^ In this context, useful templates can be achieved for the generation of more functionalized hybrid materials by immobilizing gallic acid on aminopropyl-modified silica,^12^ which could potentially be used to enable catalytic transformations. The presence of reactive hydroxyl groups in the structure of gallic acid appears to be essential for the functionalization of gallic acid with sultone to produce acidic sulfonic groups. The produced heterogeneous acid catalyst can then be used as a sustainable catalyst for the production of valuable chemicals.^13^

Multicomponent reaction (MCR) chemistry provides a flexible synthetic toolbox to access an endless list of substituted heterocyclic systems in a convergent way. MCRs are superior to classical methods because they are more environmentally friendly, have a lower atom/step economy, and avoid tedious purification procedures.^14^ Acridines, and especially acridine-1,8-diones, are a significant class of fused heterocycles on account of their ubiquitous presence and wide potential applications in biological and synthetic molecules.^15^ Study reports on acridine-1,8-diones show that they have a wide range of medicinal activities, including antimicrobial,^16^ antimalarial,^17^ antitumor,^18^ anticancer,^19^ antibacterial,^20^ and fungicidal,^21^ activities. Besides, they are of interest because of their unique photochemical and electrochemical behavior.^22^ Different catalytic approaches to accessing acridines are known and well documented in the literature, including the use of acid catalysts,^15*c*,23^ magnetic nanocatalysts,^24^ metals,^25^ and ionic liquid catalysts.^26^ Nevertheless, in order to achieve these transformations, it is necessary to utilize harsh reaction conditions such as high temperatures, strong acid catalysts, tedious work-up procedures, and longer reaction times. As environmental concerns grow, the demand for sustainable synthetic methods has become crucial for organic transformations on any scale and has remained an important subject. In this context, we disclose the divergent route for the construction of acridine-1,8-diones using Fe_3_O_4_@SiO_2_ core/shell functionalized by sulfonated gallic acid as a robust heterogeneous catalyst. Incorporating sulfonic functional groups through the use of gallic acid facilitates the formation of a water-compatible and exceptionally recyclable catalytic system for MCR reactions within an aqueous environment. This newly developed catalytic system demonstrates wide substrate scope under mild conditions with the addition of a low amount of catalyst. The utilization of an environmentally benign solvent, such as water, in the present catalytic system is also a prominent area of interest in the field of sustainability. The current established catalytic system not only opens an avenue to access 1,4-dihydropyridine (1,4-DHP) fragments^27^ under mild conditions but also demonstrates several unique characteristics pertinent to sustainable organic synthesis, including a recyclable catalyst and facile product separation, solar cells, light-emitting sensors, and ion detection.

## Experimental

### Synthesis of Fe_3_O_4_ MNPS

First, a solution containing FeCl_2_·4H_2_O (3.0 mmol, 0.50 g) and FeCl_3_·6H_2_O (6.0 mmol, 0.8 g) in 150.0 mL of deionized water was prepared, and 1.0 g of polyvinyl alcohol (PVA 15000) was added as a surfactant. The resulting mixture was vigorously stirred for 30 minutes at 80 °C. To the resulting solution, hexamethylenetetramine (HMTA) (1 M) was added dropwise to adjust the pH to 10 and obtain a black suspension. Next, the black mixture was stirred for a further 2 hours at 60 °C. Finally, the resulting black powder (Fe_3_O_4_ MNPs) (1) was separated by an external magnet, washed three times with deionized water and ethanol, and dried at 80 °C for 10 h.^28^

### Synthesis of Fe_3_O_4_@SiO_2_ core/shell MNPs

0.5 g of Fe_3_O_4_ MNPs was dispersed in 5.0 mL of deionized water. Next, a mixture of 50.0 mL of ethanol and 0.5 mL of NaOH (10% w/w) was added to the magnetic suspension. Then, 0.2 mL of tetraethyl orthosilicate (TEOS) was added dropwise to the mixture and the mixture, was stirred for 30 minutes at room temperature. The obtained Fe_3_O_4_@SiO_2_ MNPs (2) were separated by an external magnet, washed three times with deionized water and ethanol, and dried at 80 °C for 10 hours.^28^

### Synthesis of Fe_3_O_4_@SiO_2_-NH_2_ MNPs

In this step, 0.5 g of Fe_3_O_4_@SiO_2_ MNPs was added to a solution of 3-(triethoxysilyl) propylamine (APTES) (1.0 mmol, 0.23 mL) in 5.0 mL of ethanol. The resulting mixture was refluxed for 12 hours. After that, the mixture was cooled to room temperature, and the resulting Fe_3_O_4_@SiO_2_-NH_2_ MNPs (3) were separated by an external magnetic field, washed three times with deionized water and subsequently with ethanol, and dried at 80 °C for 10 hours.^29^

### Synthesis of Fe_3_O_4_@SiO_2_-NH-GA-[(CH_2_)_4_-SO_3_H]_3_ MNPs

Gallic acid (3.0 mmol, 0.5 g), *N*-hydroxysuccinimide (NHS) (2.6 mmol, 0.305 g), and *N*,*N*′-dicyclohexylcarbodiimide (DCC) (2.8 mmol, 0.57 g) were mixed in 100.0 mL of borate buffer (pH = 11), and the resulting mixture was stirred for 30 minutes at 40 °C. To the resulting mixture, 0.5 g of Fe_3_O_4_@SiO_2_-NH_2_ MNPs (3) was added at 40 °C under an N_2_ atmosphere for 6 h. The obtained suspension of Fe_3_O_4_@SiO_2_-NH-GA MNPs (4) was then treated with 1,4-butane sultone and H_2_SO_4_ (0.1 M) to afford a mixture containing Fe_3_O_4_@SiO_2_-NH-GA-[(CH_2_)_4_-SO_3_H]_3_ MNPs (5). Finally, the resulting mixture was stirred at 70 °C for 24 hours to complete the nanocatalyst formation. The nanohybrid material (5) was then easily separated magnetically, washed three times with deionized water and ethanol, and dried at 80 °C for 10 hours. The acid capacity and concentration of sulfonic groups in the sample were determined by titration with 0.01 M NaOH. The acid capacity of the obtained sulfonic silica material was found to be 18 mmol H^+^ g^−1^.

### General method for the preparation of acridine-1,8-dione

First, a 25 mL round-bottom flask was charged with a mixture of aldehyde (1.0 mmol), dimedone (2.0 mmol, 0.28 g), ammonium acetate or anilines (1.1 mmol), and 0.02 g of Fe_3_O_4_@SiO_2_-NH-GA-[(CH_2_)_4_-SO_3_H]_3_ MNPs and then stirred in water (2.0 mL) at 60 °C for the indicated time. After completion, the magnetic nanocatalyst was retrieved by magnetic separation. The solid was washed three times with ethanol, dried overnight in an oven at 70 °C, and reused for the next time. The residual reaction mixture was extracted with ethyl acetate (3 × 5 mL), and the combined organic layer was dried over anhydrous Na_2_SO_4_ and concentrated under reduced pressure. The resulting crude product was purified by column chromatography (silica gel 200–300 mesh, petroleum ether : EtOAc) to obtain corresponding acridine-1,8-diones.

## Result and discussion

**Scheme 1:**
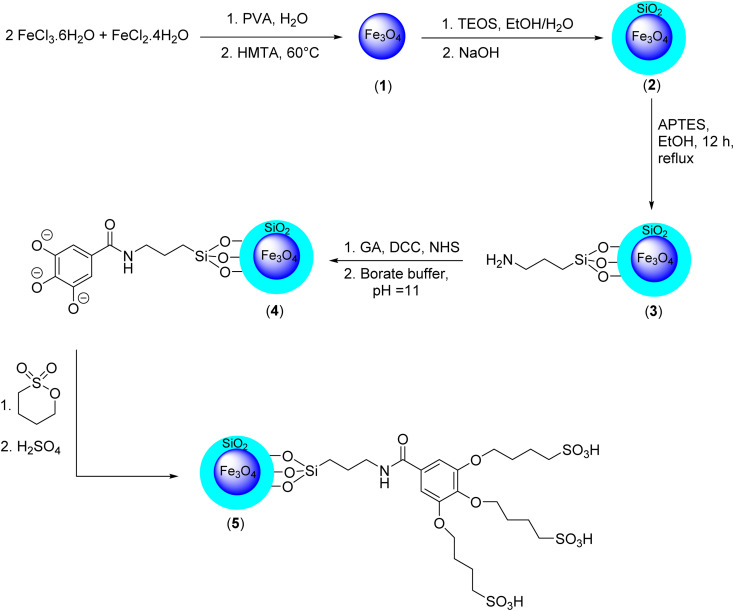
Concise procedure for the preparation of the Fe_3_O_4_@SiO_2_-NH-GA-[(CH_2_)_4_-SO_3_H]_3_ MNPs.

The current study has outlined a methodological approach consisting of five sequential steps for the synthesis of Fe_3_O_4_@SiO_2_-NH-GA-[(CH_2_)_4_-SO_3_H]_3_ nanocomposites (Scheme 1).

The study commenced with the application of a sol–gel coating technique to deposit a slender layer of silica onto pre-fabricated Fe_3_O_4_ MNPs.^30^ To generate the amine-functionalized mesostructured composite on the SiO_2_@Fe_3_O_4_ surface, 3-(triethoxysilyl)propylamine (APTES) was used as a functional precursor. Then, treatment of the obtained bifunctionalized magnetic core-mesoporous silica shell nanocomposite (3) with gallic acid, *N*-hydroxysuccinimide (NHS), and *N*,*N*′-dicyclohexylcarbodiimide in the presence of borate buffer, followed by the addition of 1,4-butane sultone, introduces –SO_3_H acidic functional groups to the structure of the Fe_3_O_4_@SiO_2_ core/shell functionalized by gallic acid (4) and produces the final catalyst (5). The structure of the prepared materials was characterized by various techniques, including Fourier transform infrared (FT-IR) spectroscopy, X-ray diffraction (XRD), field emission scanning electron microscopy (FESEM), energy-dispersive X-ray (EDX), transmission electron microscopy (TEM), VSM (vibrating-sample magnetometer), and thermogravimetric analysis (TGA).

### FT-IR analysis

**Fig. 1:**
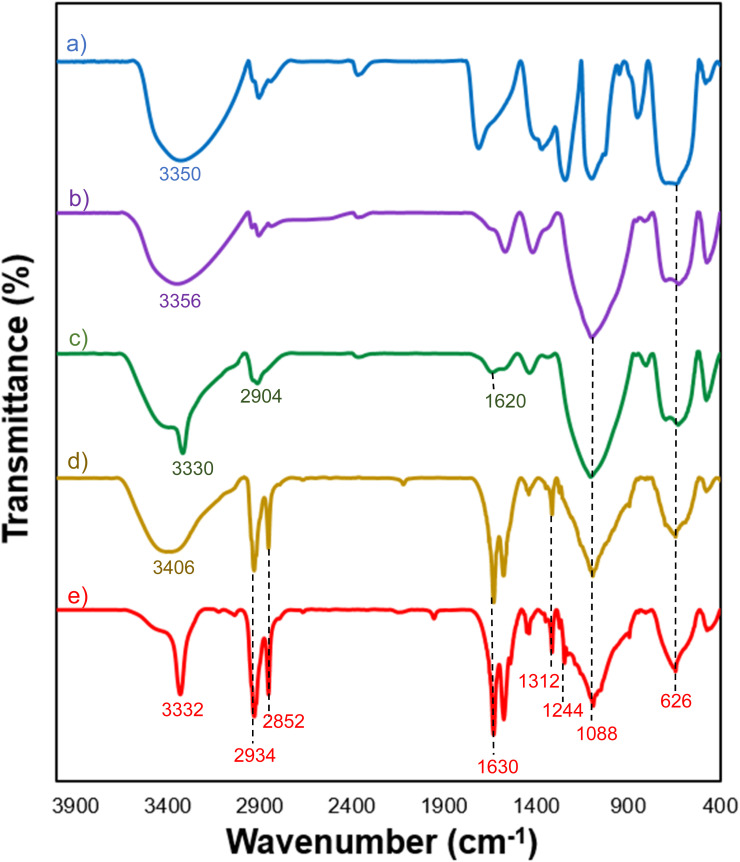
FT-IR spectra of: (a) Fe_3_O_4_, (b) Fe_3_O_4_@SiO_2_, (c) Fe_3_O_4_@SiO_2_-NH_2_, (d) Fe_3_O_4_@SiO_2_-NH-GA, and (e) Fe_3_O_4_@SiO_2_-NH-GA-[(CH_2_)_4_-SO_3_H]_3_ MNPs.

The FT-IR spectrum revealed that the Fe_3_O_4_ displayed two vibration bands at 626 cm^−1^ and 3350 cm^−1^, which are indicative of the typical Fe–O and O–H bonds.^31^ (Fig. 1a). The FT-IR spectrum of Fe_3_O_4_@SiO_2_ MNPs' exhibited a new peak at 1088 cm^−1^, which is attributed to the asymmetric stretching vibrations of the Si–O–Si bonds,^32^ indicating the successful coating of a thin silica shell on the Fe_3_O_4_ surface (Fig. 1b). For Fe_3_O_4_@SiO_2_-NH_2_ MNPs, additional peaks were observed in 1620, 2904, and 3330 cm^−1^ that correspond to the bending vibrations of the N–H bond, stretching vibrations of the aliphatic C–H bond, and NH-stretching (Fig. 1c) modes. The typical peak at 1620 cm^−1^ confirms the presence of terminal –NH_2_ on the surface of particles after decorating.^33^ The gallic acid-functionalized Fe_3_O_4_@SiO_2_-NH_2_ sample exhibited several additional absorption peaks (Fig. 1d). Three absorbance peaks around 1088, 2934, and 3406 cm^−1^ were indicative of the C–O, C–H, and O–H vibrations of the phenyl ring of gallic acid, while the other four additional absorbance bands around 1312, 1626, 1576, and 2852 cm^−1^ were attributed to the stretching vibrations of C–N and C=O, bending vibrations of the N–H bond, and symmetric stretching modes of the C–H bond in the alkane chain, revealing the formation of an amide bond. Finally, successful –SO_3_H functionalization of Fe_3_O_4_@SiO_2_-NH_2_-GA was confirmed by the presence of S=O stretching vibration at 1244 cm^−1^ (Fig. 1e).

### XRD analysis

**Fig. 2:**
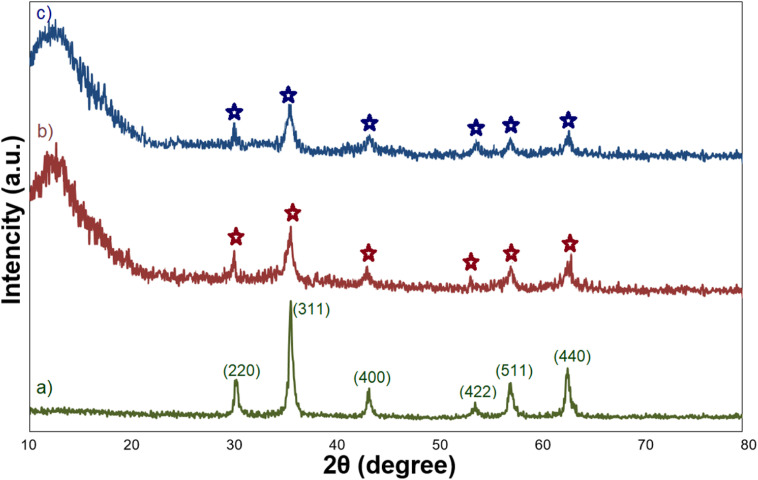
XRD patterns of (a) Fe_3_O_4_, (b) Fe_3_O_4_@SiO_2_, and (c) Fe_3_O_4_@SiO_2_-NH-GA-[(CH2)_4_-SO_3_H]_3_ MNPs.

Fig. 2 shows the XRD patterns of Fe_3_O_4_, Fe_3_O_4_@SiO_2_, and Fe_3_O_4_@SiO_2_-GA-[(CH_2_)_4_-SO_3_H]_3_ MNPs. The XRD pattern of Fe_3_O_4_ showed the characteristic peaks at 2*θ* = 30.0°, 35.6°, 43.6°, 53.9°, 57.2°, and 62.8°, which are attributed to the crystal planes [220], [311], [400], [422], [511], and [440], respectively. All the observed peaks can be exactly indexed to the diffractions of the Fe_3_O_4_ crystal in the cubic spinel structure (JCPDS Card no 19-629).^34^ These peaks are also preserved for Fe_3_O_4_@SiO_2_ MNPs and Fe_3_O_4_@SiO_2_-GA-[(CH_2_)_4_-SO_3_H]_3_ MNPs, but due to the coating process by the SiO_2_ shell and then the organic layer, the intensity of these peaks slightly decreased. These results also confirm that surface modification has no effect on the crystalline properties of magnetite. The presence of a broad diffraction peak at 2*θ* around 15–25° in the XRD patterns of Fe_3_O_4_@SiO_2_ and Fe_3_O_4_@SiO_2_-GA-[(CH_2_)_4_-SO_3_H]_3_ is attributed to the existence of amorphous SiO_2_. After coating with SiO_2_ and organic modification with gallic acid and sultone, no new peak was observed.

**Fig. 3:**
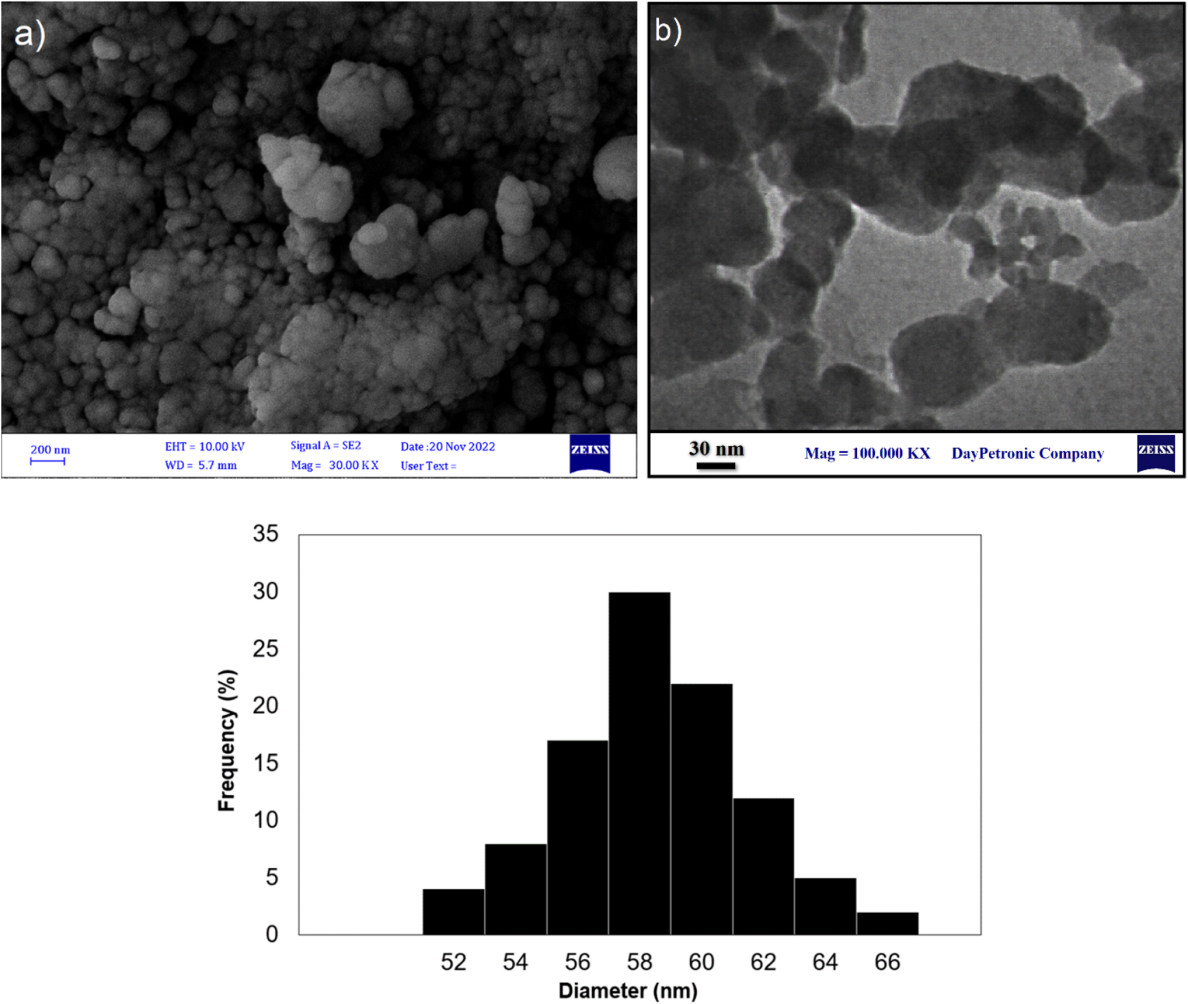
(a) FE-SEM image, (b) TEM image, and (c) the particle distributions of Fe_3_O_4_@SiO_2_-NH-GA-[(CH_2_)_4_-SO_3_H]_3_ MNPs.

The surface morphology and particle distributions of the corresponding magnetic core–shell nanocomposites Fe_3_O_4_@SiO_2_-NH-GA-[(CH_2_)_4_-SO_3_H]_3_ were examined by field emission scanning electron microscopy (FESEM) and transmission electron microscopy (TEM). The FESEM and TEM image analyses (Fig. 3a and b) suggested that the final magnetic core–shell nanoparticles are in the 56–60 nm range (confirmed by DLS analysis) and have almost spherical shapes with a transparent core–shell structure. DLS analysis of the final nanocatalyst (Fig. 3c) obviously showed an average of 58 nm in size distribution.

**Fig. 4:**
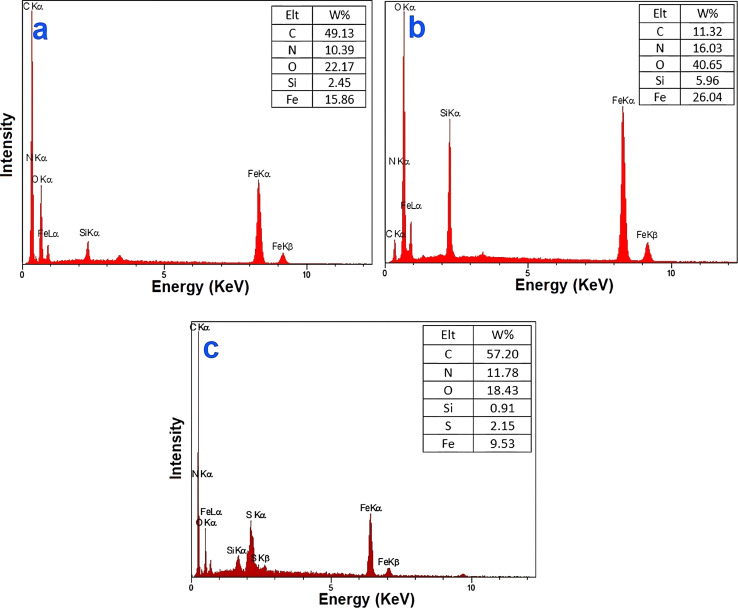
EDX spectrum of (a) Fe_3_O_4_@SiO_2_-NH_2_, (b) Fe_3_O_4_@SiO_2_-NH-GA, (c) Fe_3_O_4_@SiO_2_-NH-GA-[(CH_2_)_4_-SO_3_H]_3_ MNPs.

The EDX results for Fe_3_O_4_@SiO_2_-NH_2_, Fe_3_O_4_@SiO_2_-NH-GA, and Fe_3_O_4_@SiO_2_-NH-GA-[(CH_2_)_4_-SO_3_H]_3_ and Fe_3_O_4_@SiO_2_-NH-GA-[(CH_2_)_4_-SO_3_H]_3_ are shown in Fig. 4, which clearly demonstrates successful loading of organic layers and the presence of Fe, C, Si, N, O, and S elements in the Fe_3_O_4_@SiO_2_-NH-GA-[(CH_2_)_4_-SO_3_H]_3_ nanocatalyst.

**Fig. 5:**
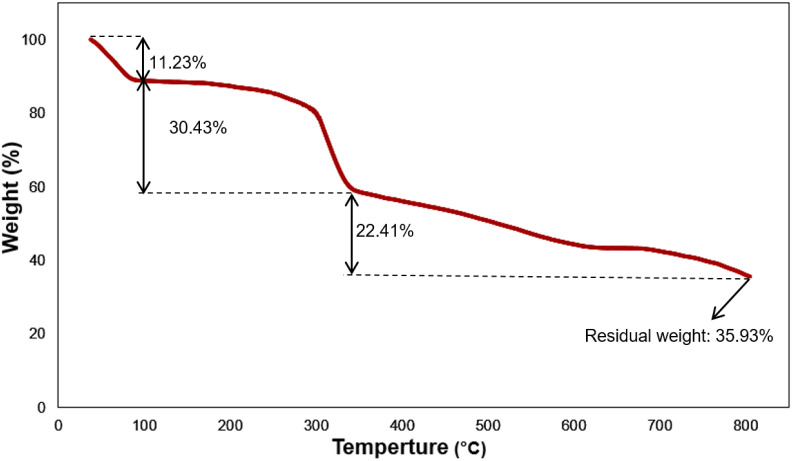
Graph of thermal gravimetric analysis of Fe_3_O_4_@SiO_2_-NH-GA-[(CH_2_)_4_-SO_3_H]_3_ MNPs.

The thermal behavior of Fe_3_O_4_@SiO_2_-NH-GA-[(CH_2_)_4_-SO_3_H]_3_ was also investigated using TG analysis at a heating rate of 10 °C min^−1^ (from 37 to 805 °C) under an N_2_ atmosphere, and the related curve is shown in Fig. 5. The initial weight loss of the final hybrid (5) up to 100 °C is 11.23%, which is due to the removal of physically adsorbed water and surface hydroxyl groups. 30.43% mass loss at 100–340 °C is attributed to the decomposition of SO_3_H groups and gallic acid grafted to the amine functional groups.^35^ As shown in Fig. 5, for Fe_3_O_4_@SiO_2_-NH-GA-[(CH_2_)_4_-SO_3_H]_3_ nanocatalyst, the gradual mass loss above 340 °C (22.41% mass loss) might result from the further removal of organic functional groups and decomposition of SO_3_H groups grafted to the silica surface.^35^ The residual weight of the Fe_3_O_4_@SiO_2_-NH-GA-[(CH_2_)_4_-SO_3_H]_3_ nanocatalyst was 35.93%.

### Vibrating sample magnetometry (VSM) analysis of Fe_3_O_4_@SiO_2_-NH-GA-[(CH_2_)_4_-SO_3_H]_3_ MNPs

**Fig. 6:**
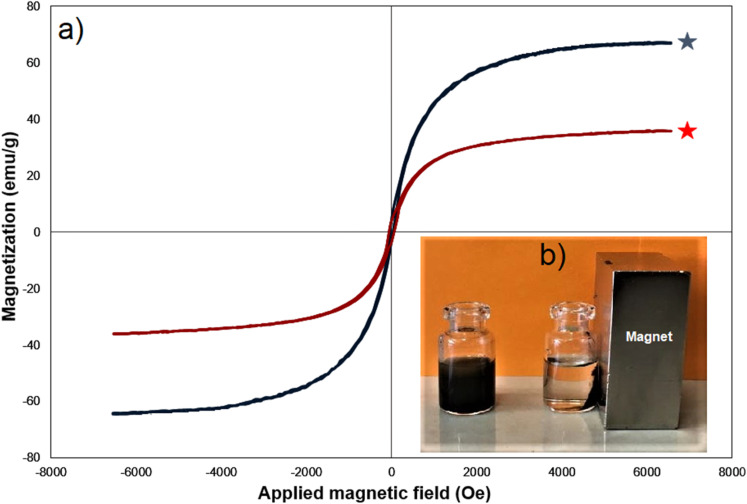
(a) The VSM curves for Fe_3_O_4_ nanoparticles (⋯) and Fe_3_O_4_@SiO_2_-NH-GA-[(CH_2_)_4_-SO_3_H]_3_ (⋯) (b) the ability of the catalyst to be effectively recovered at the end of the reactions by an external magnetic field.

Vibrating sample magnetometry (VSM) is a common technique used to measure the magnetic properties of materials. In this case, Fe_3_O_4_ and Fe_3_O_4_@SiO_2_-NH-GA-[(CH_2_)_4_-SO_3_H]_3_ MNPs (magnetic nanoparticles) were analyzed at room temperature using VSM to determine their magnetic behavior (Fig. 6). As illustrated in Fig. 6a, the two nanomaterials showed superparamagnetic characteristics without any hysteresis loop. The saturation magnetization of Fe_3_O_4_ and Fe_3_O_4_@SiO_2_-NH-GA-[(CH_2_)_4_-SO_3_H]_3_ MNPs were found to be 70.1 and 38.9 emu g^−1^, respectively (Fig. 6a). As expected, after functionalization with silica and subsequent sulfonated gallic acid, the *M*_s_ value of the final catalyst was reduced to 38.9 emu g^−1^. This reduction can be attributed to the non-magnetic nature of the SiO_2_-NH-GA-[(CH_2_)_4_-SO_3_H]_3_ coating, which acts as a barrier between the magnetic core and the surrounding environment.^36^

**Fig. 7:**
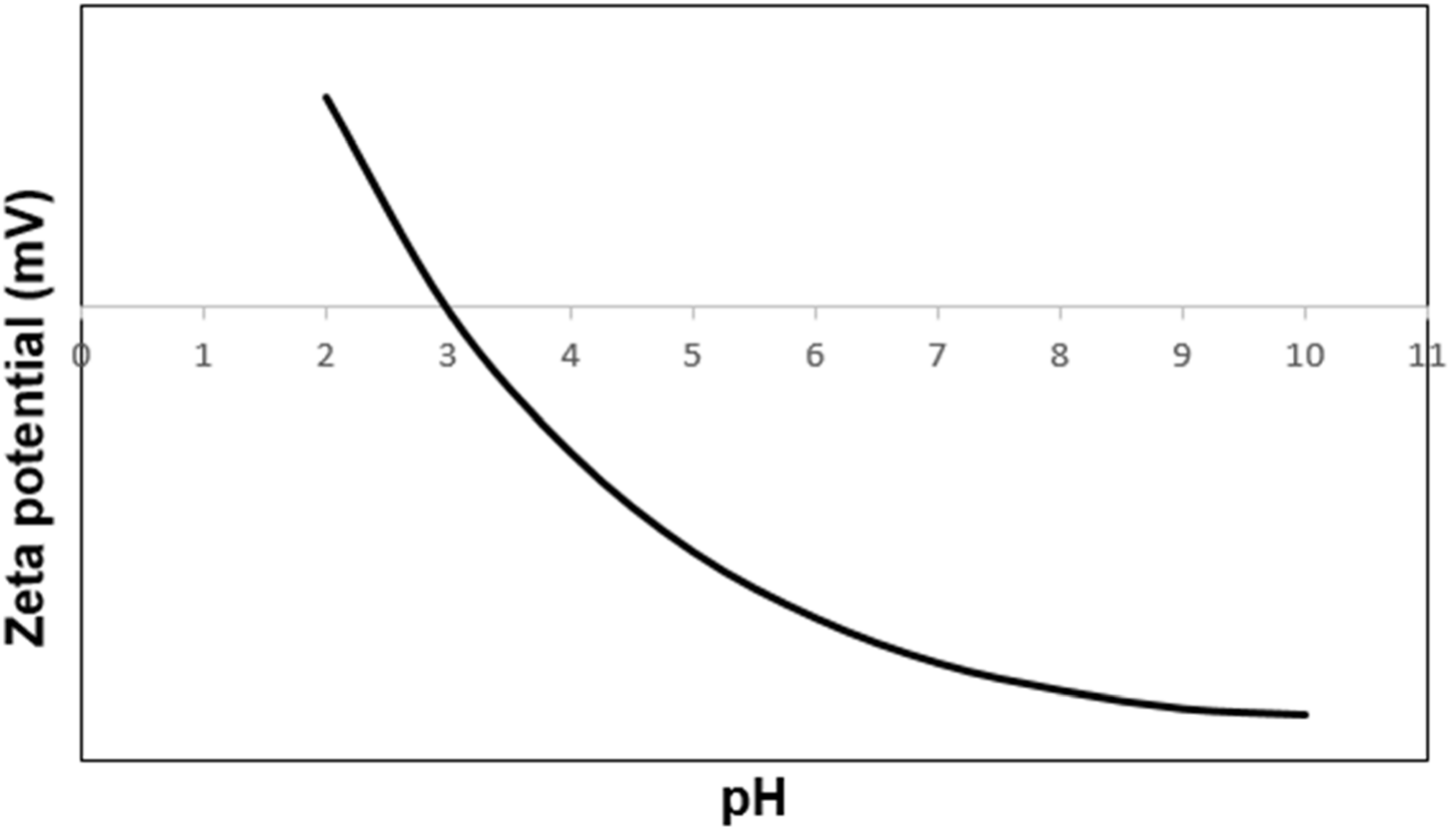
Zeta potential values *vs.* pH for Fe_3_O_4_@SiO_2_-NH-GA-[(CH2)_4_-SO_3_H]_3_ MNPs.

The changes in zeta potential *versus* pH for the catalyst containing –SO_3_H functional groups can also provide valuable information about the ionization of these groups and the resulting changes in the surface charge of the nanoparticles. As can be seen from Fig. 7, the isoelectric point of the Fe_3_O_4_@SiO_2_-NH-GA-[(CH_2_)_4_-SO_3_H]_3_ is at pH 3.1. The negatively charged sulfonic acid functional groups at high pH values result in a high negative zeta potential. As the pH is decreased, the –SO_3_H groups become progressively less ionized and less negatively charged. This sets the stage for triggering a decrease in the negative surface charge of the nanoparticles and a decrease in the magnitude of the zeta potential.

**Fig. 8:**
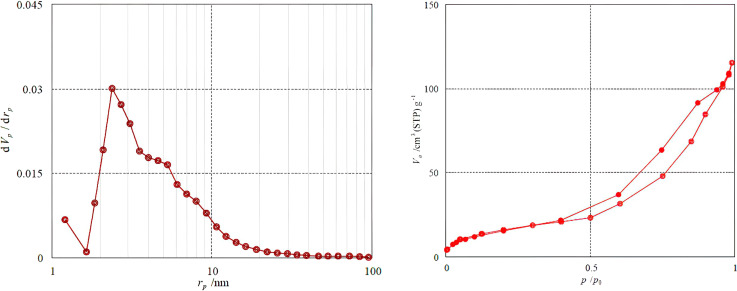
(a) Pore size distributions and (b) N_2_ adsorption–desorption isotherms of Fe_3_O_4_@SiO_2_-NH-GA-[(CH_2_)_4_-SO_3_H]_3_ NPs.

The surface and the porous property of the Fe_3_O_4_@SiO_2_-NH-GA-[(CH_2_)_4_-SO_3_H]_3_ NPs were investigated by the BET method *via* nitrogen adsorption and desorption measurement (Fig. 8).

The BET surface area of the Fe_3_O_4_@SiO_2_-NH-GA-[(CH_2_)_4_-SO_3_H]_3_ NPs was found to be 6.0184 m^2^ g^−1^. The total pore volume and the mean pore diameter of the catalyst were 0.1782 cm^3^ g^−1^ and 11.842 nm, respectively. Fig. 4a demonstrates that the pore size distribution is mainly concentrated between 2–10 nm. As can be seen from Fig. 4b, the N_2_ adsorption–desorption isotherms of the final catalyst are type IV, as classified by IUPAC, and have an H_3_-type hysteresis loop in the latter half part (*P*/*P*_0_ is 0.5–1.0), which is typical of mesoporous materials.^37^

After the characterization of the nanocomposite, the catalytic activity of Fe_3_O_4_@SiO_2_-NH-GA-[(CH_2_)_4_-SO_3_H]_3_ nanocatalyst was examined in the synthesis of acridine-1,8-diones by the reaction of benzaldehyde, dimedone, and ammonium acetate as a model reaction (Table 1). The study commenced using Fe_3_O_4_@SiO_2_-NH-GA-[(CH_2_)_4_-SO_3_H]_3_ nanocatalyst in toluene at 110 °C for 6 h, which led to the formation of the desired product, acridine-1,8-dione (4a), in 65% yield (Table 1, entry 1).

**Table 1:** Optimization of conditions and reaction parameters for the synthesis of acridine-1,8-dione 4a^a^

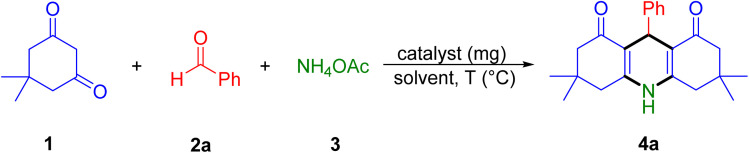					
Entry	Catalyst (mg)	Solvent	*T* (°C)	Time (h)	Yield^b^ (%)
1	Fe_3_O_4_@SiO_2_-NH-GA-[(CH_2_)_4_-SO_3_H]_3_ (5)	Toluene	Reflux	6	65
2	Fe_3_O_4_@SiO_2_-NH-GA-[(CH_2_)_4_-SO_3_H]_3_ (5)	THF	Reflux	6	47
3	Fe_3_O_4_@SiO_2_-NH-GA-[(CH_2_)_4_-SO_3_H]_3_ (5)	CHCl_3_	Reflux	6	68
4	Fe_3_O_4_@SiO_2_-NH-GA-[(CH_2_)_4_-SO_3_H]_3_ (5)	EtOH	Reflux	3	76
5	Fe_3_O_4_@SiO_2_-NH-GA-[(CH_2_)_4_-SO_3_H]_3_ (5)	H_2_O	Reflux	1	92
6	Fe_3_O_4_@SiO_2_-NH-GA-[(CH_2_)_4_-SO_3_H]_3_ (5)	—	80	6	54
7^c^	Fe_3_O_4_@SiO_2_-NH-GA-[(CH_2_)_4_-SO_3_H]_3_ (5)	H_2_O	60	1	90
8	Fe_3_O_4_@SiO_2_-NH-GA-[(CH_2_)_4_-SO_3_H]_3_ (5)	H_2_O	r.t	6	66
9	Fe_3_O_4_@SiO_2_-NH-GA-[(CH_2_)_4_-SO_3_H]_3_ (8)	H_2_O	60	1	89
10	Fe_3_O_4_@SiO_2_-NH-GA-[(CH_2_)_4_-SO_3_H]_3_ (3)	H_2_O	60	1	81
11	—	H_2_O	60	12	<5
12	Fe_3_O_4_	H_2_O	60	6	49
13	Fe_3_O_4_@SiO_2_	H_2_O	60	6	58
14	Fe_3_O_4_@SiO_2_-NH_2_	H_2_O	60	3	65
15	Fe_3_O_4_@SiO_2_-NH-GA	H_2_O	60	3	65

a Experimental conditions: benzaldehyde (1.0 mmol), dimedone (2.0 mmol), ammonium acetate (1.1 mmol), catalyst (type indicated), and solvent (2.0 mL).

b Yield of a pure isolated product.

c The bold value signifies the best reaction conditions.

Then the effect of different solvents on the reaction time and yield was investigated. The results demonstrated the inadequate yield and prolonged reaction time associated with the use of THF, CHCl_3_, and EtOH (entries 2–4). The formation of 4a was eventually found to be facile only in refluxing H_2_O with excellent yield in a considerably shorter time (1 h), and therefore, water was designated as the preferred solvent for the reaction (entry 5). The reaction in solvent-free conditions afforded acridine-1,8-dione (4a) only in poor yield (54%, entry 6). Interestingly, decreasing the reaction temperature to 60 °C afforded nearly the same yield for 4a in water (90%, entry 7). Nonetheless, the further decrease in the reaction temperature did not disclose any advantageous outcomes (entry 8). No remarkable additional changes were observed upon increasing the amounts of the catalyst to 8 mg (entry 9). A slightly lower yield was obtained when the catalyst loading was decreased to 3 mg (entry 10). When the model reaction was carried out in the absence of any catalyst, the desired product 4a was not obtained (entry 11). Furthermore, a comparison of the reactivity of Fe_3_O_4_, Fe_3_O_4_@SiO_2_, Fe_3_O_4_@SiO_2_-NH_2_, and Fe_3_O_4_@SiO_2_-NH-GA with that of Fe_3_O_4_@SiO_2_-NH-GA-[(CH_2_)_4_-SO_3_H]_3_ revealed that the formers exhibit notably inferior performance for the model reaction (entries 12–15).

With the optimized reaction conditions established, we turned our focus towards exploring the potential of Fe_3_O_4_@SiO_2_-NH-GA-[(CH_2_)_4_-SO_3_H]_3_ for mediating the three-component synthesis of acridine-1,8-diones, 4. First, a series of aldehydes were reacted under the optimized conditions. The results show that aldehydes with both electron-withdrawing and electron-donating substituents are suitable substrates for this transformation (spectral data are given in Fig. S1–S66†). It is worth mentioning that electron-deficient aldehydes such as nitrobenzaldehydes showed better reactivity and provided the corresponding acridine-1,8-diones 4b–4d in good to excellent yields (Table 2, entries 2–4). Halo-substituted aryl aldehydes survived well, leading to halo-substituted acridines (entries 5–8).

**Table 2:** Fe_3_O_4_@SiO_2_-NH-GA-[(CH_2_)_4_-SO_3_H]_3_ catalyzed the synthesis of acridine-1,8-diones^a^

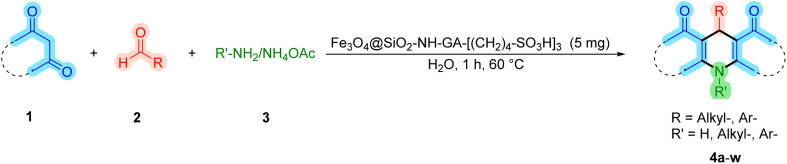		
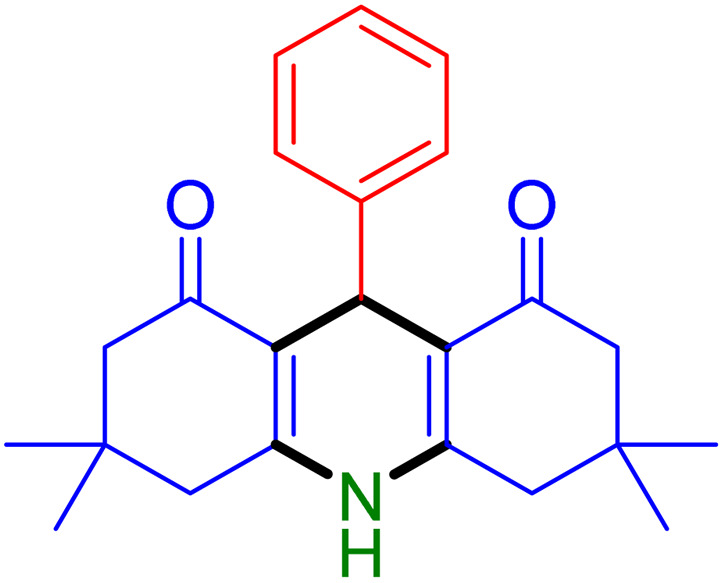	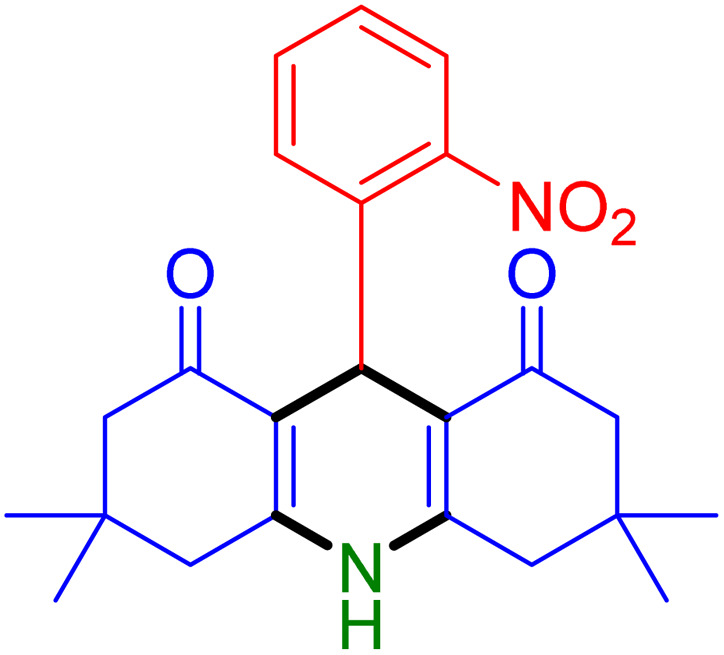	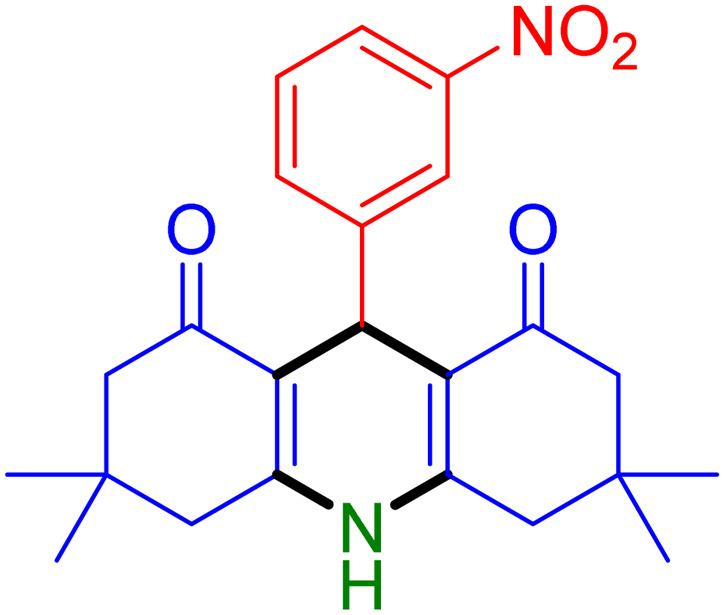
Entry 1: 4a, 90%^b^	Entry 2: 4b, 88%	Entry 3: 4c, 94%
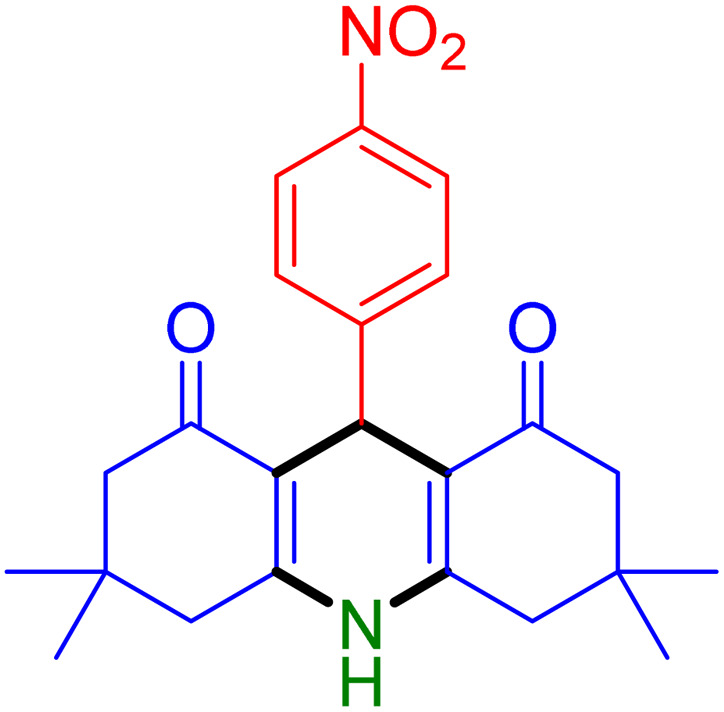	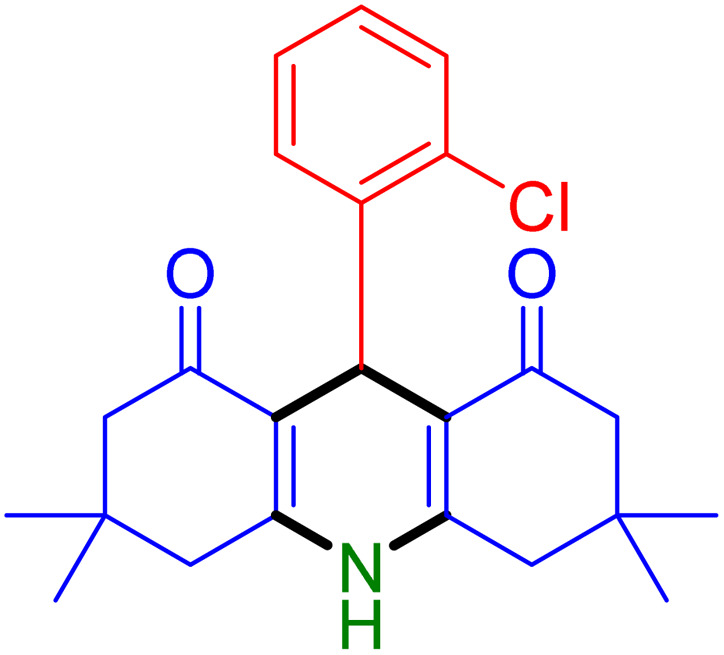	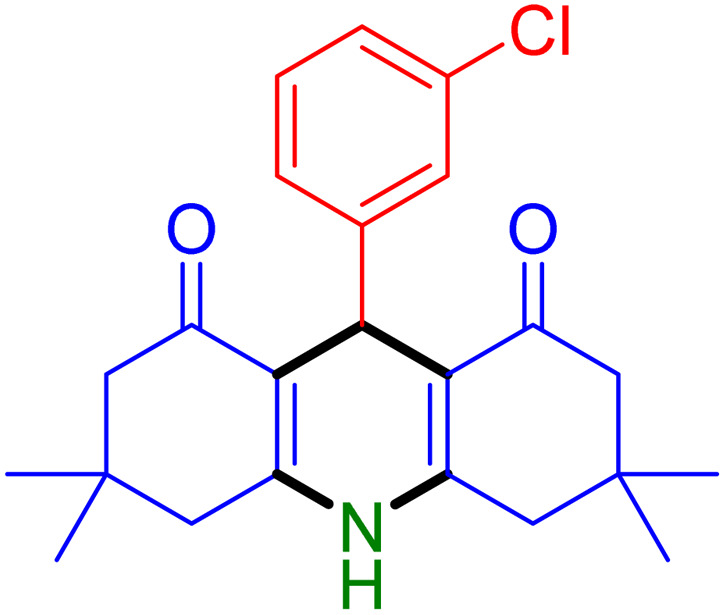
Entry 4: 4d, 96%	Entry 5: 4e, 85%	Entry 6: 4f, 87%
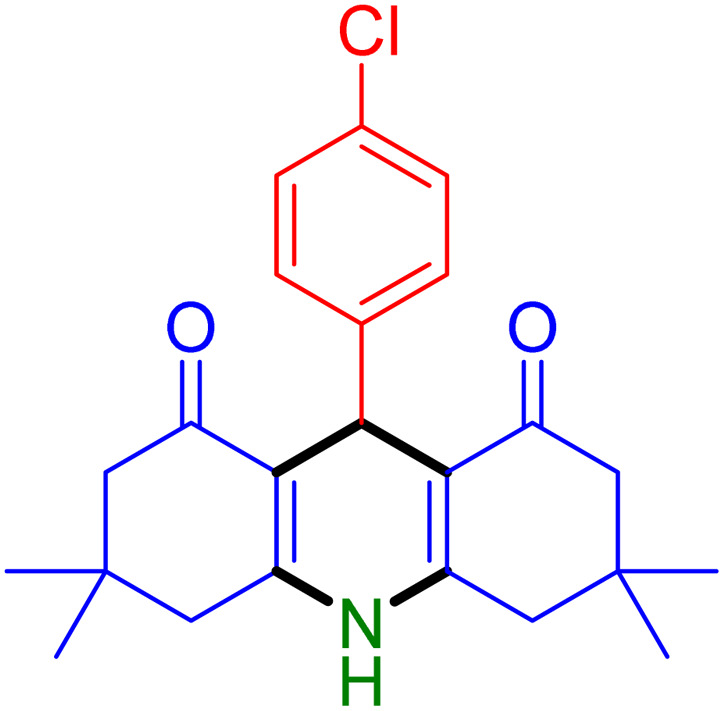	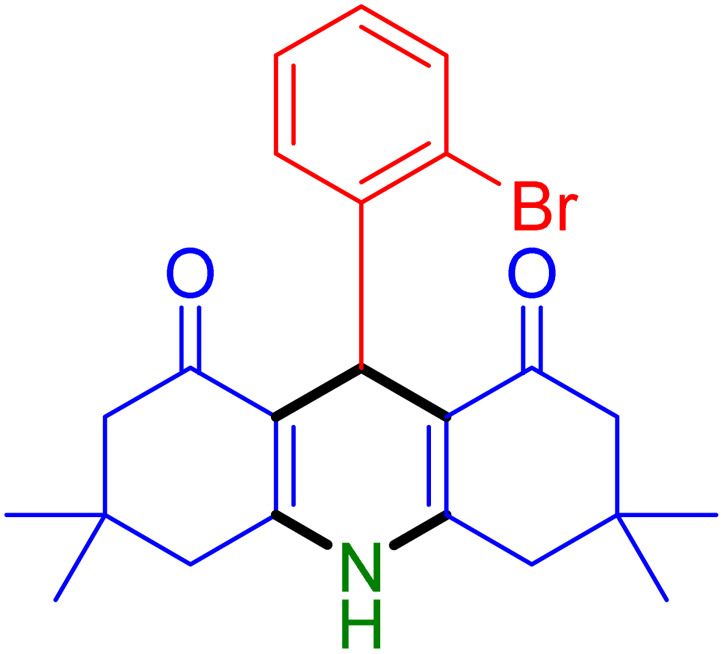	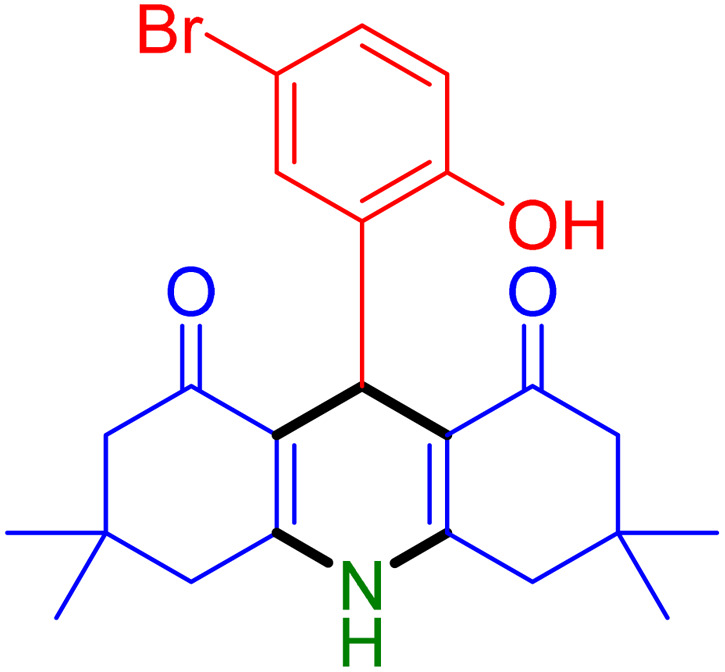
Entry 7: 4g, 87%	Entry 8: 4h, 81%	Entry 9: 4i, 80%
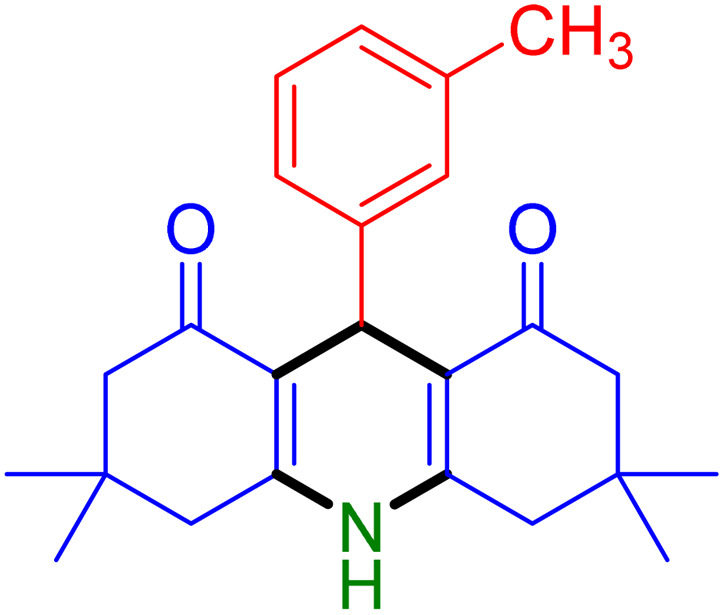	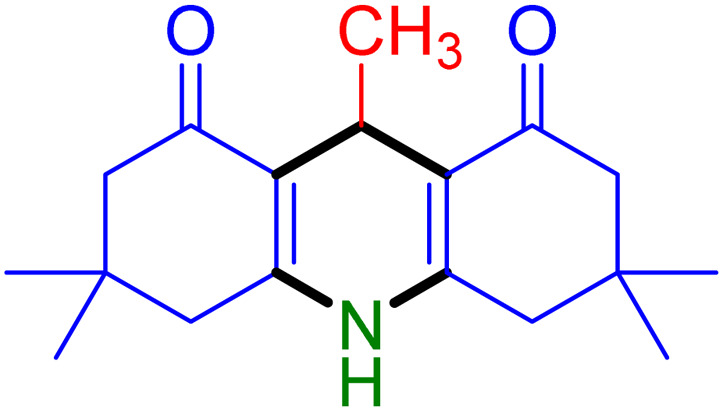	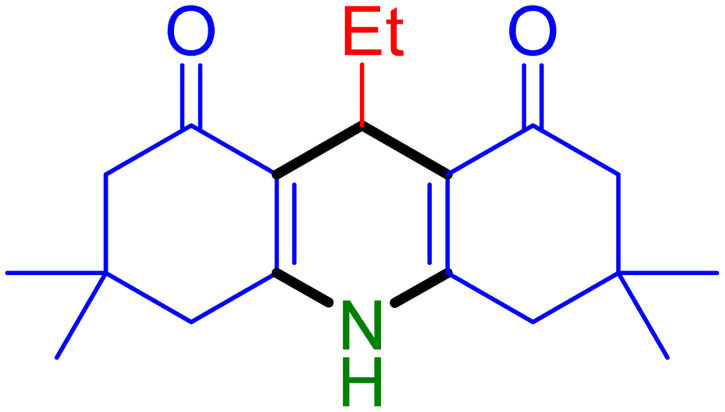
Entry 10: 4j, 80%	Entry 11: 4k, 74%^c^	Entry 12: 4l, 70%^c^
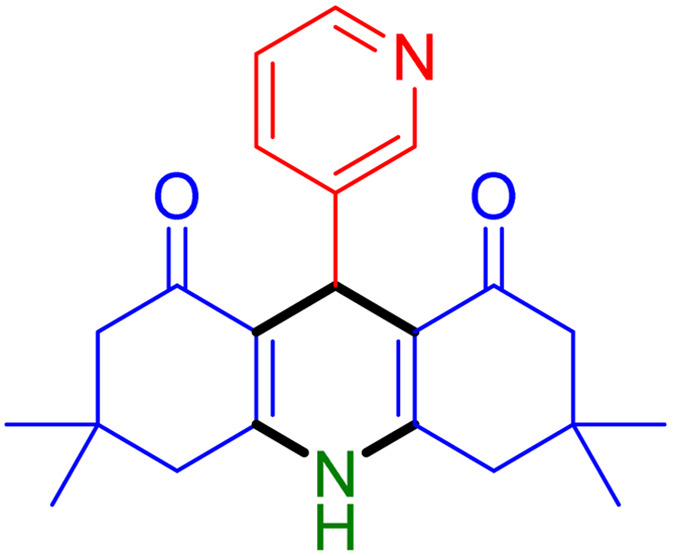	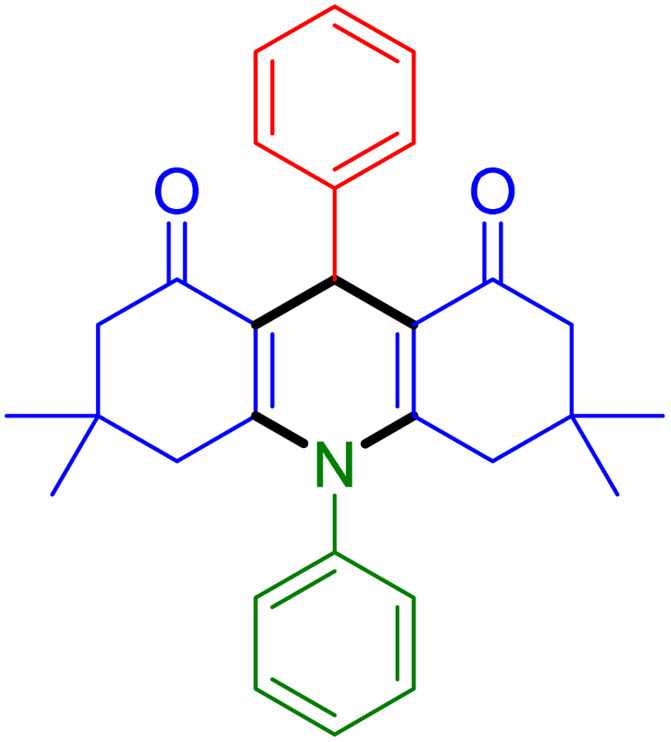	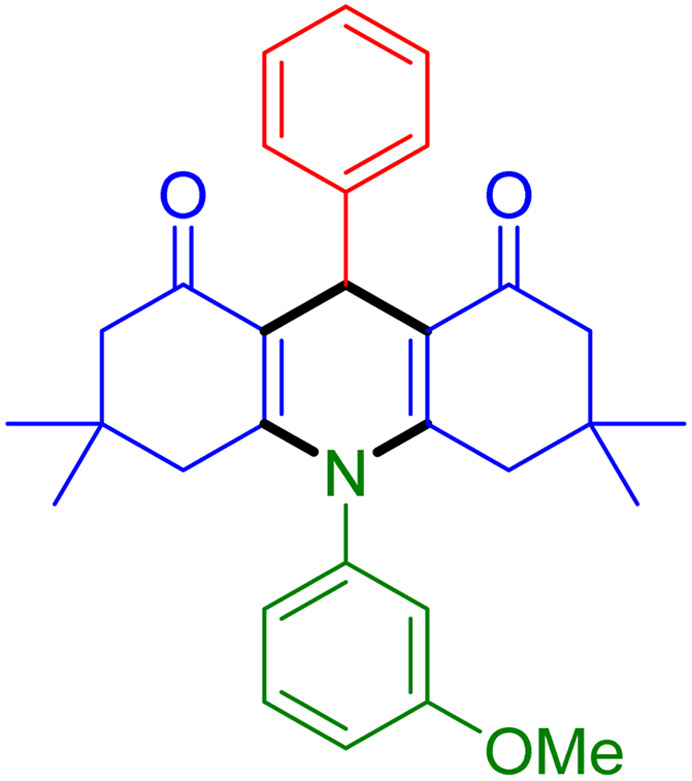
Entry 13: 4m, 83%	Entry 14: 4n, 84%	Entry 15: 4o, 84%
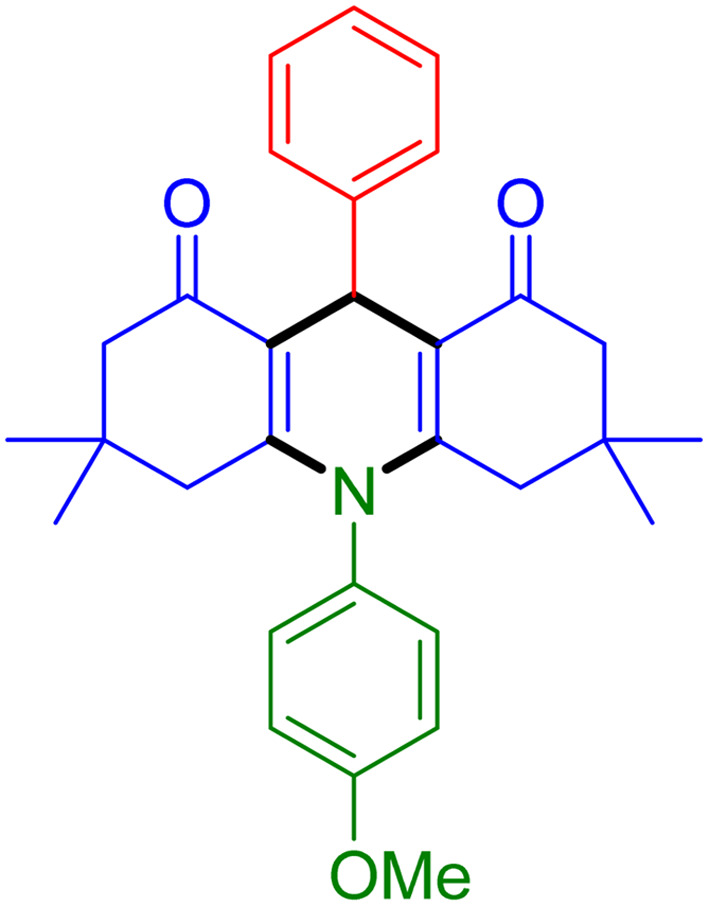	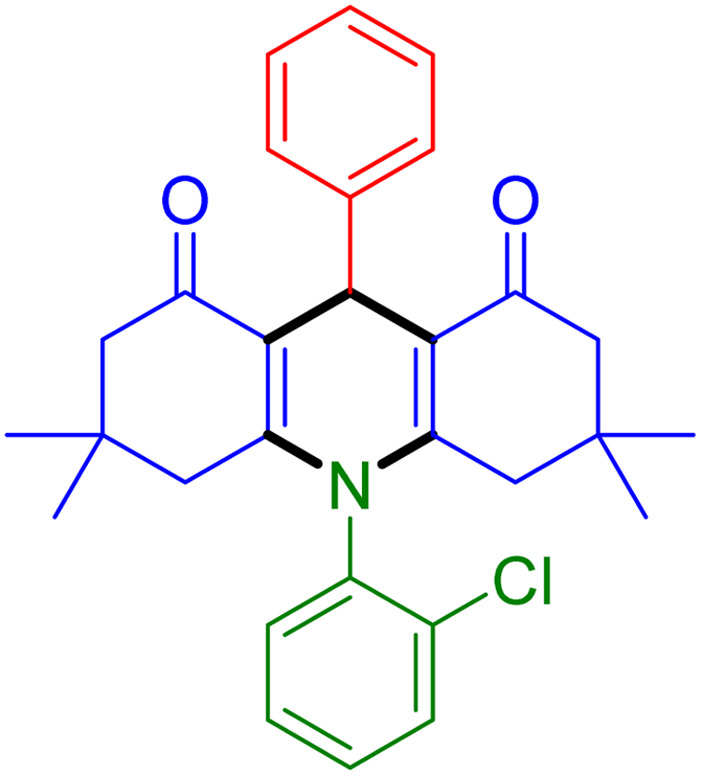	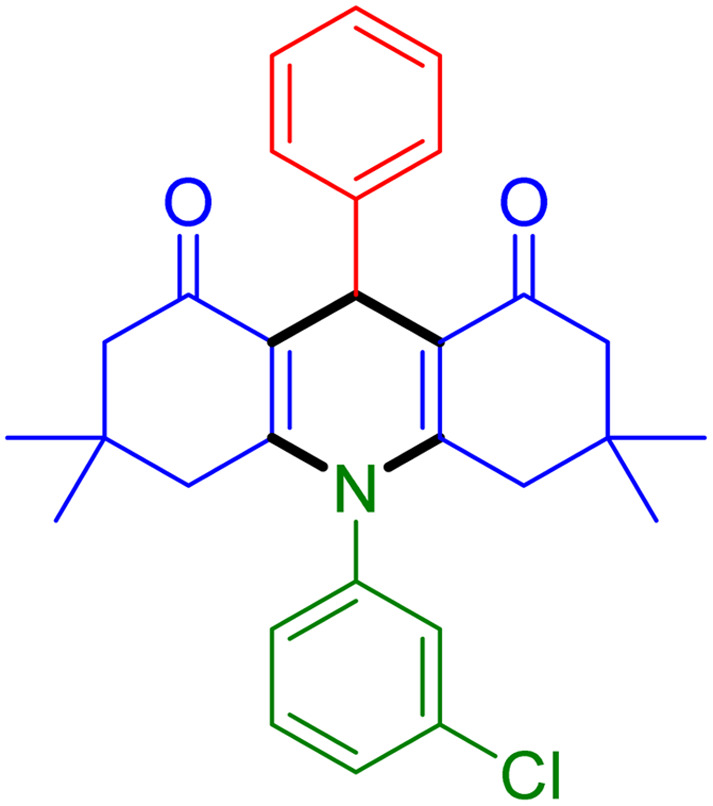
Entry 16: 4p, 87%	Entry 17: 4q, 73%	Entry 18: 4r, 77%
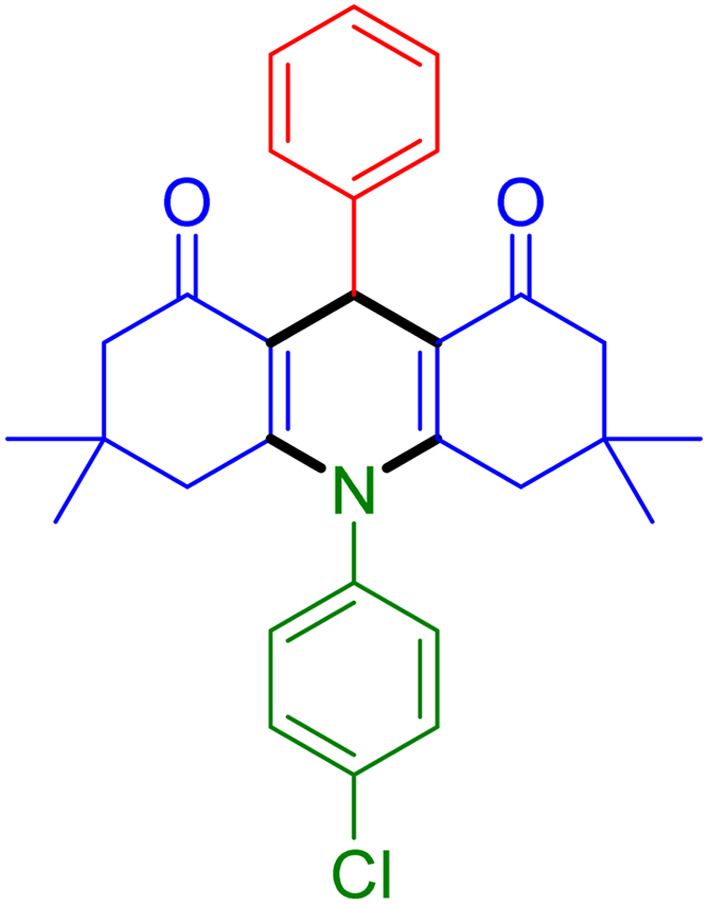	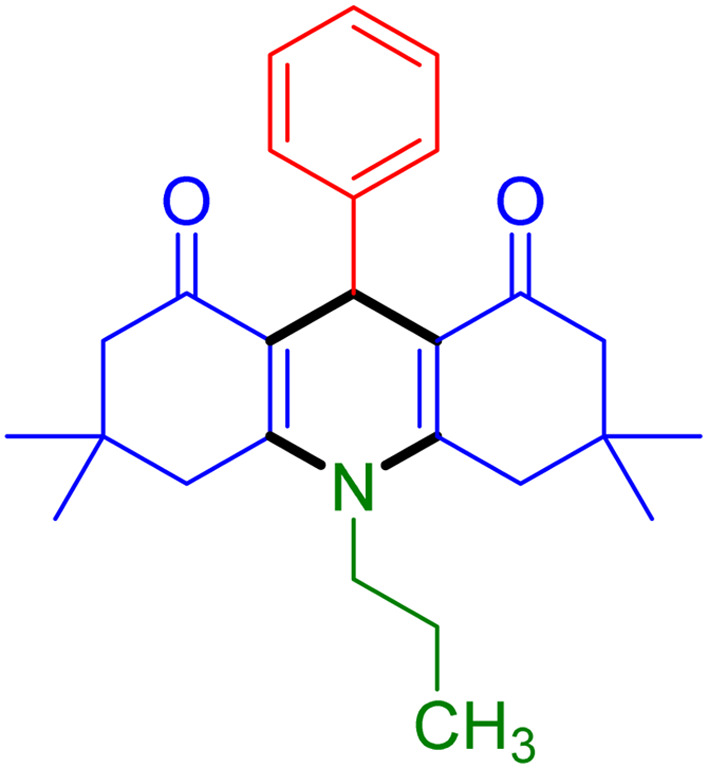	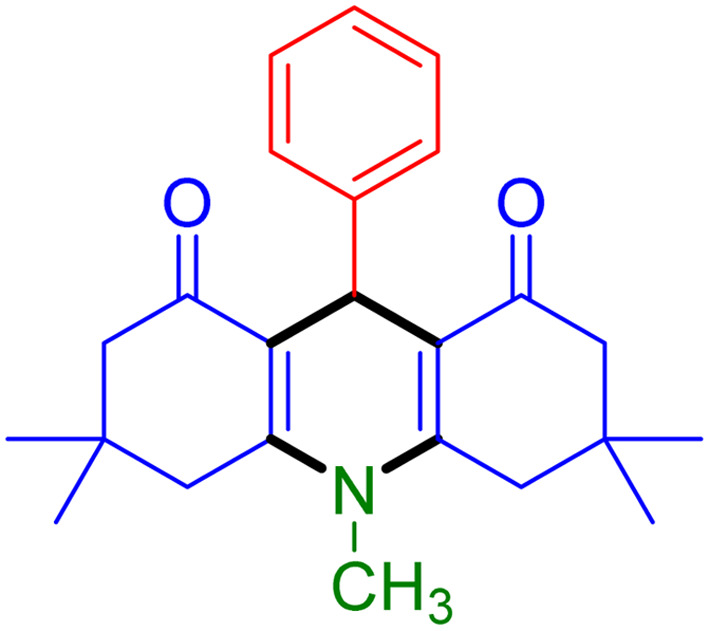
Entry 19: 4s, 78%	Entry 20: 4t, 94%	Entry 21: 4u, 96%
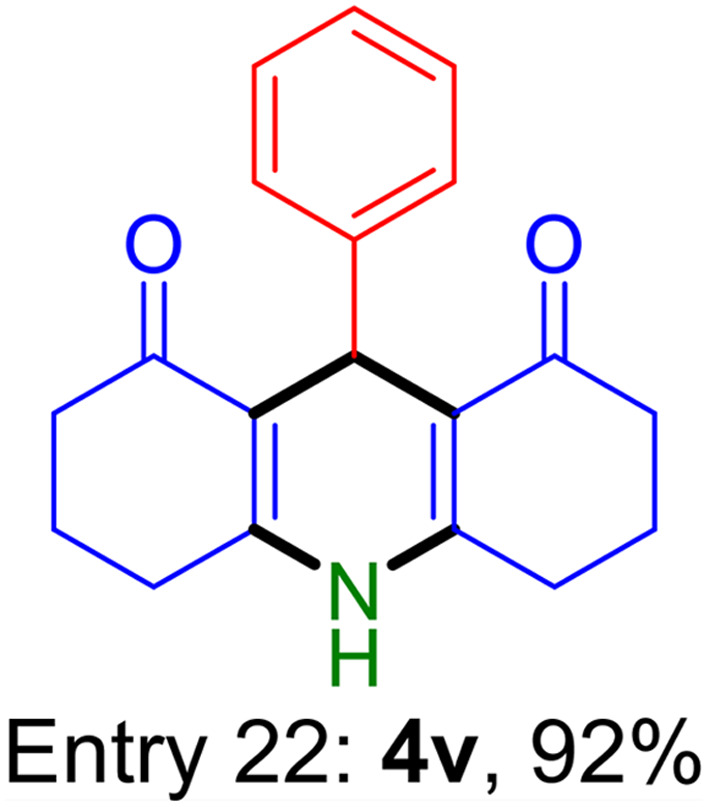	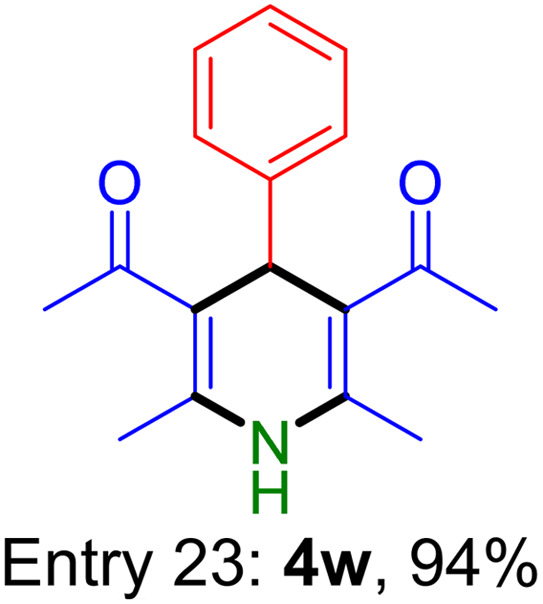	
Entry 22: 4v, 92%	Entry 23: 4w, 94%	

a Experimental conditions: aldehyde (1.0 mmol), dimedone (2.0 mmol), and ammonium acetate/amines (1.1 mmol), catalyst (5.0 mg), in water (2.0 mL) at 60 °C.

b Isolated yield.

c The reaction was performed in a sealed tube.

Substituted aldehydes at the *ortho* position resulted in lower yields when compared to their *para*- or *meta*-substituted counterparts, potentially due to the steric hindrance (entries 2 and 5 compared to entries 5 and 7). Electron-rich aldehydes such as 3-methylbenzaldehyde were made to react under the stipulated conditions to afford the desired product 4j in reasonably good yield (entry 10). The scope of the present method was also extended to more challenging aliphatic aldehydes. When dimedone 1, acetaldehyde, and ammonium acetate were added at once in a sealed tube and stirred at 60 °C, the desired product 4k was obtained in moderate yield (74%, entry 11). When propanal was employed, the respective product 4l was obtained in 70% yield (entry 12). As a heterocyclic aldehyde, pyridine-3-carbaldehyde was also found to be compatible with this process and gave the corresponding product 4m with an 83% yield (entry 13).

To further extend the adaptability of the catalytic system, different primary aromatic and aliphatic amines containing a variety of substituents, such as chloro and methoxy, were subjected to optimized reaction conditions to give the corresponding products. Electron-rich anilines modulated the performance of the process. For example, 3- and 4-methoxyaniline afforded the expected products 4o and 4p in 84 and 87% yield, respectively (entries 15 and 16). Sterically demanding substrates such as 2-chloroaniline slightly reduce the product yield (entry 17). Much to our satisfaction, it was discovered that the reaction conditions were mild enough to enable the incorporation of halogenated anilines (entries 17–19). Furthermore, propylamine as a primary aliphatic amine was also found to be adept at efficiently furnishing the desired product 4t in excellent yield (94%, entry 20). Methylamine, another aliphatic amine, also provided the corresponding acridine-1,8-dione 4u in excellent yield (entry 21). In addition to dimedone, other β-diketones such as 1,3-cyclohexanedione and acetylacetone were also applied to this protocol, and the desired products 4v and 4w were obtained in excellent yields (Table 2, entries 22 and 23).

**Fig. 9:**
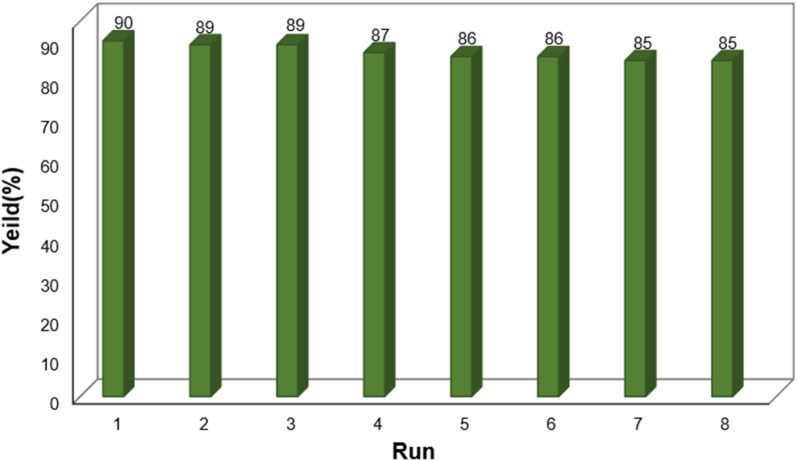
Recyclability of Fe_3_O_4_@SiO_2_-NH-GA-[(CH_2_)_4_-SO_3_H]_3_ nanocatalyst in the synthesis of acridine-1,8-dione.

One of the key factors that determines the effectiveness and economic viability of a heterogeneous catalyst is its reusability. Using a reusable catalyst in industrial processes can lead to reduced production costs, minimized waste, maintained consistency, and improved sustainability.^38^ Consistent with these benefits, the reusability of the final catalyst was attempted during the reaction of dimedone, benzaldehyde, and ammonium acetate. While the catalyst was reusable up to eight times with negligible impact on the product yield, after separation with an external magnetic field and washes with H_2_O and EtOH, a gradual decrease in the catalytic activity was observed in the ninth and tenth runs (Fig. 9).

To quantify the environmental sustainability of our catalytic system, some key metrics in green chemistry, such as the environmental factor (E-factor)^39^ and atom economy,^40^ were evaluated. These metrics measure the overall environmental impact and the efficiency of a chemical process when a green chemistry improvement has been made to the process. Using the Fe_3_O_4_@SiO_2_-NH-GA-[(CH_2_)_4_-SO_3_H]_3_-catalyzed reaction between dimedone, benzaldehyde, and ammonium acetate as an example, the E-factor of the process was 0.5 kg kg^−1^. The atom economy, another essential metric in the realm of green chemistry, was also calculated. The atom economy of the presented catalytic system was 75.4%. Reaction mass efficiency (RME)^41^ and process mass intensity (PMI)^13*b*^ are other important factors widely used to evaluate the “greenness” of chemical transformations. Under the optimized conditions, the calculation of the percentage of reaction mass efficiency gave a value of 67%, indicating the cleanliness of the process. A nearly ideal value of process mass intensity (PMI) was also obtained for this catalytic system (1.51). Ecoscale,^42^ which is based on economical and ecological parameters, stands as another crucial indicator within the domain of green chemistry for evaluating reactions. This methodology has 63.0 scores in terms of the ecoscale, which is demonstrative of an acceptable synthesis. The obtained data for the present catalytic system shows a nice combination between the ecoscale score and the E-factor. The other parameters, such as atom economy, RME, and PMI, advocate that this methodology is a clean and green synthetic route for the synthesis of acridine-1,8-diones. The calculated data is given in the ESI file.†

**Fig. 10:**
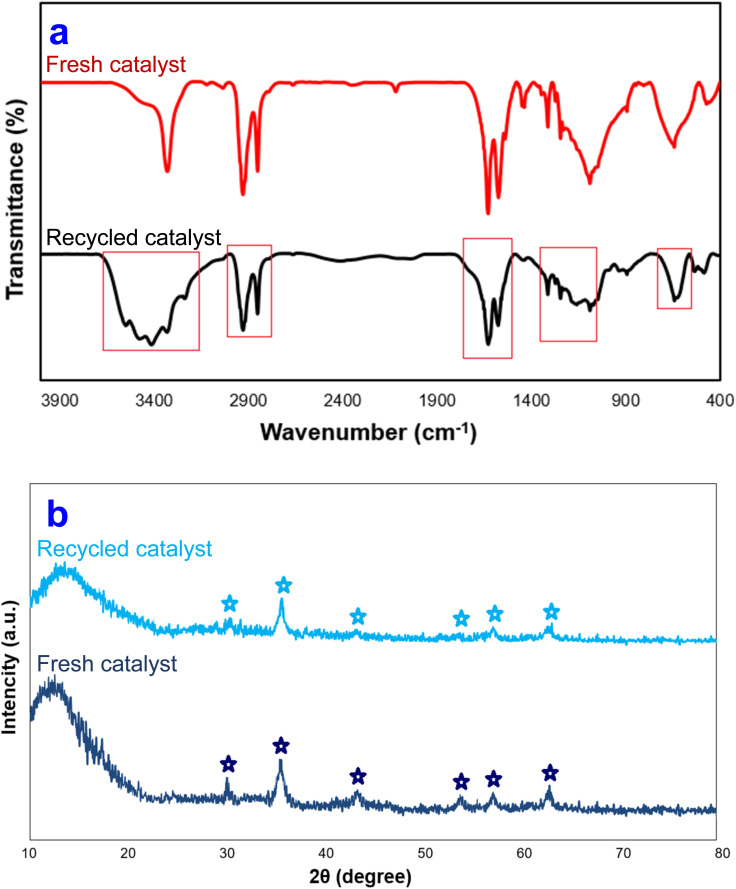
(a) FT-IR and (b) XRD diagram of Fe_3_O_4_@SiO_2_-NH-GA-[(CH_2_)_4_-SO_3_H]_3_ MNPs after eight reaction cycles.

To determine the fate of the Fe_3_O_4_@SiO_2_-NH-GA-[(CH_2_)_4_-SO_3_H]_3_ catalyst used in these condensation reactions, the residual catalyst was separated from the reaction mixture by an external magnet and characterized after being reused. Compared to the fresh catalyst, the FT-IR spectrum (KBr) of the catalyst isolated at the conclusion of the reaction shows a broader signal at about *ν* = 3150–3400 cm^−1^ (O–H stretch), as well as a decrease in the intensity of diagnostic signals (Fig. 10a). The spent Fe_3_O_4_@SiO_2_-NH-GA-[(CH_2_)_4_-SO_3_H]_3_ was also characterized by the X-ray diffraction method. According to the XRD analysis presented in Fig. 10b, the X-ray diffraction patterns of the initial and used Fe_3_O_4_@SiO_2_-NH-GA-[(CH_2_)_4_-SO_3_H]_3_ catalysts exhibited negligible changes, indicating that all peaks in the recovered nanocomposite remained unchanged during the reaction process. These findings demonstrated that the active sites in the catalyst were preserved without any substantial modifications, thereby implying their retention and stability upon reuse.

**Fig. 11:**
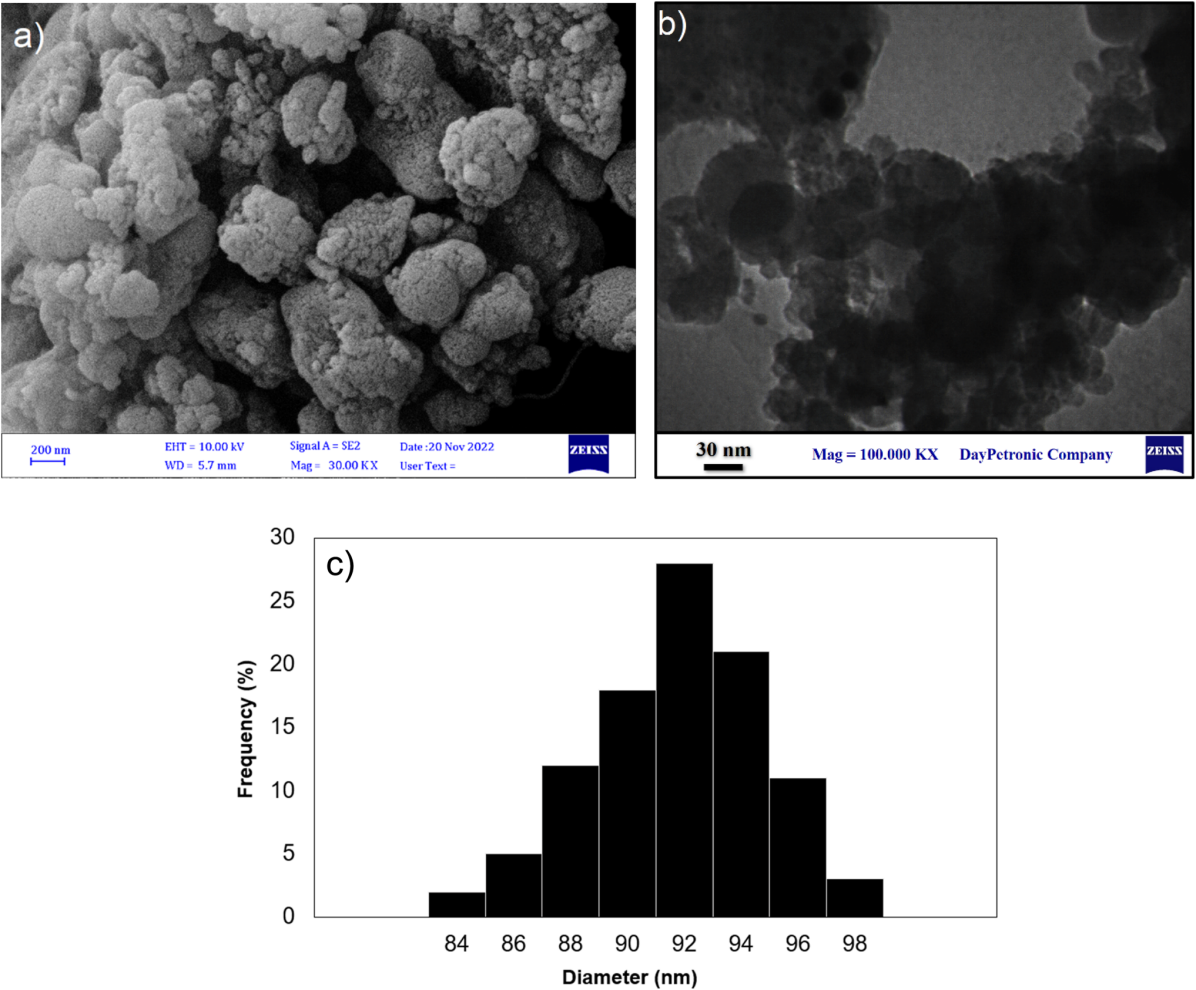
(a) FE-SEM, (b) TEM image and (c) DLS of Fe_3_O_4_@SiO_2_-NH-GA-[(CH_2_)_4_-SO_3_H]_3_ MNPs after eight reaction cycles.

FE-SEM and TEM analyses were conducted on the reused catalyst after the 8th run, as shown in Fig. 11. The FE-SEM and TEM images (Fig. 11a and b) revealed that the morphology of the catalyst exhibits evidence of slight agglomeration during the condensation reaction. Moreover, DLS analysis of the retrieved nanocomposite (Fig. 11c) obviously showed an average of 92 nm in size distribution. Hence, the observed decline in catalyst activity and increase in the average size of the catalyst can be attributed to this agglomeration process.

**Scheme 2:**
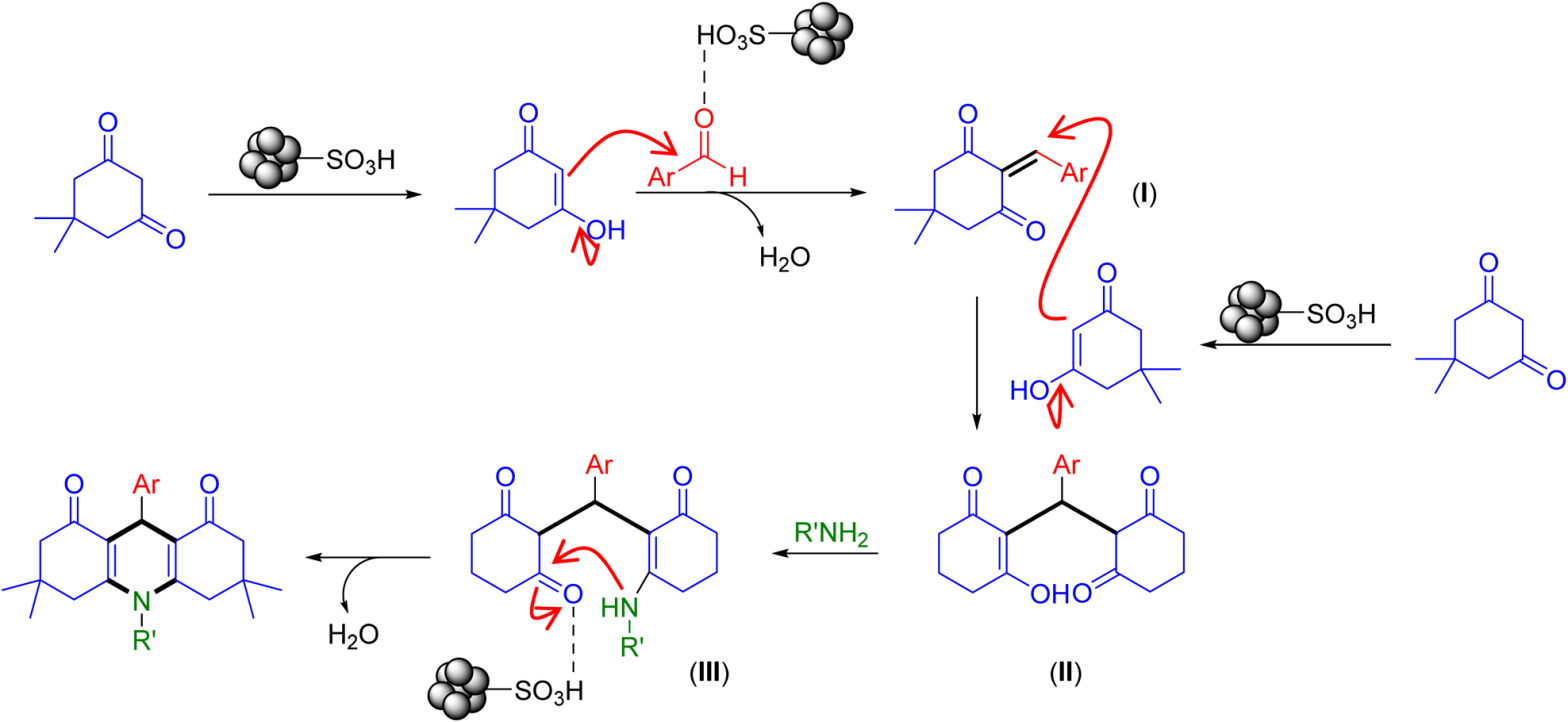
Plausible mechanistic pathway for the construction of acridine-1,8-diones catalyzed by the Fe_3_O_4_@SiO_2_-NH-GA-[(CH_2_)_4_-SO_3_H]_3_ MNPs.

Based on the results and the literature survey,^15*a*,43^ the possible catalytic route for the synthesis of acridine-1,8-diones is shown in Scheme 2.

Initially, the carbonyl groups of both dimedone and aldehyde get activated by the catalyst Fe_3_O_4_@SiO_2_-NH-GA-[(CH_2_)_4_-SO_3_H]_3_ to generate the enolic form of dimedone (as a nucleophile) and protonated aldehyde. These two activated compounds react *via* Knoevenagel condensation to produce chalcone intermediate (I). Next, the Michael addition of activated dimedone to the chalcone (I) affords the Michael adduct (II). Subsequent nucleophilic addition of amines gives the intermediate (III), which undergoes simultaneous dehydration followed by intramolecular cyclization and imine-enamine tautomerization to form the corresponding acridines.

To check the merit of the present work, we conducted a comparative study of the catalytic efficiency of Fe_3_O_4_@SiO_2_-NH-GA-[(CH_2_)_4_-SO_3_H]_3_ with several other reported acidic catalytic systems for the synthesis of acridine-1,8-diones 4a (Table 3).

**Table 3:** Comparative study of Fe_3_O_4_@SiO_2_-NH-GA-[(CH_2_)_4_-SO_3_H]_3_ for the one-pot, three-component synthesis of 4a

Entry	Catalyst	Condition	Yield (%)	Recovered run	Ref.
1	Fe_3_O_4_–TiO_2_–SO_3_H	Solvent-free/110 °C, 50 min	95	5	44
2	Fe_3_O_4_@TiO_2_@O_2_PO_2_(CH_2_)_2_NHSO_3_H	Solvent-free/90 °C, 20 min	90	5	45
3	Nano-ZrO_2_-SO_3_H	Solvent-free/100 °C, 50 min	89	5	46
4	Fe_3_O_4_@SiO_2_-NH-GA-[(CH_2_)_4_-SO_3_H]_3_	H_2_O/60 °C, 60 min	92	8	This work

These comparative results demonstrate the distinct advantage of utilizing heterogeneous Fe_3_O_4_@SiO_2_-NH-GA-[(CH_2_)_4_-SO_3_H]_3_ over the currently employed methods (based on reaction conditions, yield, and recovered runs).

## Conclusion

To sum up, a novel Fe_3_O_4_@SiO_2_ core/shell functionalized by sulfonated gallic acid has been prepared and demonstrated to be a highly efficient heterogeneous catalyst for the multicomponent synthesis of acridine-1,8-dione derivatives in water and under mild conditions. Under the optimal reaction conditions, the newly developed catalytic system tolerated various electronics and bulky aldehydes and amines. Hence, the combination of the magnetite with the introduced sulfonic groups cherishes the hybrid properties of the green chemistry matrix along with the robustness of a magnetic system. The use of this catalyst has the potential to improve the efficiency, sustainability, and cost-effectiveness of the synthetic procedures of other heterocyclic compounds. This heterogeneous catalyst could be recovered by a simple external magnet and reused up to eight times with only a minor loss of its catalytic activity.

## Author contributions

Zahra Firoozi: data curation; investigation; formal analysis, writing a draft. Dariush Khalili: project administration; supervision, conceptualization, writing – review and editing. Ali Reza Sordaria: project administration; supervision, review and editing.

## Data availability

The data that support the findings of this research work are presented in the ESI† of this article.

## Conflicts of interest

The authors declare no competing financial interest.

## Supplementary Material

RA-014-D4RA00629A-s001
