# Supplementary material for: Fe3O4@SiO2 core/shell functionalized by gallic acid: a novel, robust, and water-compatible heterogeneous magnetic nanocatalyst for environmentally friendly synthesis of acridine-1,8-diones
Source: RSC Adv. 2024 Apr 3;14(16):10842–57. doi: 10.1039/d4ra00629a (PMC10990003; doi:10.1039/d4ra00629a)
Supplement: RA-014-D4RA00629A-s001 [file RA-014-D4RA00629A-s001.pdf]

Electronic Supplementary Material (ESI) for RSC Advances.
This journal is © The Royal Society of Chemistry 2024

Supplementary information

Fe_3O_4@SiO_2 core/shell functionalized by gallic acid: a novel, robust and water-compatible heterogeneous magnetic nanocatalyst for environmentally friendly synthesis of acridine-1,8-diones

Zahra Firoozi, Dariush Khalili,* and Ali Reza Sardarian*

[a]*Department of Chemistry, College of Sciences, Shiraz University, Shiraz 71467-13565, Iran*

Table of content

Experimental section…………………………………………………………………….S2

Calculation of green chemistry metrics……………………………………………………..S2

Characterization of the products………………………………………………………..S4

Copy of FT-IR, ^{1}H-NMR and ^{13}C-NMR of products……………………………..…......…S12

References……………………………………………………………………………...S56

Experimental section

Chemical and instrument

All chemicals were purchased from Merck, Acros, and Sigma-Aldrich and used without further purification. The power and oscillation of the used ultrasonic device in catalyst synthesis was 120 (V) and 40 (kHz) and the final catalyst was characterized as follows: The fourier transform infrared spectrometer (FT-IR) model Shimadzu FT-IR 8300 was applied to FT-IR measurement using KBr pellet in the range of 400 to 4000 wavenumbers/cm^{-1}. Also, the X-ray diffraction (XRD) patterns were recorded by a GNR (Italy) XRD explorer X-ray diffractometer using CuKα radiation (λ = 1.54178 Å) with a 2θ scan range of 10° to 80°. Moreover, the presence of elements in the magnetic samples was proved by energy dispersive X-ray spectrometry (EDX) attached to a Philips scanning electron microscope (SEM), Transmission electron microscope (TEM) images were taken on a ZEISS EM10C-100KV microscope, and field emission scanning electron microscope (FE-SEM) images were taken on a ZEISS SIGMA VP microscope, Dynamic light scattering (DLS) measurements were performed on a HORIBA LB-550. To obtain thermal gravimetric analysis (TGA) data, a device from TA company model Q600 made in USA was used and vibrating sample magnetometer (VSM) measurements were analyzed using BHV-55 model vibrating sample magnetometer. Finally, zeta potential analysis was performed on a HORIBA Z-100. The reaction progress has been checked by thin layer chromatography (TLC). The final products characterized by melting points in open capillary tubes were determined with a Büchi B-545 melting point apparatus, and nuclear magnetic resonance (NMR) spectroscopy using Devices Brucker DPX-400 spectrometer that work for ^{13}C at 101 MHz and for ^{1}H at 400 MHz and a spectrometer Brucker DPX-300 that work for ^{13}C at 75 MHz and ^{1}H at 300 Hz in pure deuterated dimethyl sulfoxide (DMSO-d_6) and deuterated chloroform ($CDCl_3$).

Calculation of green chemistry metrics

In order to assess the environmental sustainability of our catalytic system, important green chemistry metrics such as the environmental factor (E-factor), atom economy, reaction mass efficiency (RME), process mass intensity (PMI) and eco-score (scale) were scrutinized. Taking the $Fe_3O4@SiO_2$-NH-GA-$[(CH_2)_4$-$SO_3H]_3$-catalyzed reaction involving dimedone, benzaldehyde, and ammonium acetate as a model reaction, these metrics were obtained as follows:

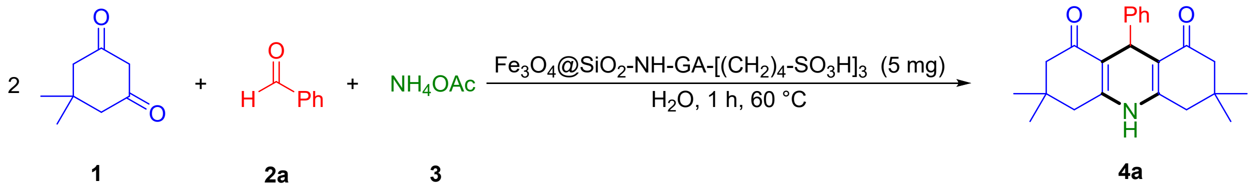

Compound code	1	2a	3	4a
M.W. (g/mol)	140	106	77	349
M.W. (mg)	2×140	106	84.7	349

The total mass of reactants = 470.7

Obtained product = 349 × 0.9 = 314.1

Environmental factor (E-factor):

E-factor = Amount of waste /Amount of product

The Amount of waste = (total mass of raw materials - the total mass of product)

The Amount of waste = (470.7 – 314.1) = 156.6

E-factor = 156.6 /314.1 = 0.50 $KgKg^{-1}$

$$\% \text{ Atom economy} = 100 \times \frac{Molecular\ weight\ of\ the\ desired\ product}{Molecular\ weight\ all\ of\ reactants}$$

$$\% \text{ Atom economy} = 100 \times \frac{349}{280 + 106 + 77} = 75.4\%$$

Reaction mass efficiency (RME) = $\frac{mass\ of\ product}{\Sigma\ mass\ of\ stoichiometric\ reactants} \times 100$

Reaction mass efficiency (RME) = $\frac{314.1}{470.7} \times 100$ = 67%

Process mass intensity (PMI) = $\frac{\Sigma\ (mass\ of\ stoichiometric\ reactants\ +\ solvent}{mass\ of\ product}$

PMI = $\frac{470.7 + 2}{314.1}$ = 1.51

Ideal value of PMI = E-factor + 1 = 0.50 + 1 =1.50

E-score has been calculated for the reaction based on the following 6 parameters below (See Beilstein Journal of Organic Chemistry 2006, 2, 3.)

Entry	Parameter	Values	Penalty Points
1	Yield	100-90/2	5
2	Cost of reactants	Inexpensive	0
3	Safety of reactants	5+5+5+5	20
4	Technical setup	Common setup	0
5	Temperature /time	60 °C, <1 h	2
6	Workup and purification	Classical chromatography	10
Total penalty points			37

Eco-Score = 100 - the sum of individual penalties = 100-37 = 63

Eco-scale from 0 to 100 using the following scores: > 75, excellent; > 50, acceptable; and < 50, inadequate.

Characterization of the products

3,3,6,6-tetramethyl-9-phenyl-3,4,6,7,9,10-hexahydroacridine-1,8(2H,5H)-dione (4a) (Table 4, entry 1)

White solid; Yield = 92%, M.P. 272-275 ˚C (Lit. 274-276 °C [1]); IR (KBr): ū (cm^{-1}) = 3280, 3196, 3066, 2960, 1644, 1610, 1486, 1224. ^{1}H NMR (400 MHz, DMSO-d_6) δ (ppm): 9.40 (s, 1H, N-*H*), 7.23-7.08 (m, 5H, Ar*H*), 4.88 (s, 1H, C*H*Ar), 2.53 (d, *J* = 16.96 Hz, 2H, CH_2), 2.40 (d, *J* = 17.12 Hz, 2H, CH_2), 2.25 (d, *J* = 16.12 Hz, 2H, CH_2), 2.06 (d, *J* = 16.32 Hz, 2H, CH_2), 1.08 (s, 6H, 2CH_3), 0.93 (s, 6H, 2CH_3). ^{13}C NMR (101MHz, DMSO-d_6) δ (ppm): 26.4, 29.0, 32.1, 32.7, 50.1, 111.4, 125.4, 127.5, 127.5, 147.1, 149.3, 194.3.

3,3,6,6-tetramethyl-9-(2-nitrophenyl)-3,4,6,7,9,10-hexahydroacridine-1,8(2H,5H)-dione (4b) (Table 4, entry 2)

Light yellow solid; Yield = 88%, M.P. 294-296 °C(Lit. 295-297 °C[2]); IR (KBr): ū (cm^{-1}) = 3424, 3286, 3204, 2958, 1612, 1370, 1224. ^{1}H NMR (400 MHz, $CDCl_3$) δ (ppm): 9.58 (s, 1H, N-*H*), 7.60-7.32 (m, 4H, Ar*H*), 5.64 (s, 1H, C*H*Ar), 2.29 (d, *J* = 8.68 Hz, 2H, CH_2), 2.20 (d, *J* = 16.60 Hz, 2H, CH_2), 2.13 (d, *J* = 16.16 Hz, 2H, CH_2), 2.06 (d, *J* = 16.24 Hz, 2H, CH_2), 1.06 (s, 6H, 2CH_3), 0.91 (s, 6H, 2CH_3). ^{13}C NMR (101MHz, $CDCl_3$) δ (ppm): 20.5, 22.6, 28.5, 39.8, 49.9, 67.1, 113.2, 127.7, 129.2, 129.9, 131.3, 139.9, 148.0, 148.0, 195.1.

3,3,6,6-tetramethyl-9-(3-nitrophenyl)-3,4,6,7,9,10-hexahydroacridine-1,8(2H,5H)-dione (4c) (Table 4, entry 3)

Light yellow solid; Yield = 94%, M.P. 293-295 °C(Lit. 291-293 °C[1]); IR (KBr): ū (cm^{-1}) = 3272, 3186, 3066, 2960, 1646, 1610, 1488, 1346, 1224. ^{1}H NMR (400 MHz, DMSO-d_6) δ (ppm): 9.50 (s, 1H, N-*H*), 7.49-7.98 (m, 4H, Ar*H*), 4.92 (s, 1H, C*H*Ar), 2.49 (d, *J* = 16.64 Hz, 2H, CH_2), 2.37 (d, *J* = 17.08 Hz, 2H, CH_2), 2.21 (d, *J* = 16.16 Hz, 2H, CH_2), 2.00 (d, *J* = 16.12 Hz, 2H, CH_2), 1.02 (s, 6H, 2CH_3), 0.86 (s, 6H, 2CH_3). ^{13}C NMR (101MHz, DMSO-d_6) δ (ppm): 26.3, 29.0, 32.1, 33.4, 49.9, 110.5, 120.7, 121.9, 134.4, 147.3, 149.1, 150.0, 194.4.

3,3,6,6-tetramethyl-9-(4-nitrophenyl)-3,4,6,7,9,10-hexahydroacridine-1,8(2H,5H)-dione (4d) (Table 4, entry 4)

Light yellow solid; Yield = 96%, M.P. 299-301 °C(Lit. 297-299 °C[1]); IR (KBr): ū (cm^{-1}) = 3386, 3074, 2960, 1644, 1480, 1342, 1222. ^{1}H NMR (400 MHz, DMSO-d_6) δ (ppm): 9.50 (s, 1H, N-*H*), 8.08 (d, *J* = 8.60 Hz, 2H, Ar*H*), 7.42 (d, *J* = 8.68 Hz, 2H , Ar*H*), 4.90 (s, 1H, C*H*Ar), 2.49 (d, *J* = 17.00 Hz, 2H, CH_2), 2.34 (d, *J* = 17.08 Hz, 2H, CH_2), 2.20 (d, *J* = 16.16 Hz, 2H, CH_2), 1.99 (d, *J* = 16.36 Hz, 2H, CH_2), 1.01 (s, 6H, 2CH_3), 0.86 (s, 6H, 2CH_3). ^{13}C NMR (101MHz, DMSO-d_6) δ (ppm): 27.1, 29.4, 32.7, 34.4, 50.5, 112.8, 123.4, 129.0, 130.9, 147.8, 153.7, 195.1.

9-(2-chlorophenyl)-3,3,6,6-tetramethyl-3,4,6,7,9,10-hexahydroacridine-1,8(2H,5H)-dione (4e) (Table 4, entry 5)

Light yellow solid; Yield = 85%, M.P. 292-294 ^{o}C(Lit. 290-299 ^{o}C[4]); IR (KBr): ῡ (cm^{-1}) = 3280, 3202, 3072, 2954, 1636, 1608, 1486, 1224, 750. ^{1}H NMR (400 MHz, DMSO-d_6) δ (ppm): 9.35 (s, 1H, N-*H*), 7.29 (d, *J* = 7.08 Hz, 2H, Ar*H*), 7.20-7.13 (m, 2H, Ar*H*), 7.05 (d, *J* = 7.44 Hz, 2H, Ar*H*), 5.08 (s, 1H, C*H*Ar), 2.45 (d, *J* = 17.00 Hz, 2H, CH_2), 2.28 (d, *J* = 17.00 Hz, 2H, CH_2), 2.15 (d, *J* = 16.16 Hz, 2H, CH_2), 1.93 (d, *J* = 16.12 Hz, 2H, CH_2), 1.01 (s, 6H, 2CH_3), 0.87 (s, 6H, 2CH_3). ^{13}C NMR (101MHz, DMSO-d_6) δ (ppm): 26.3, 29.1, 31.9, 32.9, 50.2, 110.7, 126.1, 126.9, 129.0, 131.9, 132.2, 144.0, 149.6, 194.1.

9-(3-chlorophenyl)-3,3,6,6-tetramethyl-3,4,6,7,9,10-hexahydroacridine-1,8(2H,5H)-dione (4f) (Table 4, entry 6)

Light yellow solid; Yield = 87%, M.P. 292-294 ^{o}C(Lit. 290 ^{o}C[4]); IR (KBr): ῡ (cm^{-1}) = 3416, 3182, 3064, 2962, 1620, 1616, 1490, 1220, 694. ^{1}H NMR (400 MHz, $CDCl_3$) δ (ppm): 9.50 (s, 1H, N-*H*), 7.22 (s, 1H, Ar*H*), 7.22-7.08 (m, 1H, Ar-*H*), 7.05 (t, *J* = 7.80 Hz, 1H, Ar*H*), 7.00-6.97 (m, 1H, Ar*H*), 5.00 (s, 1H, C*H*Ar), 2.25 (d, *J* = 16.88 Hz, 2H, CH_2), 2.19 (d, *J* = 4.92 Hz, 2H, CH_2), 2.15 (d, J = 5.52 Hz, 2H, CH_2), 2.10 (d, *J* = 16.36 Hz, 2H, CH_2), 1.01 (s, 6H, 2CH_3), 0.90 (s, 6H, 2CH_3). ^{13}C NMR (101MHz, $CDCl_3$) δ (ppm): 27.1, 29.5, 32.6, 40.8, 50.7, 112.8, 126.2, 126.6, 128.0, 129.1, 133.7, 148.5, 148.8, 195.67.

9-(4-chlorophenyl)-3,3,6,6-tetramethyl-3,4,6,7,9,10-hexahydroacridine-1,8(2H,5H)-dione (4g) (Table 4, entry 7)

White solid; Yield = 87%, M.P. 294-296 ^{o}C(Lit. 298-300 ^{o}C[5]); IR (KBr): ῡ (cm^{-1}) = 3552, 3478, 3408, 2960, 1644, 1618, 1488, 1222, 844. ^{1}H NMR (400 MHz, DMSO-d_6) δ (ppm): 9.38 (s, 1H, N-*H*), 7.23 (d, *J* = 8.40 Hz, 2H, Ar*H*), 7.15 (d, *J* = 8.48 Hz, 2H, Ar*H*), 4.78 (s, 1H, C*H*Ar), 2.46 (d, *J* = 17.04 Hz, 2H, CH_2), 2.32 (d, *J* = 17.04 Hz, 2H, CH_2), 2.18 (d, *J* = 16.12 Hz, 2H, CH_2), 1.98 (d, *J* = 16.08 Hz, 2H, - CH_2), 1.01 (s, 6H, 2CH_3), 0.86 (s, 6H, 2CH_3). ^{13}C NMR (101MHz, DMSO-d_6) δ (ppm): 27.1, 29.5, 39.6, 40.9, 50.7, 113.2, 128.1, 129.4, 131.5, 145.0, 148.2, 195.6.

9-(2-bromophenyl)-3,3,6,6-tetramethyl-3,4,6,7,9,10-hexahydroacridine-1,8(2H,5H)-dione (4h) (Table 4, entry 8)

White solid; Yield = 81%, M.P. 255-257 ^{o}C(Lit. 252-254 ^{o}C[6]); IR (KBr): ῡ (cm^{-1}) = 3276, 3182, 3064, 2962, 1640, 1612, 1486, 1222, 560. ^{1}H NMR (400 MHz, DMSO-d_6) δ (ppm): 9.39, (s, 1H, N-*H*), 7.23-7.09 (m, 4H, Ar*H*), 4.80 (s, 1H, C*H*Ar), 2.46 (d, *J* = 21.12 Hz, 2H, CH_2), 2.35 (d, *J* = 17.04

Hz, 2H, CH_2), 2.19 (d, J = 16.08 Hz, 2H, CH_2), 2.01 (d, J = 16.00 Hz, 2H, CH_2), 1.01 (s, 6H, 2CH_3), 0.87 (s, 6H, 2CH_3). ^{13}C NMR (101MHz, DMSO-d_6) δ (ppm): 26.3, 29.0, 32.1, 33.0, 50.0, 110.8, 125.4, 126.1, 127.5, 129.5, 132.1, 149.3, 149.6, 194.4.

9-(5-bromo-2-hydroxyphenyl)-3,3,6,6-tetramethyl-3,4,6,7,9,10-hexahydroacridine-1,8(2H,5H)-dione (4i) (Table 4, entry 9)

Light yellow solid; Yield = 80%, M.P. 238-240 °C(Lit. 235-238 °C[3]); IR (KBr): ū (cm^{-1}) = 3512, 3480, 3416, 2958, 1616, 1604, 1480, 1232, 626. ^{1}H NMR (300 MHz, DMSO-d_6) δ (ppm): 9.69 (s, 1H, O-H), 9.64 (s, 1H, N-H), 7.07 (d, J = 6.84 Hz, 1H, , ArH), 6.98 (s, 1H, ArH), 6.65 (d, J = 10.32 Hz, 1H, ArH), 4.79 (s, 1H, CHAr), 2.48 (d, J = 12.57 Hz, 2H, CH_2), 2.37 (d, J = 17.10 Hz, 2H, CH_2), 2.24 (d, J = 16.20 Hz, 2H, CH_2), 2.05 (d, J = 16.08 Hz, 2H, CH_2), 1.00 (s, 6H, 2CH_3), 0.91 (s, 6H, 2CH_3). ^{13}C NMR (75MHz, DMSO-d_6) δ (ppm): 26.7, 29.5, 32.6, 37.7, 50.2, 110.8, 113.4, 113.7, 119.6, 130.1, 131.9, 140.5, 143.9, 151.7, 153.7, 196.2.

3,3,6,6-tetramethyl-9-(m-tolyl)-3,4,6,7,9,10-hexahydroacridine-1,8(2H,5H)-dione (4j) (Table 4, entry 10)

Yellow solid; Yield = 80%, M.P. 214-217 °C(Lit. 211-213 °C[7]); IR (KBr): ū (cm^{-1}) = 3278, 3182, 3066, 2956, 1642, 1604, 1488, 1220. ^{1}H NMR (400 MHz, DMSO-d_6) δ (ppm): 9.33 (s, 1H, N-H), 7.12-6.90 (m, 4H, ArH), 4.83 (s, 1H, CHAr), 2.51 (d, J = 17.08 Hz, 2H, CH_2), 2.39 (d, J = 17.04 Hz, 2H, CH_2), 2.26 (s, 3H, CH_3), 2.23 (d, J = 16.28 Hz, 2H, CH_2), 2.05 (d, J = 16.28 Hz, 2H, -CH_2), 1.07 (s, 6H, 2CH_3), 0.93 (s, 6H, 2CH_3). ^{13}C NMR (101MHz, DMSO-d_6) δ (ppm): 19.3, 24.5, 27.2, 30.3, 30.8, 48.4, 109.6, 122.8, 124.2, 125.7, 126.6, 134.2, 145.2, 147.3, 192.5.

3,3,6,6,9-pentamethyl-3,4,6,7,9,10-hexahydroacridine-1,8(2H,5H)-dione (4k) (Table 4, entry 11)

Light yellow solid; Yield = 74%, M.P. 270-273 °C(Lit. 269-279 °C[8]); IR (KBr): ū (cm^{-1}) = 3416, 3280, 3204, 2958, 1630, 1608, 1488, 1232. ^{1}H NMR (400 MHz, $CDCl_3$) δ (ppm): 9.51 (s, 1H, N-H), 6.50 (s, 1H, CHAr), 2.27 (d, J = 14.64 Hz, 2H, CH_2), 2.18 (s, 3H, CH_3), 2.14 (d, J = 8.60 Hz, 2H, CH_2), 1.93 (d, J = 6.28 Hz, 2H, CH_2), 1.66 (d, J = 8.64 Hz, 2H, CH_2), 1.02 (s, 6H, 2CH_3), 0.90 (s, 6H, 2CH_3). ^{13}C NMR (101MHz, $CDCl_3$) δ (ppm): 21.5, 22.5, 27.0, 29.5, 41.1, 50.9, 114.5, 148.3, 195.9.

9-ethyl-3,3,6,6-tetramethyl-3,4,6,7,9,10-hexahydroacridine-1,8(2H,5H)-dione (4l) (Table 4, entry 12)

Light yellow solid; Yield = 70%, M.P. 291-293 °C(Lit. 282-283 °C[9]); IR (KBr): ῡ (cm^{-1}) =3468, 2951, 1608, 1395. ^{1}H NMR (400 MHz, $CDCl_3$) δ (ppm): 9.61 (s, 1H, N-*H*), 3.14 (t, *J* = 11.38 Hz, 1H,C*H*CH_2), 2.28 (d, *J* = 9.12 Hz, 2H, CH_2), 2.20 (d, *J* = 12.64 Hz, 2H, CH_2), 2.11 (d, *J* = 8.40 Hz, 2H, CH_2), 2.03 (d, *J* = 14.56 Hz, 2H, CH_2), 1.40-1.31 (m, 2H, CH_2), 1.10 (s, 6H, 2CH_3), 0.99 (s, 6H, 2CH_3), 0.89 (t, *J* = 4.86 Hz, 3H, CH_2CH_3). ^{13}C NMR (101MHz, $CDCl_3$) δ (ppm): 11.1, 24.7, 29.4, 31.8, 32.6, 40.5, 46.4, 50.0, 115.4, 125.8, 127.6, 128.0, 145.7, 150.2, 195.6.

3,3,6,6-tetramethyl-9-(pyridin-3-yl)-3,4,6,7,9,10-hexahydroacridine-1,8(2H,5H)-dione (4m) (Table 4, entry 13)

Light yellow solid; Yield = 83%, M.P. 301-303 °C(Lit. 298-300 °C[10]); IR (KBr): ῡ (cm^{-1}) = 3361, 2936, 2877, 3815, 1612, 1601, 1545, 1403. ^{1}H NMR (400 MHz, DMSO-d_6) δ (ppm): 9.48 (s, 1H, N-*H*), 7.37(d, *J* = 52.08 Hz, 2H, Ar*H*), 7.64-7.54 (m, 1H, Ar*H*), 7.33 (s, 1H, Ar*H*), 4.82 (s, 1H, C*H*Ar), 2.36 (d, *J* = 17.00 Hz, 2H, CH_2), 2.23 (d, *J* = 13.80 Hz, 2H, CH_2), 2.10 (d, *J* = 7.80 Hz, 2H, CH_2), 1.99 (d, *J* = 15.88Hz, 2H, CH_2), 1.01 (s, 6H, 2CH_3), 0.85 (s, 6H, 2CH_3). ^{13}C NMR (101MHz, DMSO-d_6) δ (ppm): 26.9, 28.9, 29.4, 31.8, 32.3, 32.6, 50.4, 110.8, 127.5, 136.8, 145.9, 148.1, 150.6, 163.9, 195.0.

3,3,6,6-tetramethyl-9,10-diphenyl-3,4,6,7,9,10-hexahydroacridine-1,8(2H,5H)-dione (4n) (Table 4, entry 14)

White solid; Yield = 84%, M.P. 250-252 °C(Lit. 253-255 °C[11]); IR (KBr): ῡ (cm^{-1}) = 3272, 3024, 2954, 1598, 1484, 1262. ^{1}H NMR (400 MHz, DMSO-d_6) δ (ppm): 7.87-7.11 (m, 10H, Ar*H*), 5.11 (s, 1H, C*H*Ar), 2.28 (d, *J* = 7.64 Hz, 2H, CH_2), 2.07 (d, *J* = 9.04 Hz, 2H, CH_2), 1.81 (d, *J* = 17.02 Hz, 2H, CH_2), 1.32 (d, *J* = 17.84 Hz, 2H, CH_2), 0.93 (s, 6H, 2CH_3), 0.77 (s, 6H, 2CH_3). ^{13}C NMR (101MHz, DMSO-d_6) δ (ppm): 25.5, 26.0, 29.2, 29.6, 49.5, 112.8, 113.0, 125.7, 127.4, 127.8, 128.2, 129.3, 138.4, 149.3, 150.2, 195.0.

10-(3-methoxyphenyl)-3,3,6,6-tetramethyl-9-phenyl-3,4,6,7,9,10-hexahydroacridine -1,8(2H,5H)-dione (4o) (Table 4, entry 15)

White solid; Yield = 84%, M.P. 260-262 °C(Lit. 184-187 °C[12]); IR (KBr): ῡ (cm^{-1}) = 3260, 2952, 1598, 1480, 1282. ^{1}H NMR (400 MHz, $CDCl_3$) δ (ppm): 7.16-7.01 (m, 5H, Ar*H*), 6.99-6.94 (m, 2H, Ar*H*), 6.44 (dd, *J* = 8.2 Hz, *j*= 2.52, 2H, ArH), 6.26 (s 1H, Ar*H*), 5.15 (s, 1H, C*H*Ar), 3.68 (s, 3H, OCH_3), 2.34 (d, *J* = 16.28 Hz, 2H, CH_2), 2.24 (d, *J* = 16.40 Hz, 2H, CH_2), 2.19 (d, *J* = 16.16 Hz, 2H, CH_2), 2.12 (d, *J* = 16.28 Hz, 2H, CH_2), 1.02 (s, 6H, 2CH_3), 0.92 (s, 6H, 2CH_3). ^{13}C NMR (101

MHz, $CDCl_3$) δ (ppm): 27.2, 29.4, 39.4, 42.1, 50.6, 55.3, 100.5, 109.3, 109.6, 118.8, 125.8, 127.3, 128.2, 131.1, 136.3, 148.1, 150.1, 158.6, 192.9, 194.6.

10-(4-methoxyphenyl)-3,3,6,6-tetramethyl-9-phenyl-3,4,6,7,9,10 hexahydroacridine -1,8(2H,5H)-dione (4p) (Table 4, entry 16)

Light yellow solid; Yield = 87%, M.P. 209-211 °C(Lit. 213-215 °C[13]); IR (KBr): ῡ (cm^{-1}) = 3286, 2966, 1586, 1490, 1234. ^1H NMR (400 MHz, DMSO-d_6) δ (ppm): 7.16 (d, J = 5.72 Hz, 4H, ArH), 7.07-7.03 (m, 1H, ArH), 6.89-6.86 (m, 1H, ArH), 6.71-6.67 (m, 3H, ArH), 5.02 (s, 1H, CHAr), 3.74 (s, 3H, OCH_3), 2.49 (d, J = 14.08 Hz, 2H, CH_2), 2.38 (d, J = 21.88 Hz, 2H, CH_2), 2.16 (d, J = 21.16 Hz, 2H, CH_2), 1.96 (d, J = 21.52 Hz, 2H, CH_2), 1.01 (s, 6H, 2CH_3), 0.93 (s, 6H, 2CH_3). ^{13}C NMR (101 MHz, DMSO-d_6) δ (ppm): 27.1, 29.6, 31.1, 32.4, 50.5, 55.6, 113.0, 114.8, 116.7, 126.1, 127.2, 127.6, 128.5, 130.0, 149.0, 152.6, 155.7, 193.4.

10-(2-chlorophenyl)-3,3,6,6-tetramethyl-9-phenyl-3,4,6,7,9,10-hexahydroacridine-1,8(2H,5H)-dione (4q) (Table 4, entry 17)

White solid; Yield = 73%, M.P. 208-211 °C(Lit. 188-190 °C[12]); IR (KBr): ῡ (cm^{-1}) = 3416, 2956, 1640, 1364, 1222, 698. ^1H NMR (400 MHz, $CDCl_3$) δ (ppm): 7.65-7.00 (m, 9H, ArH), 5.16 (s, 1H, CHAr), 2.07 (d, J = 8.36 Hz, 2H, CH_2), 1.94 (d, J = 17.44 Hz, 2H, CH_2), 1.54 (d, J = 17.24 Hz, 2H, CH_2), 1.22 (d, J = 25.92 Hz, 2H, CH_2) 0.85 (s, 6H, 2CH_3), 0.76 (s, 6H, 2CH_3). ^{13}C NMR (101 MHz, $CDCl_3$) δ (ppm): 30.1, 32.2, 38.7, 41.7, 50.1, 114.8, 125.8, 127.7, 128.2, 128.7, 128.8, 130.8, 130.9, 130.9, 131.6, 146.1, 148.4, 195.7.

10-(3-chlorophenyl)-3,3,6,6-tetramethyl-9-phenyl-3,4,6,7,9,10-hexahydroacridine-1,8(2H,5H)-dione (4q) (Table 4, entry 18)

White solid; Yield = 77%, M.P. 262-264 °C(Lit. 177-180 °C[12]); IR (KBr): ῡ (cm^{-1}) = 3278, 3192, 3070, 1600, 1480, 1284, 696. ^1H NMR (300 MHz, DMSO-d_6) δ (ppm): 7.25-7.07 (m, 5H, ArH), 6.95-6.88 (m, 4H, ArH), 5.05 (s, 1H, CHAr), 2.53 (d, J = 10.32 Hz, 2H, CH_2), 2.40 (d, J = 16.83 Hz, 2H, CH_2), 2.19 (d, J = 15.99 Hz, 2H, CH_2), 2.00 (d, J = 16.11 Hz, 2H, CH_2) ,1.03 (s, 6H, 2CH_3), 0.95 (s, 6H, 2CH_3). ^{13}C NMR (75MHz, DMSO-d_6) δ (ppm): 27.1, 29.5, 30.5, 32.5, 50.5, 107.1, 115.0, 122.8, 125.2, 126.3, 127.3, 128.6, 131.4, 131.7, 138.0, 148.6, 152.0, 193.6.

10-(4-chlorophenyl)-3,3,6,6-tetramethyl-9-phenyl-3,4,6,7,9,10-hexahydroacridine-1,8(2H,5H)-dione (4q) (Table 4, entry 19)

White solid; Yield = 78%, M.P. 295-297 °C(Lit. 301-303 °C[13]); IR (KBr): ū (cm^{-1}) = 3416, 3058, 2956, 1636, 1364, 1222, 698. ^1H NMR (400 MHz, DMSO-d_6) δ (ppm): 7.48 (d, *J* = 8.24 Hz, 2H, Ar*H*), 7.34 (d, *J* = 7.52 Hz, 2H, Ar*H*), 7.17-7.12 (m, 4H, Ar*H*), 7.04 (t, *J* = 7.30 Hz, 1H, Ar*H*), 5.20 (s, 1H, CHAr), 2.13 (d, *J* = 16.28 Hz, 2H, CH_2), 2.06 (d, *J* = 16.60 Hz, 2H, CH_2), 1.98 (d, *J* = 17.36 Hz, 2H, CH_2),1.74 (d, *J* = 18.16 Hz, 2H, CH_2), 0.89 (s, 6H, 2CH_3), 0.74 (s, 6H, 2CH_3). ^{13}C NMR (101MHz, DMSO-d_6) δ (ppm): 26.8, 29.7, 32.4, 32.6, 50.1, 114.9, 118.1, 122.1, 126.0, 127.8, 128.1, 135.4, 137.6, 145.9, 149.1, 195.7.

3,3,6,6-tetramethyl-9-phenyl-10-propyl-3,4,6,7,9,10-hexahydroacridine-1,8(2H,5H)-dione (4t) (Table 4, entry 20)

Light yellow solid; Yield = 94%, M.P. 180-182 °C (Lit. new); IR (KBr): ū(cm^{-1}) = 2958, 1640, 1384, 1212. ^1H NMR (400 MHz, $CDCl_3$) δ (ppm): 7.27 (d, *J* = 6.08 Hz, 2H, Ar*H*), 7.18 (t, *J* = 15.00 Hz, 2H, Ar*H*), 7.06 (t, *J* = 7.24 Hz, 1H, Ar*H*), 5.28 (s, 1H, C*H*Ar), 3.62 (t, *J* = 7.68 Hz, 2H, CH_2N), 2.54 (d, *J* = 16.60 Hz, 2H, CH_2), 2.41 (d, *J* = 16.72 Hz, 2H, CH_2), 2.31 (d, *J* = 7.24 Hz, 2H, CH_2), 2.23 (d, *J* = 8.24 Hz, 2H, CH_2), 1.68 (sext, *J* = 8.00 Hz, 2H, C$H_2$$CH_3$), 1.11 (s, 6H), 1.01 (s, 6H, 2CH_3), 0.90 (t, *J* = 3.06 Hz, 3H, 2CH_3), 2.41 (d, *J* = 16.72 Hz, 2H, CH_3). ^{13}C NMR (101MHz, $CDCl_3$) δ (ppm): 11.05, 24.70, 29.36, 31.84, 32.55, 40.47, 46.44, 49.96, 115.41, 125.81, 127.62, 127.95, 145.74, 150.16, 195.63.

3,3,6,6,10-pentamethyl-9-phenyl-3,4,6,7,9,10-hexahydroacridine-1,8(2H,5H)-dione (4u) (Table 4, entry 21)

Light yellow solid; Yield = 96%, M.P. 232-233 °C (Lit. 200-202 °C[14]); IR (KBr): ū(cm^{-1}) = 3470, 29.31, 16.17, 1325. ^1H NMR (400 MHz, $CDCl_3$) δ (ppm): 7.44 (t, *J* = 8.34 Hz, 4H, Ar*H*), 7.33-7.36 (m, 1H, Ar*H*), 4.89 (s, 1H, C*H*Ar), 3.72 (s, 1H, CH_3N), 2.19 (d, *J* = 8.20 Hz, 2H, CH_2), 2.14 (d, *J* = 5.28 Hz, 2H, CH_2), 2.09 (d, *J* = 5.76 Hz, 2H, CH_2), 2.05 (d, *J* = 5.28 Hz, 2H, CH_2), 1.19 (s, 6H, 2CH_3), 1.07 (s, 6H, 2CH_3). ^{13}C NMR (101MHz, $CDCl_3$) δ (ppm): 26.81, 28.99, 34.99, 38.00, 50.62, 113.26, 128.14, 128.47, 128.61, 146.84, 153.70, 188.98.

9-phenyl-3,4,6,7,9,10-hexahydroacridine-1,8(2H,5H)-dione (4v) (Table 4, entry 22)

Light yellow solid; Yield = 92%, M.P. 307-308 °C (Lit. 310 °C [3]); IR (KBr): ū(cm^{-1}) = 3449, 2929, 1620, 1372. ^1H NMR (400 MHz, $CDCl_3$) δ (ppm): 9.33 (s, 1H, N-*H*), 7.33 (d, *J* = 7.80 Hz, 4H, Ar*H*), 7.13-7.05 (m, 1H, Ar*H*), 4.96 (s, 1H, C*H*Ar), 3.26 (t, *J* = 7.32 Hz, 4H CH_2CO), 2.98 (t, *J* = 6.12 Hz, 4H CH_2), 1.83 (quin, 4H, C$H_2$$CH_2$CO). ^{13}C NMR (101MHz, $CDCl_3$) δ (ppm): 21.05, 28.39, 30.05, 35.43, 112.19, 131.17, 132.78, 134.19, 142.67, 150.61, 195.72.

1,1'-(2,6-dimethyl-4-phenyl-1,4-dihydropyridine-3,5-diyl)bis(ethan-1-one) (4w) (Table 4, entry 23)

Yellow solid; Yield = 94%, M.P. 186-187 °C (Lit. 184-186 °C [15]); IR (KBr): ῡ(cm^{-1}) = 3407, 2915, 1621, 1397. ^{1}H NMR (400 MHz, DMSO-d_6) δ (ppm): 8.98 (s, 1H, N-*H*), 7.33 (d, *J* = 19.88 Hz, 4H, Ar*H*), 7.17-7.10 (m, 1H, Ar*H*), 4.88 (s, 1H, C*H*Ar), 2.38 (s, 6H, 2CH_3CO), 2.16 (s, 6H, 2CH_3). ^{13}C NMR (101MHz, DMSO-d_6) δ (ppm): 20.06, 27.21, 33.96, 112.85, 128.09, 128.78, 129.61, 143.48, 147.28, 198.59.

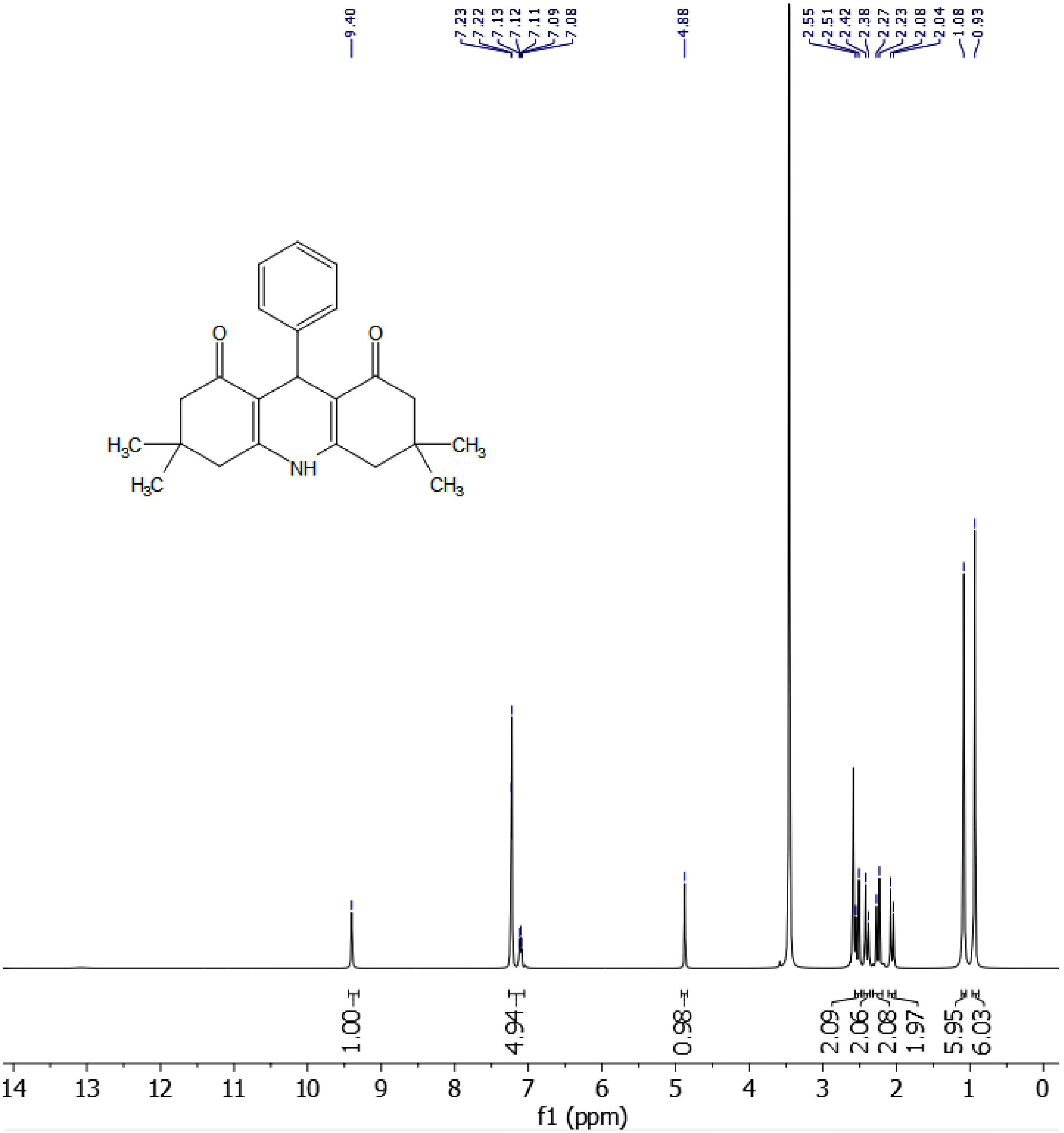

Figure S1: The ^{1}H NMR spectrum (400 MHz) of 3,3,6,6-tetramethyl-9-phenyl-3,4,6,7,9,10-hexahydroacridine-1,8(2*H*,5*H*)-dione in DMSO-d_6

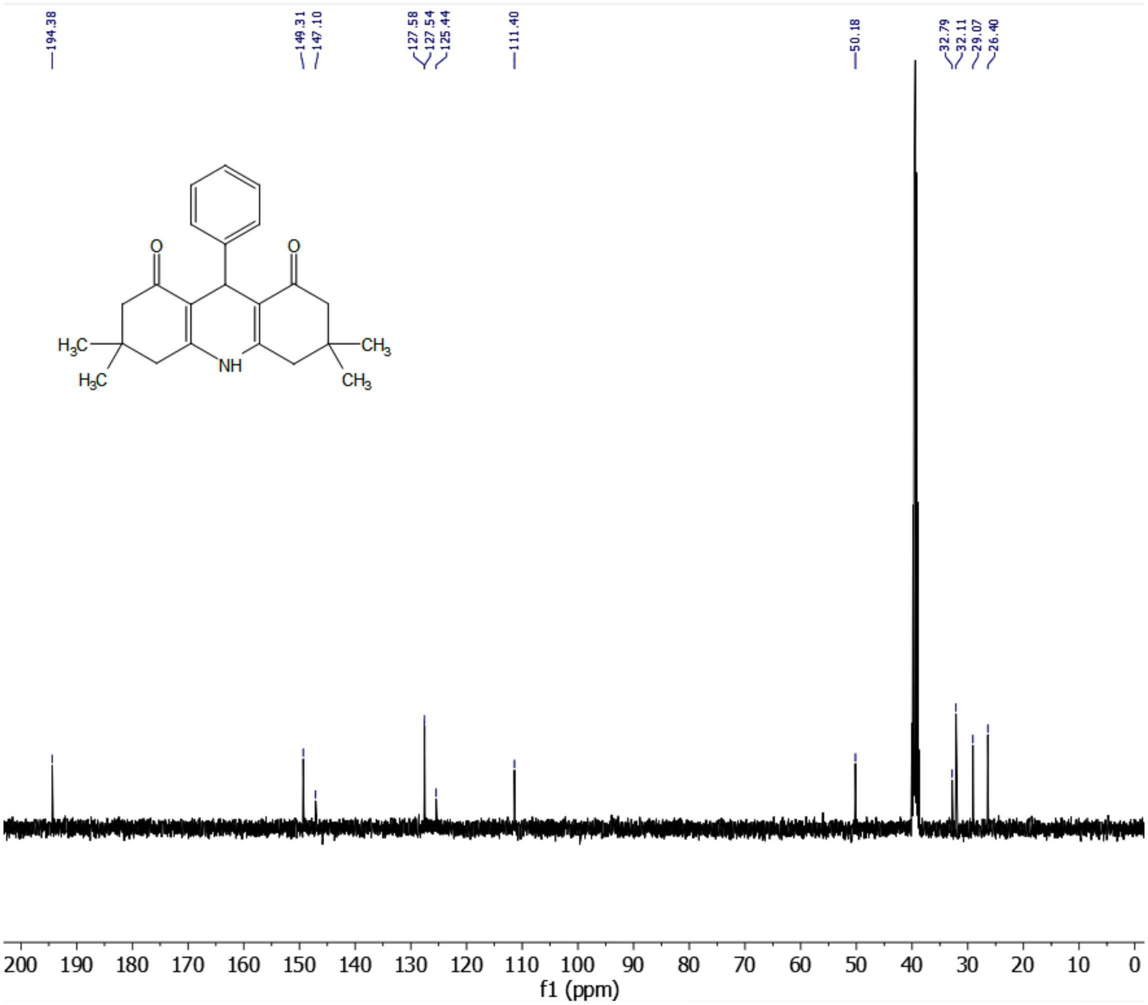

Figure S2: The ^{13}C NMR spectrum (101 MHz) of spectrum of 3,3,6,6-tetramethyl-9-phenyl-3,4,6,7,9,10-hexahydroacridine-1,8(2*H*,5*H*)-dione in DMSO-d_6

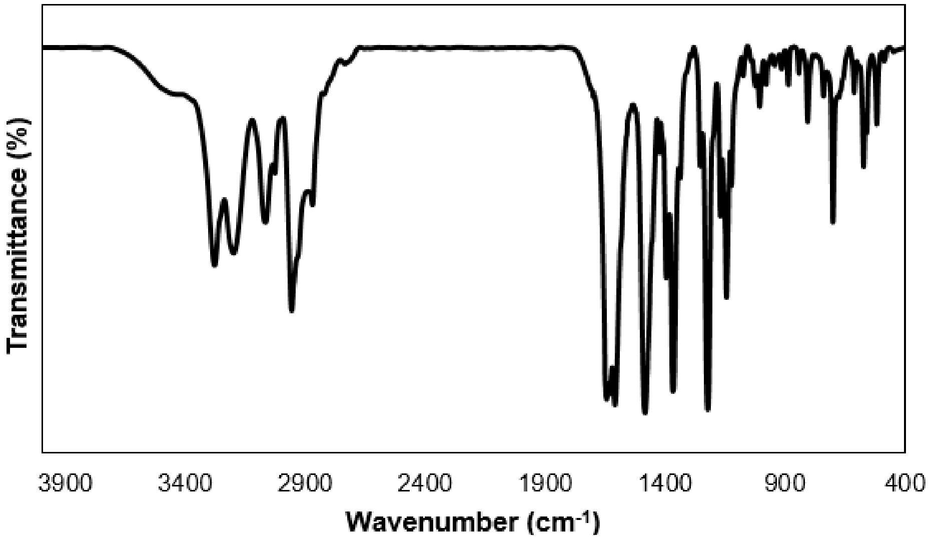

Figure S3: The FT-IR spectrum of 3,3,6,6-tetramethyl-9-phenyl-3,4,6,7,9,10-hexahydroacridine-1,8(2*H*,5*H*)-dione in KBr

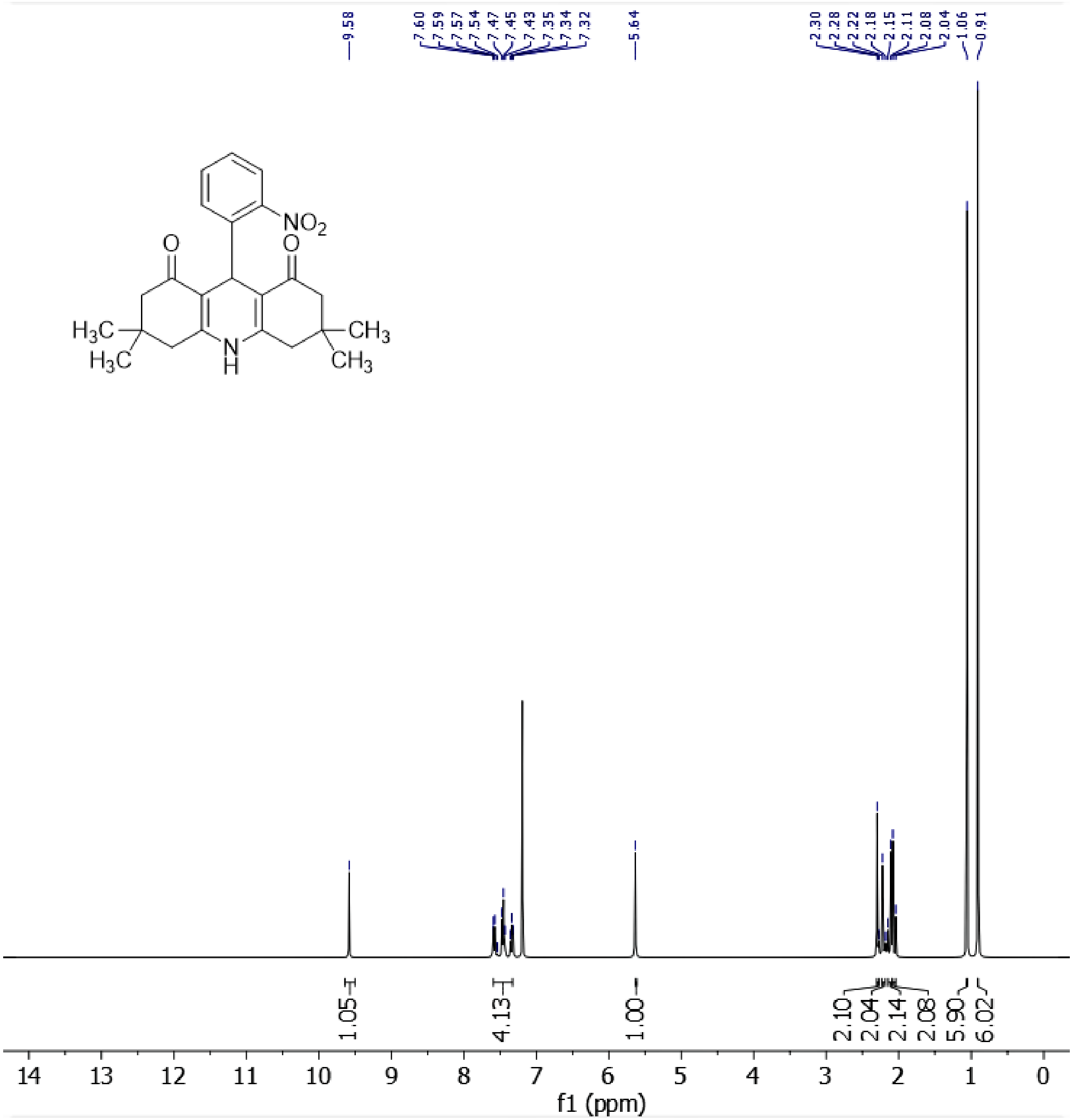

Figure S4: The ^{1}H NMR spectrum (400 MHz) of 3,3,6,6-tetramethyl-9-(2-nitrophenyl)-3,4,6,7,9,10-hexahydroacridine-1,8(2*H*,5*H*)-dione in $CDCl_3$

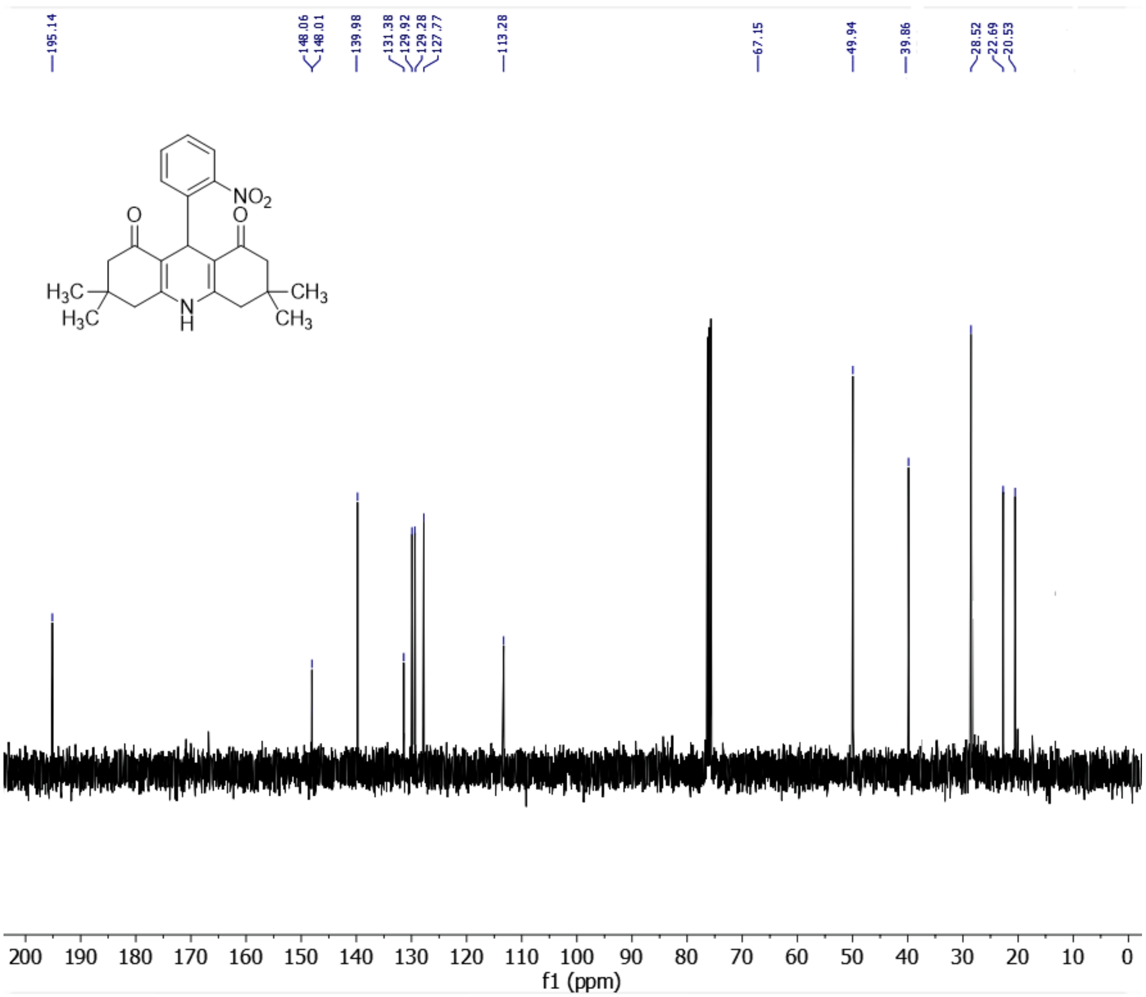

Figure S5: The ^{13}C NMR spectrum (101 MHz) of spectrum of 3,3,6,6-tetramethyl-9-(2-nitrophenyl)-3,4,6,7,9,10-hexahydroacridine-1,8(2*H*,5*H*)-dione in $CDCl_3$

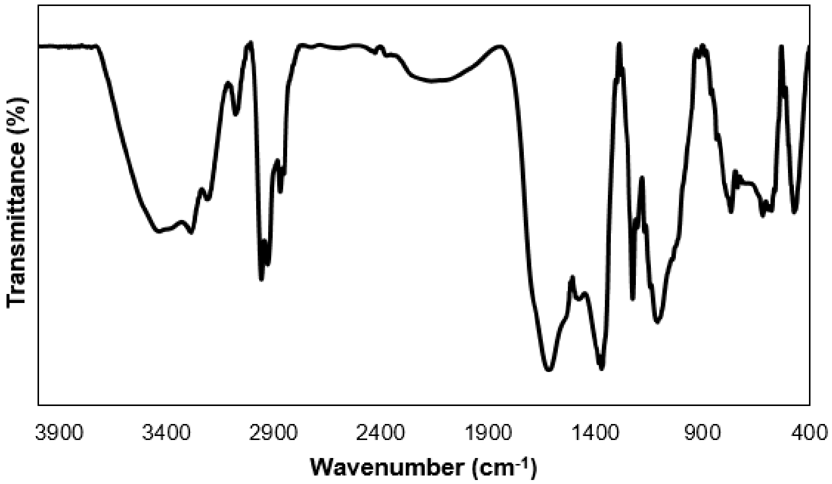

Figure S6: The FT-IR spectrum of 3,3,6,6-tetramethyl-9-(2-nitrophenyl)-3,4,6,7,9,10-hexahydroacridine-1,8(2*H*,5*H*)-dione in KBr

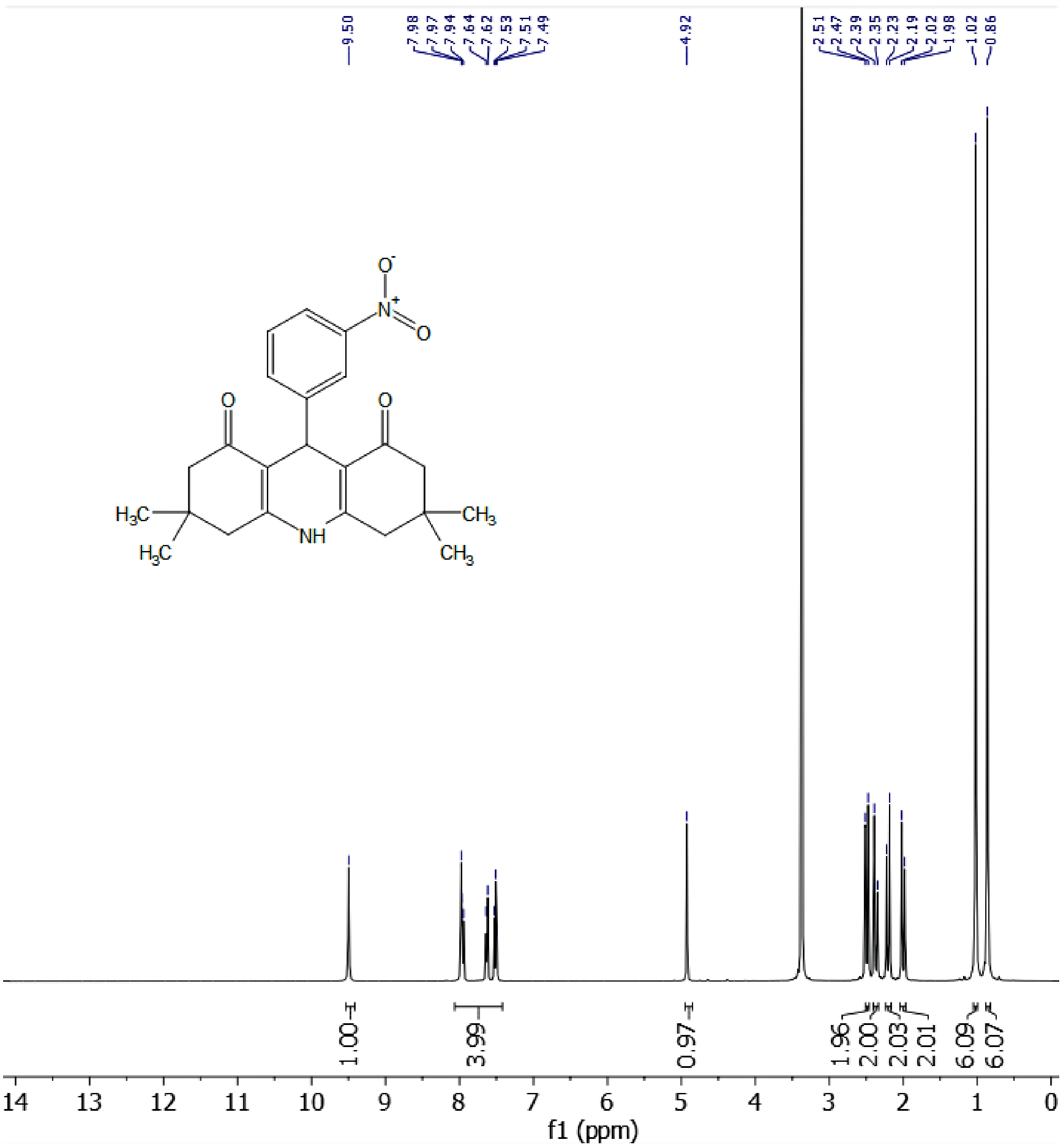

Figure S7: The ^{1}H NMR spectrum (400 MHz) of 3,3,6,6-tetramethyl-9-(3-nitrophenyl)-3,4,6,7,9,10-hexahydroacridine-1,8(2*H*,5*H*)-dione in DMSO-d_6

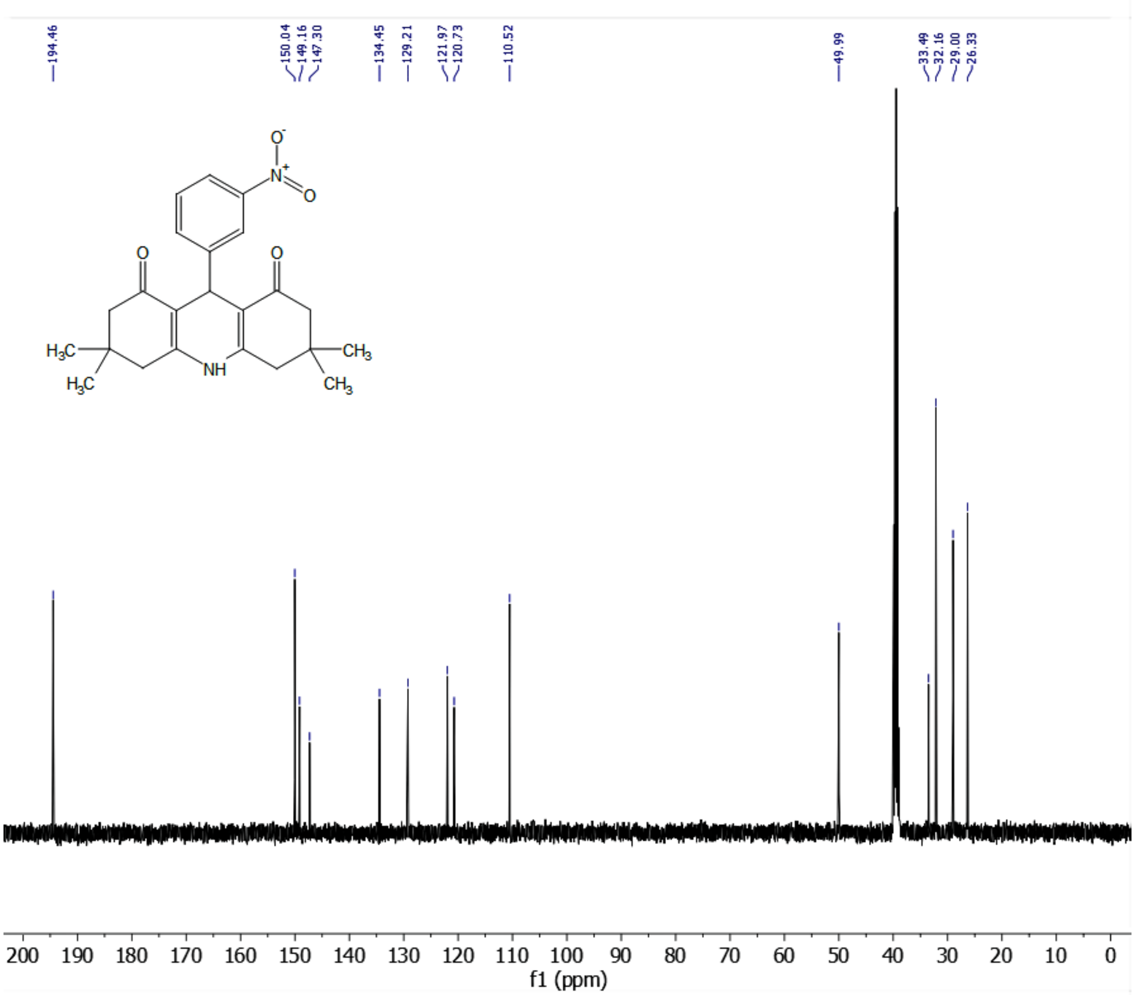

Figure S8: The ^{13}C NMR spectrum (101 MHz) of 3,3,6,6-tetramethyl-9-(3-nitrophenyl)-3,4,6,7,9,10-hexahydroacridine-1,8(2*H*,5*H*)-dione in DMSO-d_6

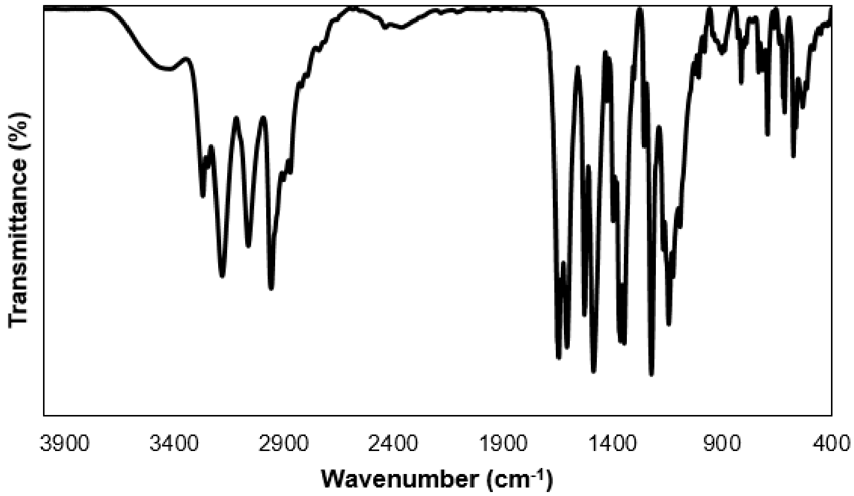

Figure S9: The FT-IR spectrum of 3,3,6,6-tetramethyl-9-(3-nitrophenyl)-3,4,6,7,9,10-hexahydroacridine-1,8(2*H*,5*H*)-dione in KBr

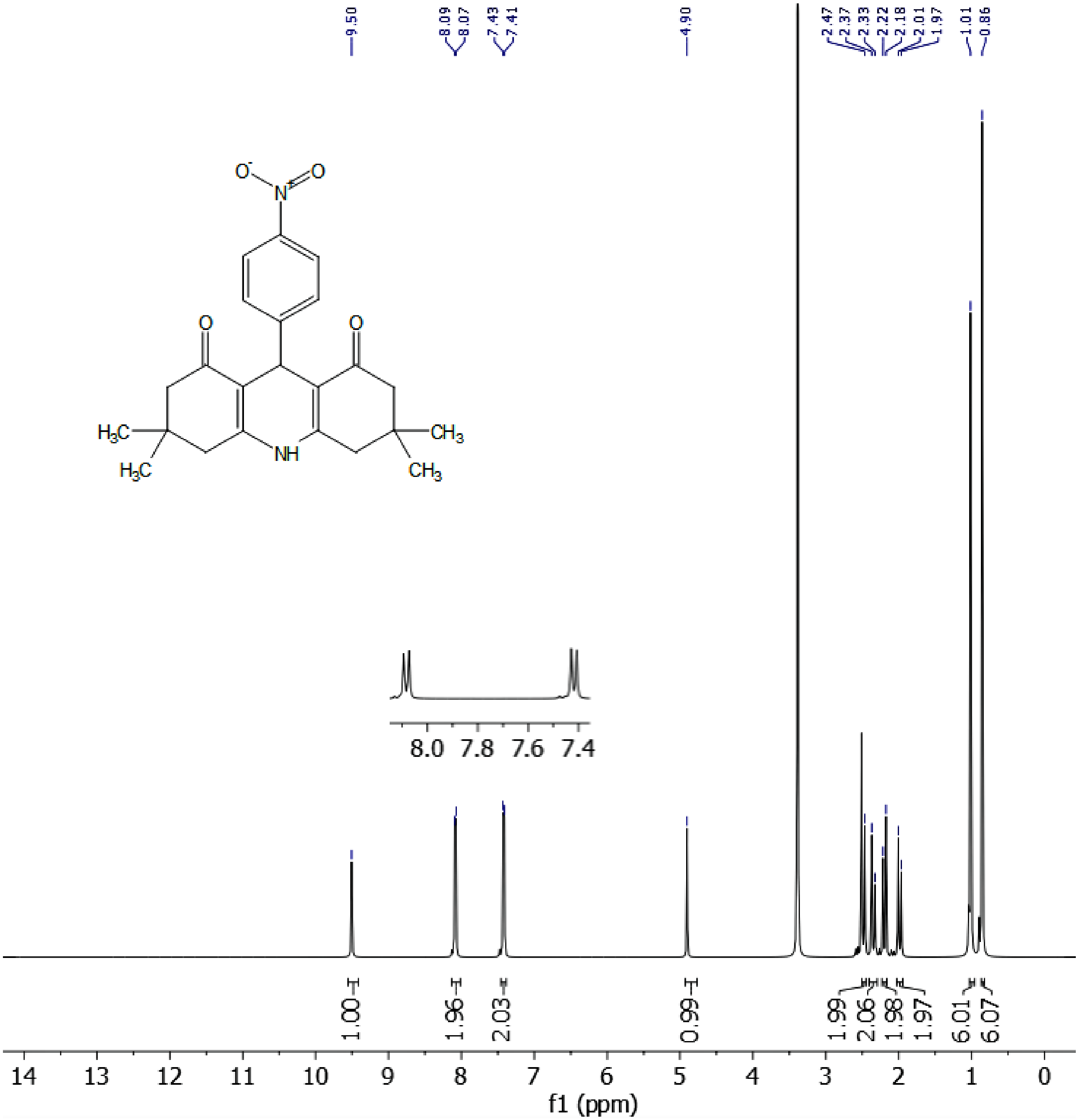

Figure S10: The ^{1}H NMR spectrum (400 MHz) of 3,3,6,6-tetramethyl-9-(4-nitrophenyl)-3,4,6,7,9,10-hexahydroacridine-1,8(2*H*,5*H*)-dione in DMSO-d_6

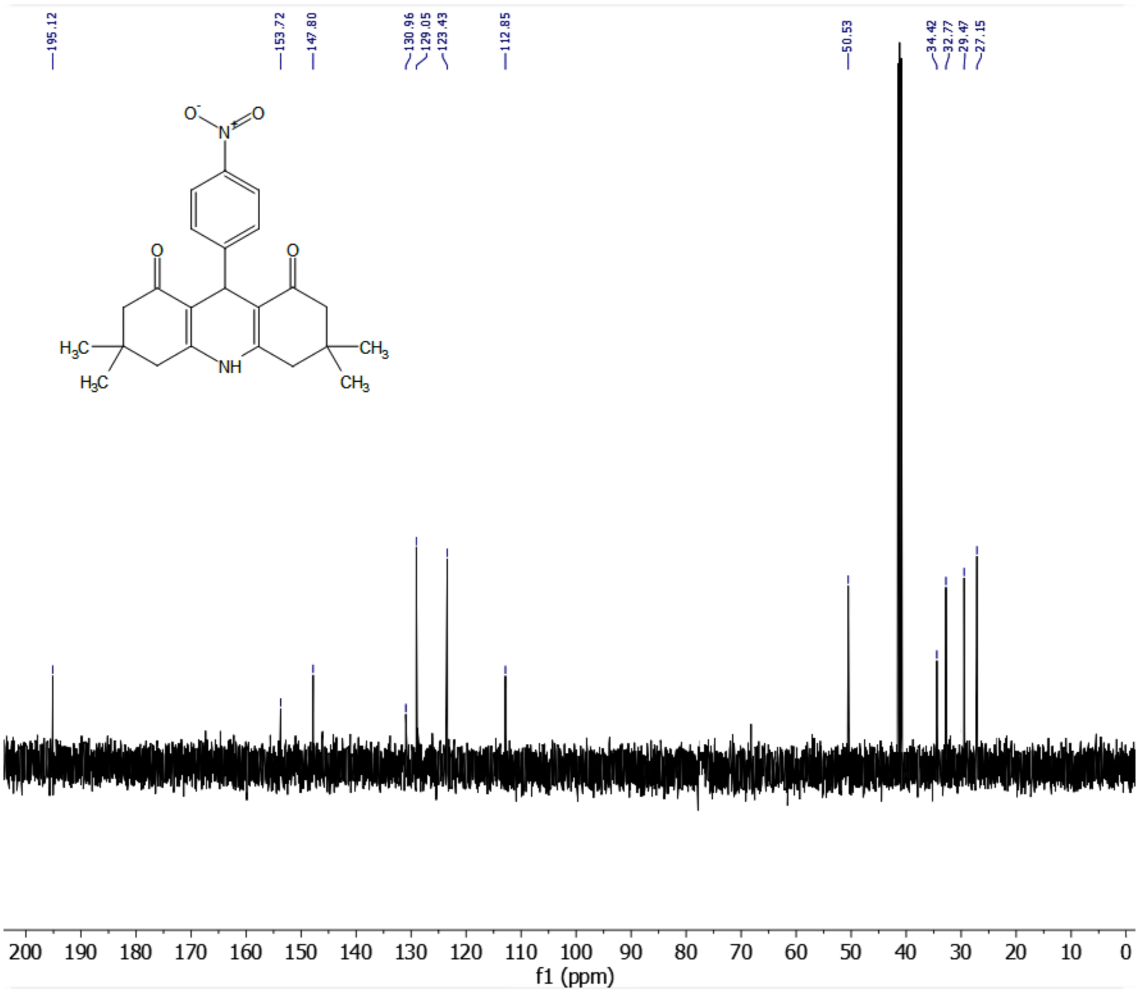

Figure S11: The ^{13}C NMR spectrum (101 MHz) of 3,3,6,6-tetramethyl-9-(4-nitrophenyl)-3,4,6,7,9,10-hexahydroacridine-1,8(2*H*,5*H*)-dione in DMSO-d_6

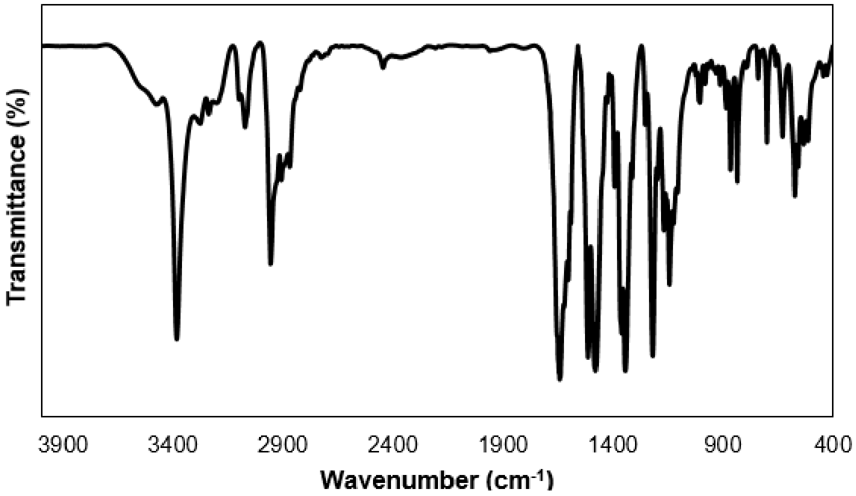

Figure S12: The FT-IR spectrum of 3,3,6,6-tetramethyl-9-(4-nitrophenyl)-3,4,6,7,9,10-hexahydroacridine-1,8(2*H*,5*H*)-dione in KBr

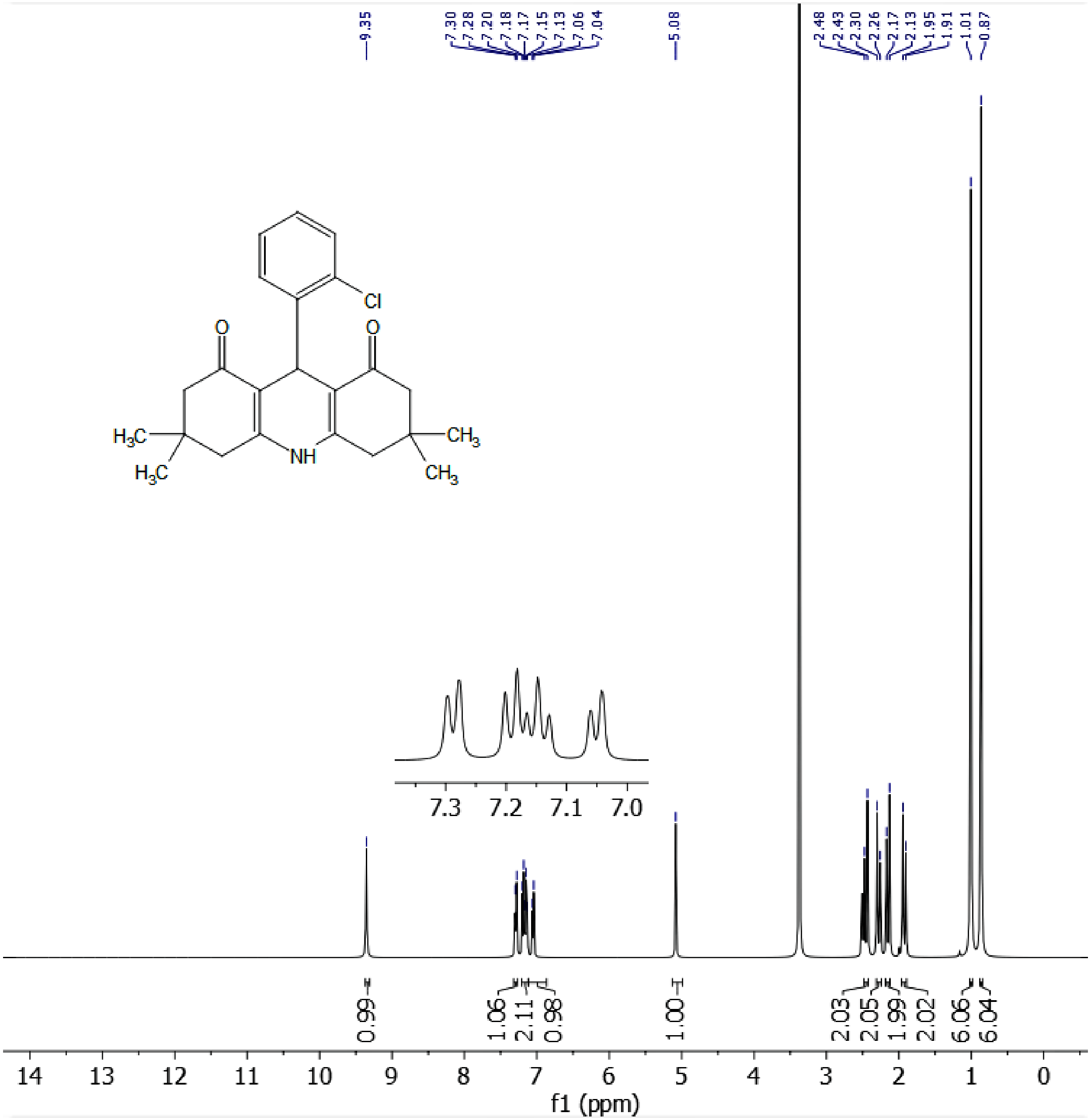

Figure S13: The ^{1}H NMR spectrum of (400 MHz) 9-(2-chlorophenyl)-3,3,6,6-tetramethyl-3,4,6,7,9,10-hexahydroacridine-1,8(2*H*,5*H*)-dione in DMSO-d_6

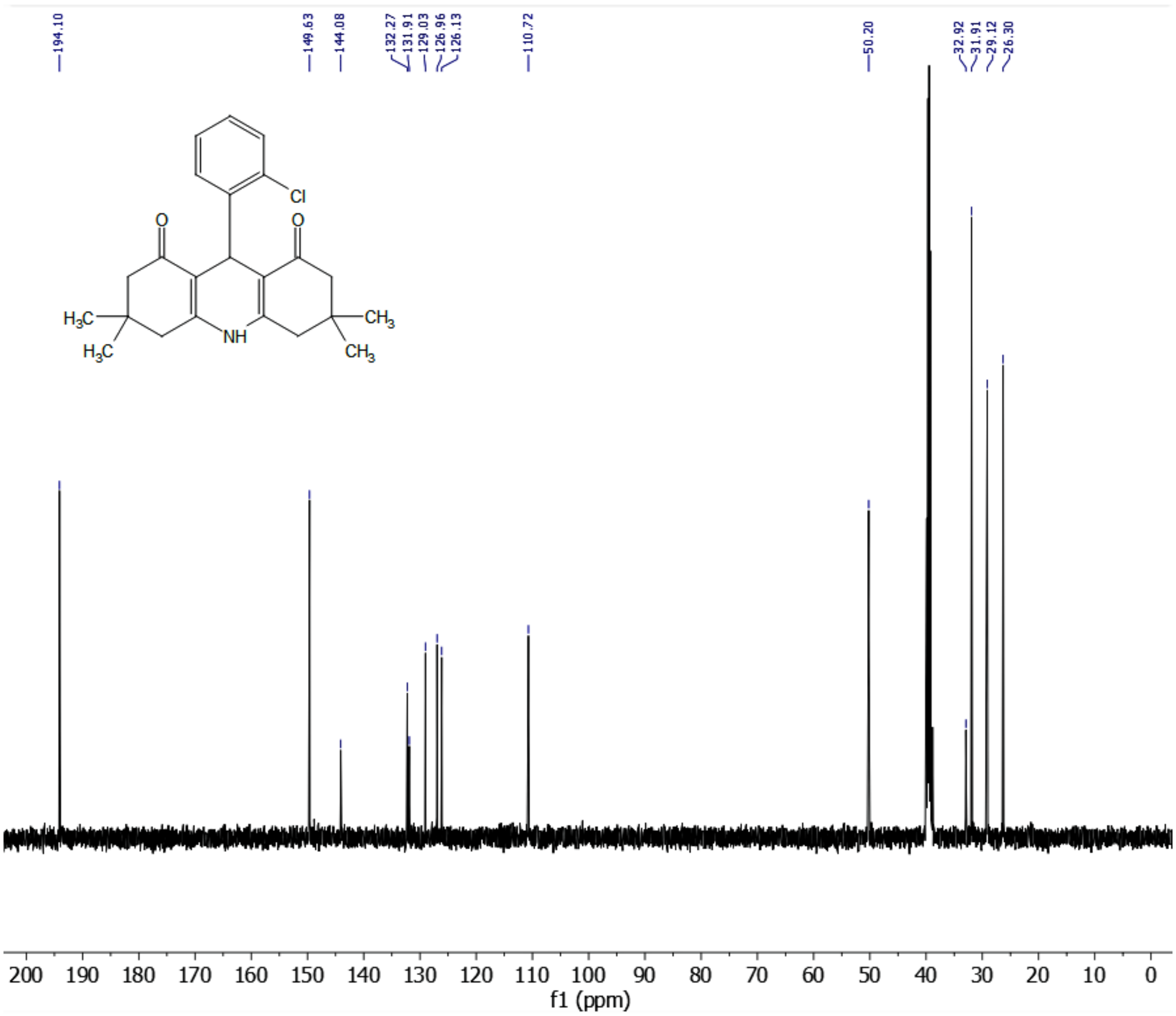

Figure S14: The ^{13}C NMR spectrum (101 MHz) of 9-(2-chlorophenyl)-3,3,6,6-tetramethyl-3,4,6,7,9,10-hexahydroacridine-1,8(2*H*,5*H*)-dione in DMSO-d_6

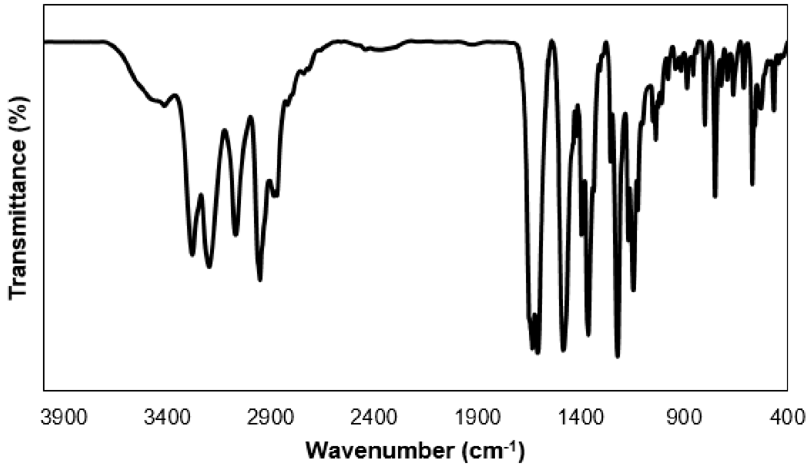

Figure S15: The FT-IR spectrum of 9-(2-chlorophenyl)-3,3,6,6-tetramethyl-3,4,6,7,9,10-hexahydroacridine-1,8(2*H*,5*H*)-dione in KBr

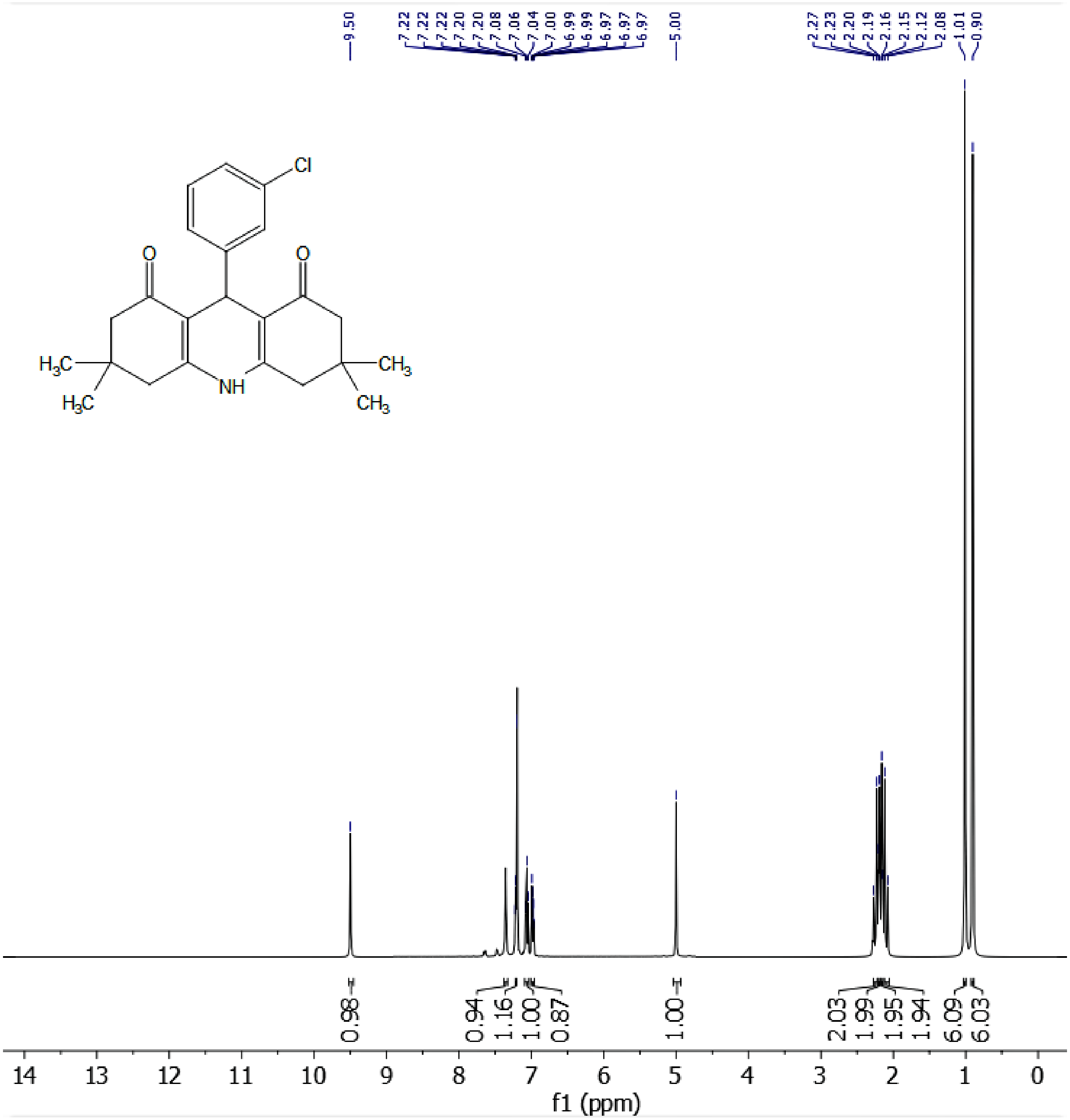

Figure S16: The ^{1}H NMR spectrum (400 MHz) of 9-(3-chlorophenyl)-3,3,6,6-tetramethyl-3,4,6,7,9,10-hexahydroacridine-1,8(2*H*,5*H*)-dione in $CDCl_3$

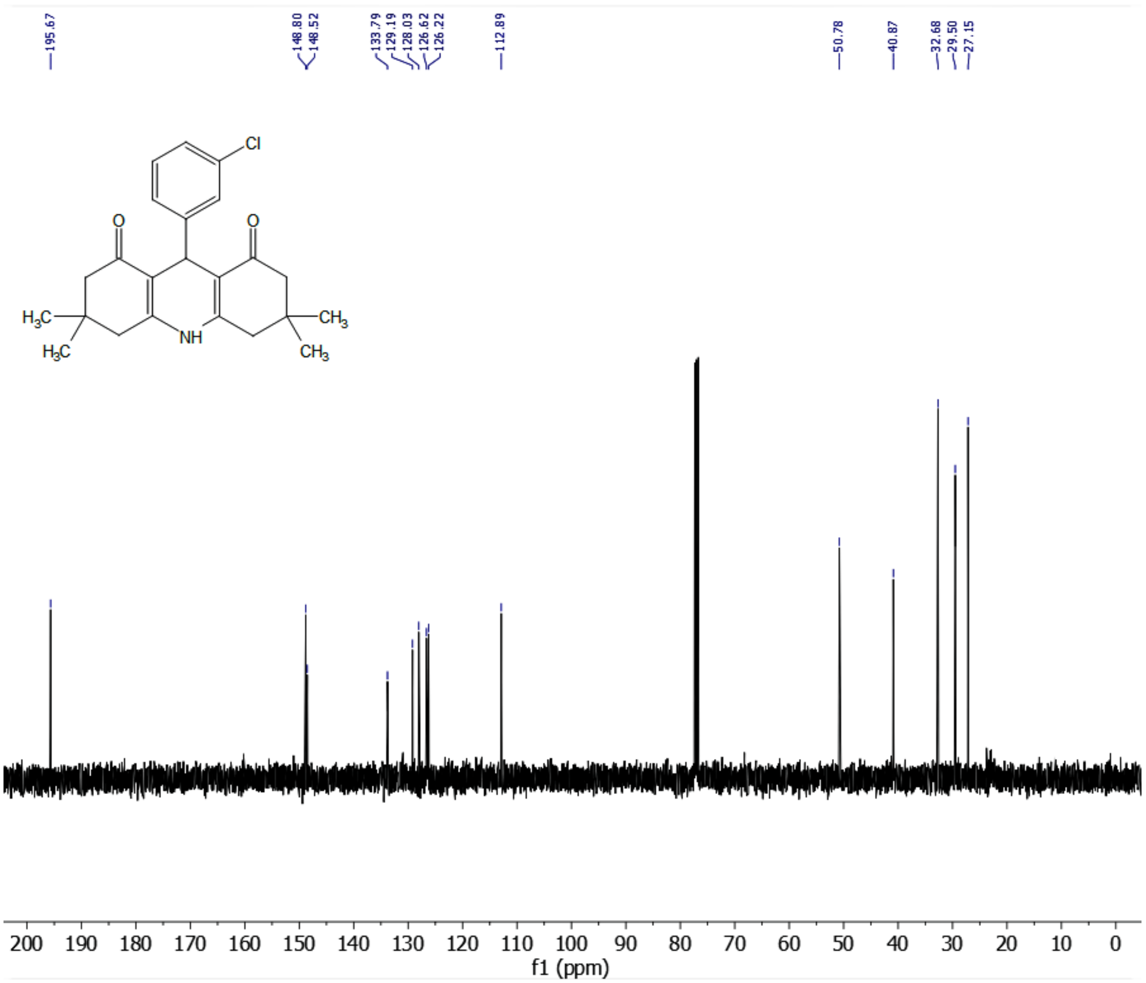

Figure S17: The ^{13}C NMR spectrum (101 MHz) of 9-(3-chlorophenyl)-3,3,6,6-tetramethyl-3,4,6,7,9,10-hexahydroacridine-1,8(2*H*,5*H*)-dione (4f) in $CDCl_3$

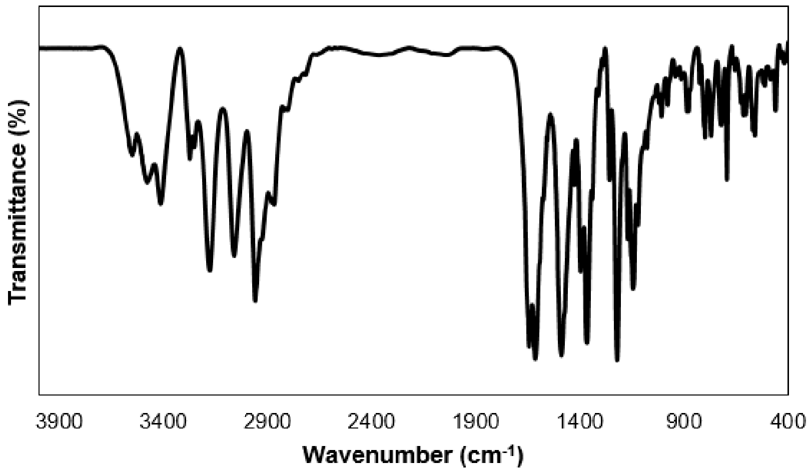

Figure S18: The FT-IR spectrum of 9-(3-chlorophenyl)-3,3,6,6-tetramethyl-3,4,6,7,9,10-hexahydroacridine-1,8(2*H*,5*H*)-dione in KBr

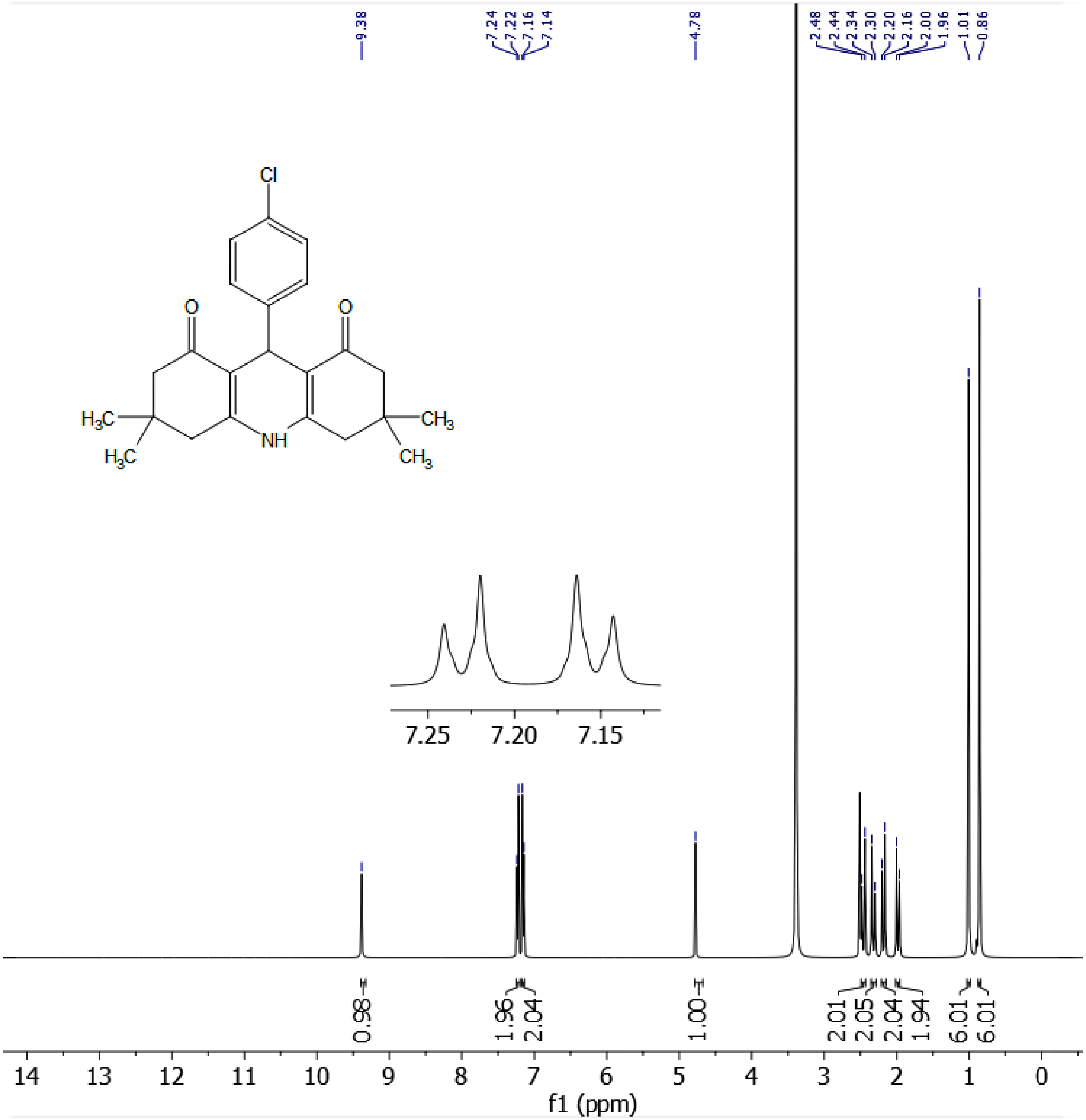

Figure S19: The ^{1}H NMR spectrum (400 MHz) of 9-(4-chlorophenyl)-3,3,6,6-tetramethyl-3,4,6,7,9,10-hexahydroacridine-1,8(2*H*,5*H*)-dione in DMSO-d_6

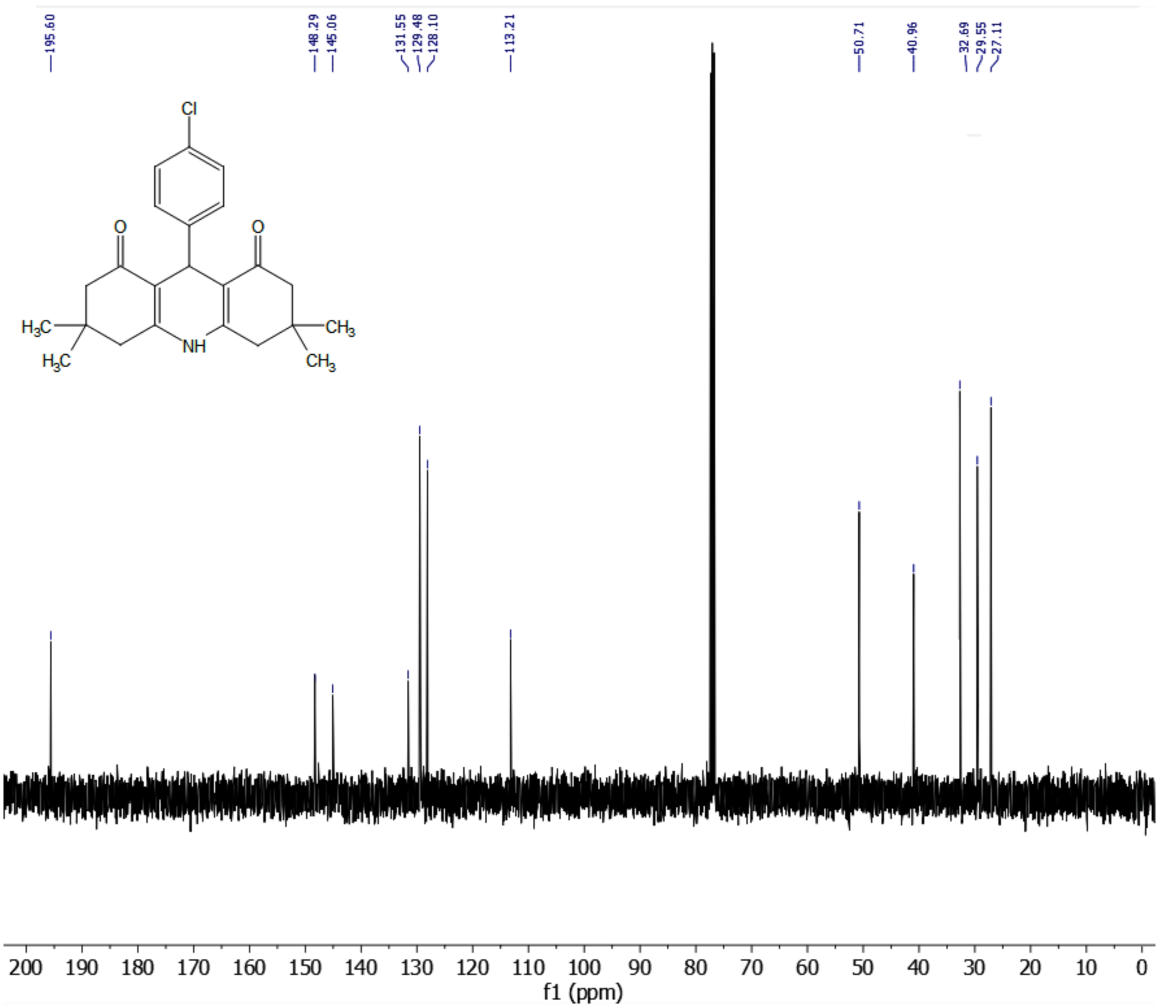

Figure S20: The ^{13}C NMR spectrum (101 MHz) of 9-(4-chlorophenyl)-3,3,6,6-tetramethyl-3,4,6,7,9,10-hexahydroacridine-1,8(2*H*,5*H*)-dione in DMSO-d_6

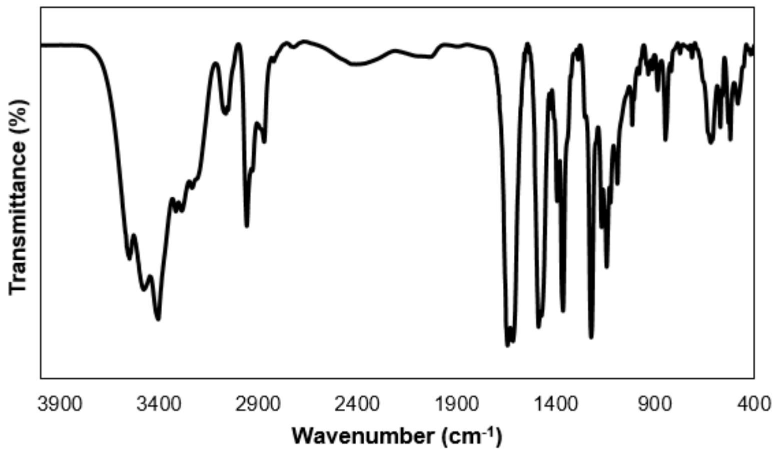

Figure S21: The FT-IR spectrum of 9-(4-chlorophenyl)-3,3,6,6-tetramethyl-3,4,6,7,9,10-hexahydroacridine-1,8(2*H*,5*H*)-dione in KBr

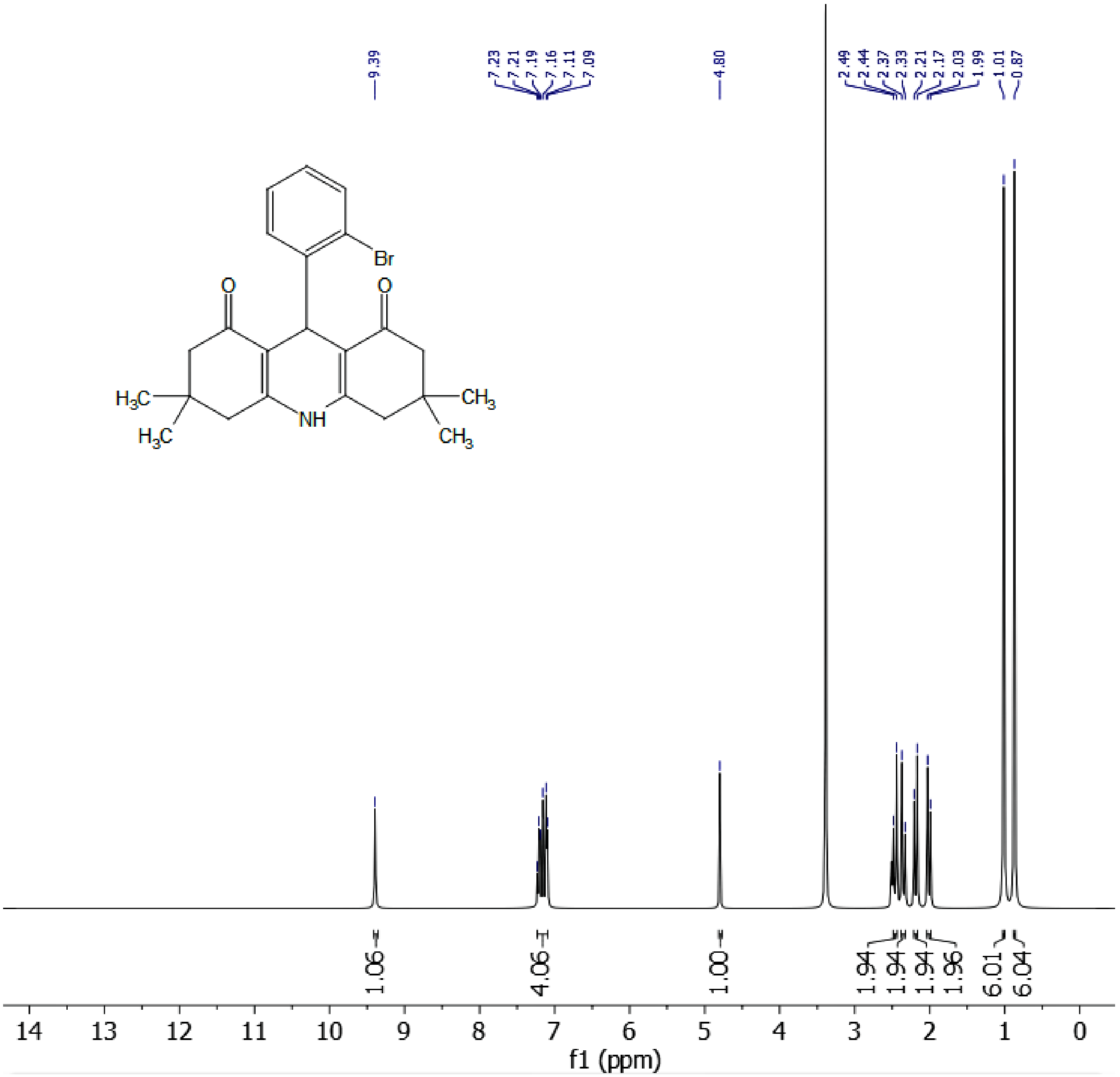

Figure S22: The ^{1}H NMR spectrum (400 MHz) of 9-(2-bromophenyl)-3,3,6,6-tetramethyl-3,4,6,7,9,10-hexahydroacridine-1,8(2*H*,5*H*)-dione in DMSO-d_6

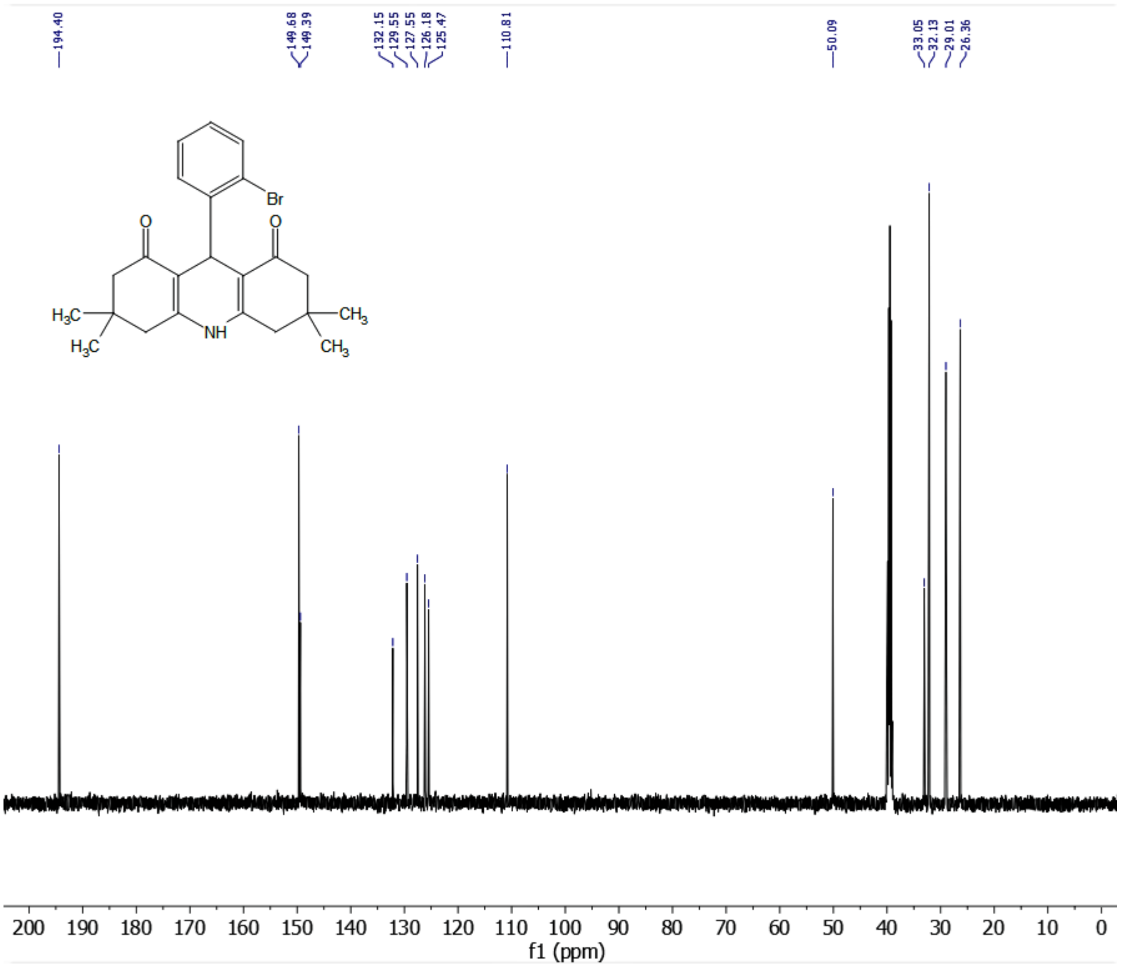

Figure S23: The ^{13}C NMR spectrum (101 MHz) of 9-(2-bromophenyl)-3,3,6,6-tetramethyl-3,4,6,7,9,10-hexahydroacridine-1,8(2*H*,5*H*)-dione in DMSO-d_6

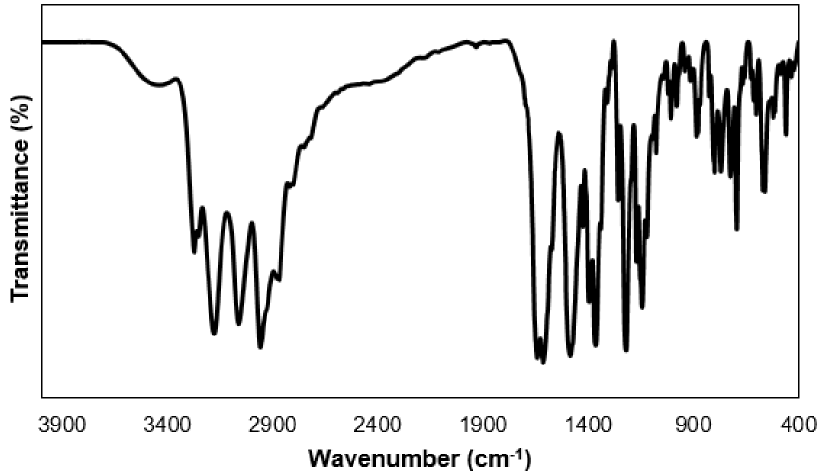

Figure S24: The FT-IR spectrum of 9-(2-bromophenyl)-3,3,6,6-tetramethyl-3,4,6,7,9,10-hexahydroacridine-1,8(2*H*,5*H*)-dione in KBr

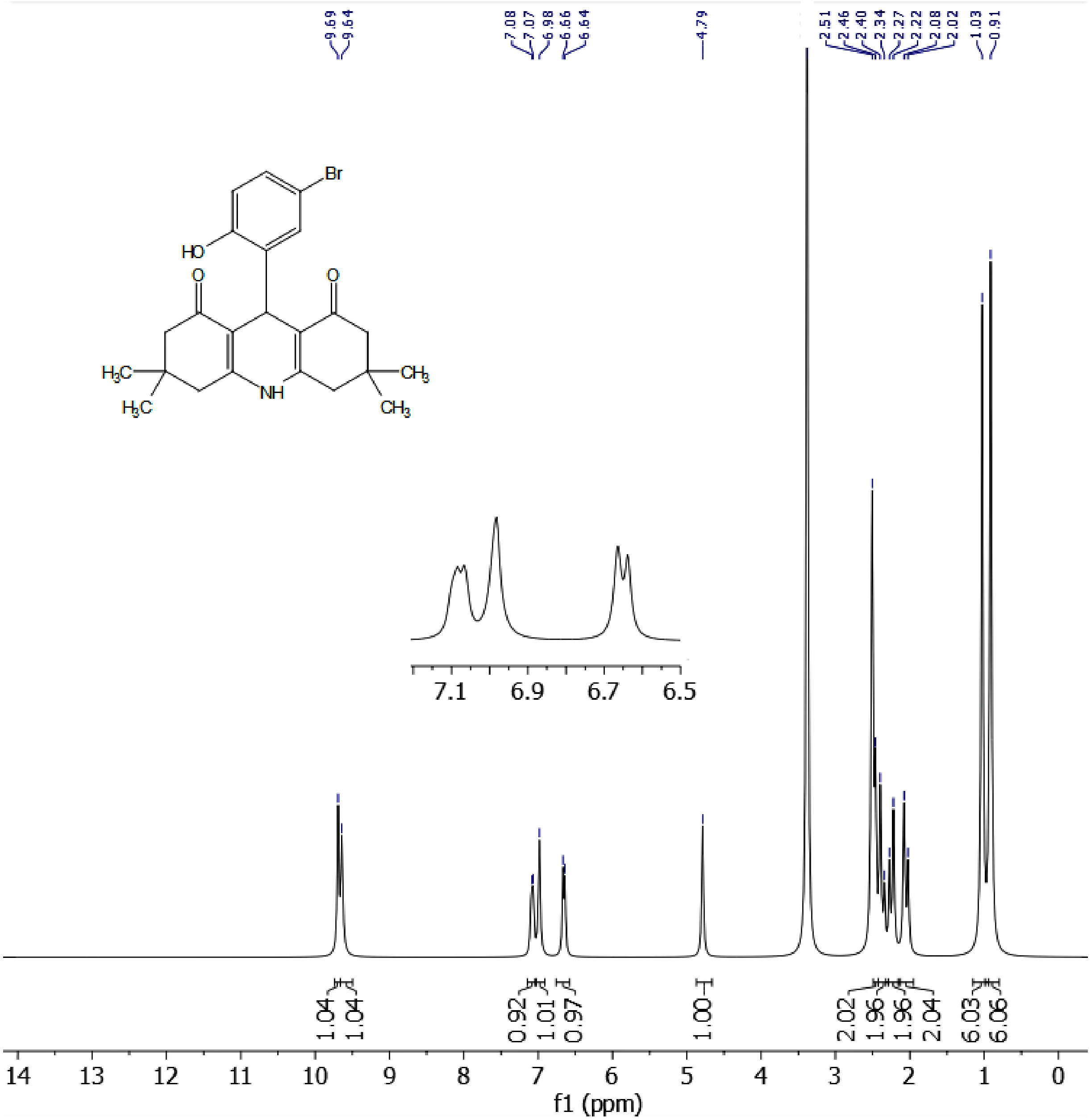

Figure S25: The ^{1}H NMR spectrum (300 MHz)of 9-(5-bromo-2-hydroxyphenyl)-3,3,6,6-tetramethyl-3,4,6,7,9,10-hexahydroacridine-1,8(2*H*,5*H*)-dione in DMSO-d_6

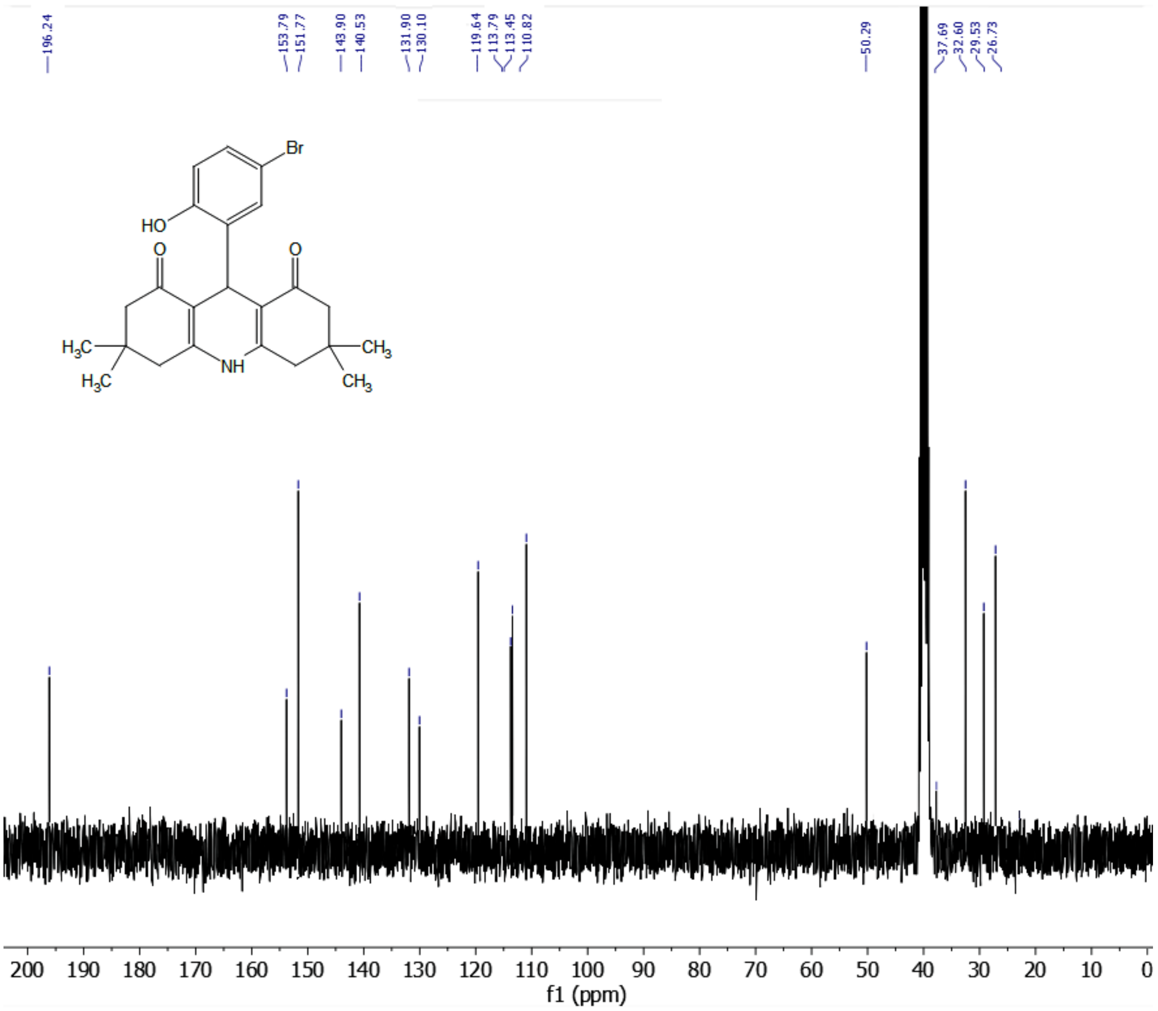

Figure S26: The ^{13}C NMR spectrum (76 MHz) of 9-(5-bromo-2-hydroxyphenyl)-3,3,6,6-tetramethyl-3,4,6,7,9,10-hexahydroacridine-1,8(2*H*,5*H*)-dione in DMSO-d_6

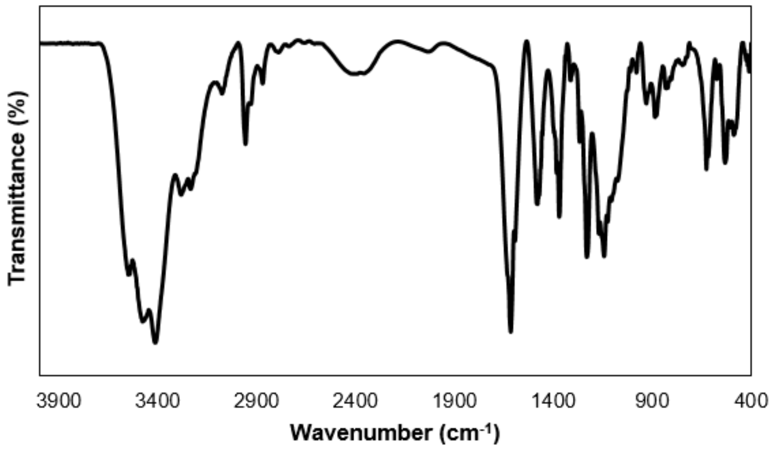

Figure S27: The FT-IR spectrum of 9-(5-bromo-2-hydroxyphenyl)-3,3,6,6-tetramethyl-3,4,6,7,9,10-hexahydroacridine-1,8(2*H*,5*H*)-dione in KBr

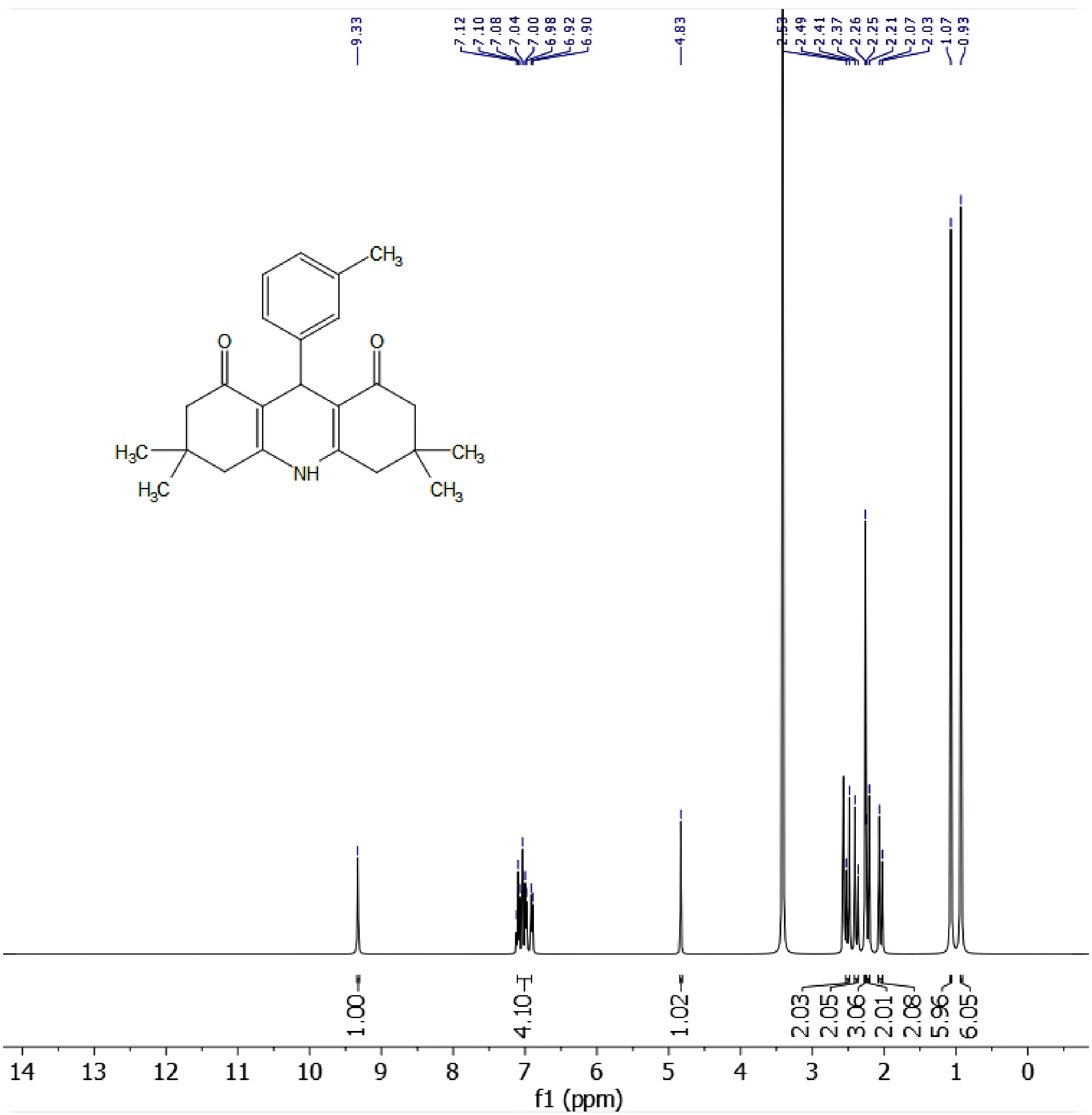

Figure S28: The ^{1}H NMR spectrum (400 MHz) of 3,3,6,6-tetramethyl-9-(*m*-tolyl)-3,4,6,7,9,10-hexahydroacridine-1,8(2*H*,5*H*)-dione in DMSO-d_6

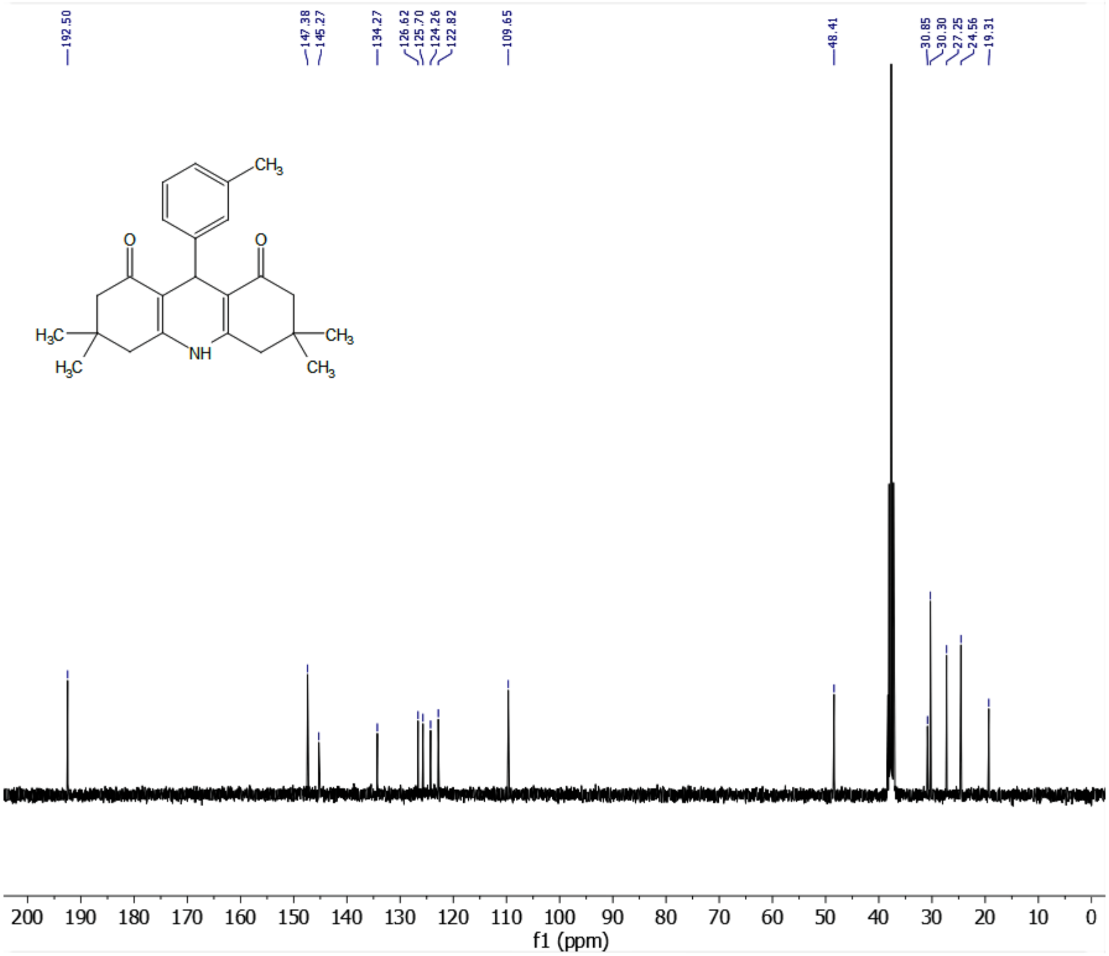

Figure S29: The ^{13}C NMR spectrum (101 MHz) of 3,3,6,6-tetramethyl-9-(*m*-tolyl)-3,4,6,7,9,10-hexahydroacridine-1,8(2*H*,5*H*)-dione in DMSO-d_6

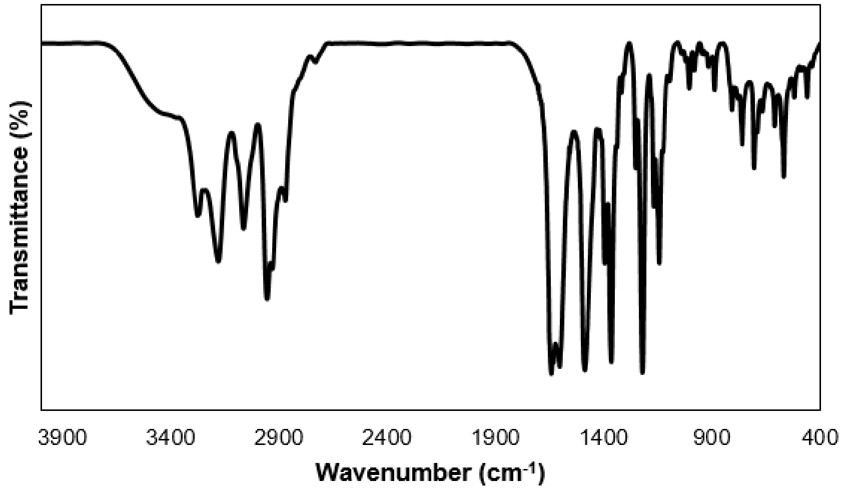

Figure S30: The FT-IR spectrum of 3,3,6,6-tetramethyl-9-(*m*-tolyl)-3,4,6,7,9,10-hexahydroacridine-1,8(2*H*,5*H*)-dione in KBr

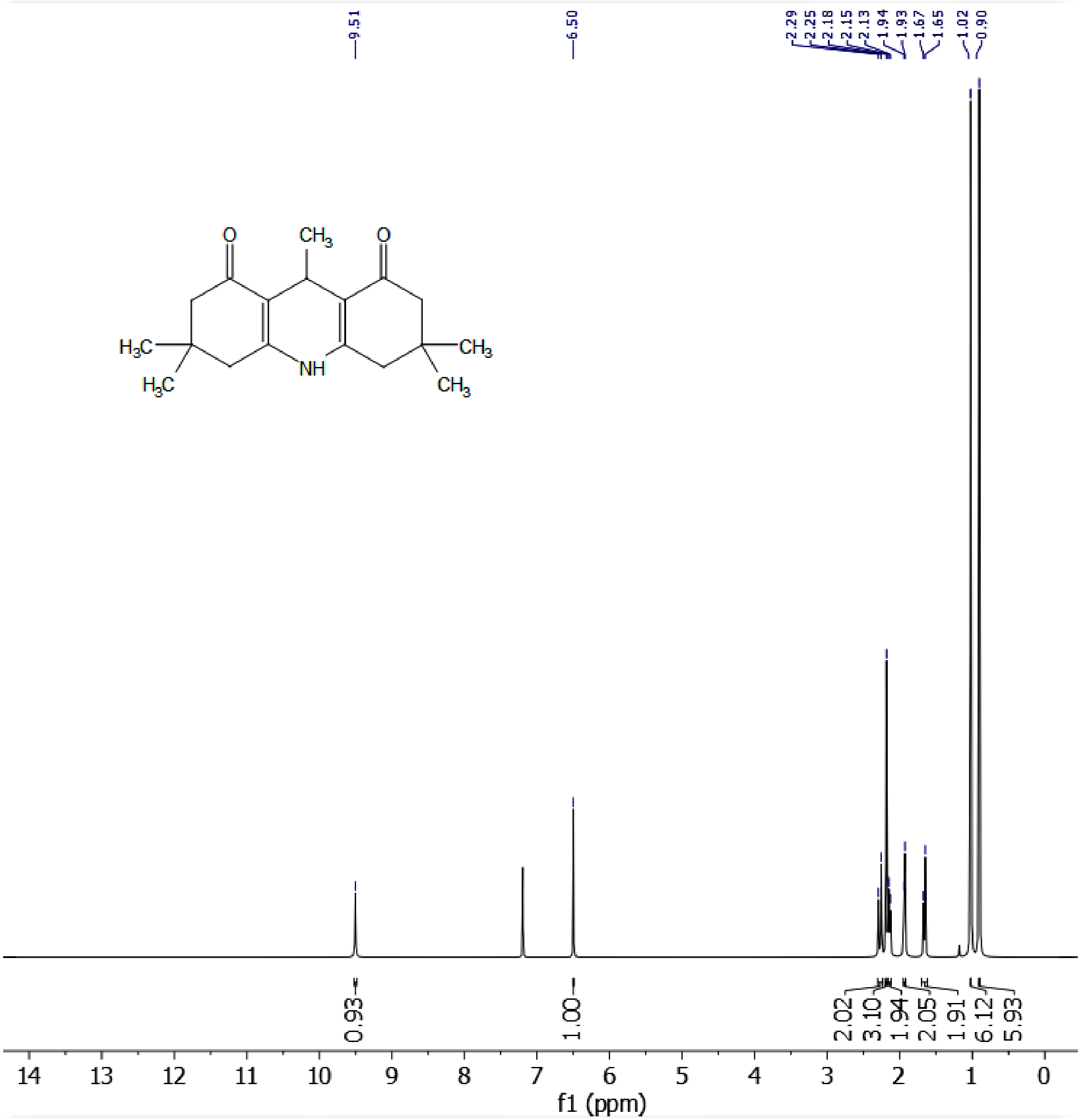

Figure S31: The ^{1}H NMR spectrum (400 MHz) of 3,3,6,6,9-pentamethyl-3,4,6,7,9,10-hexahydroacridine-1,8(2*H*,5*H*)-dione in $CDCl_3$

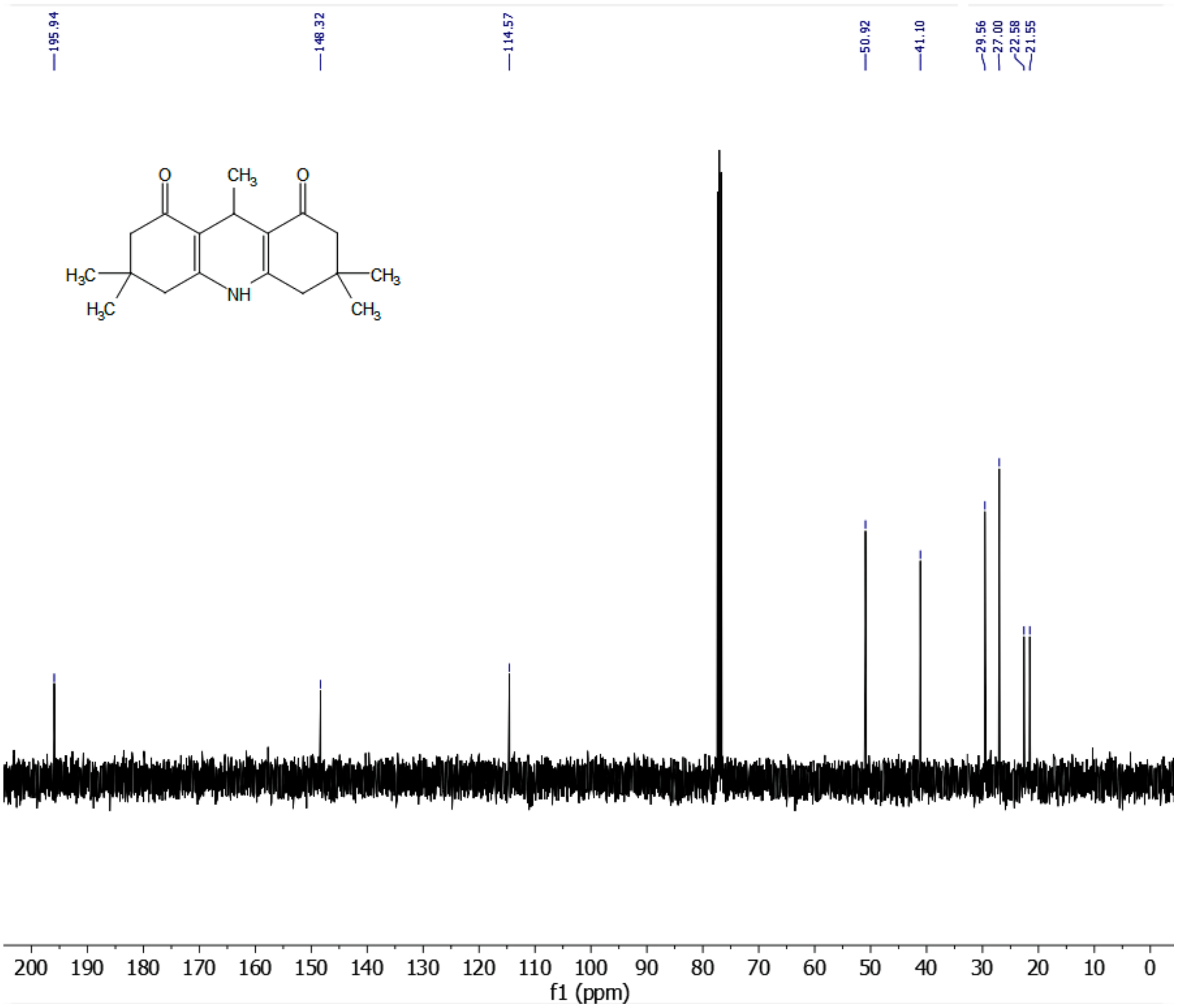

Figure S32: The ^{13}C NMR spectrum (101 MHz) of 3,3,6,6,9-pentamethyl-3,4,6,7,9,10-hexahydroacridine-1,8(2*H*,5*H*)-dione in $CDCl_3$

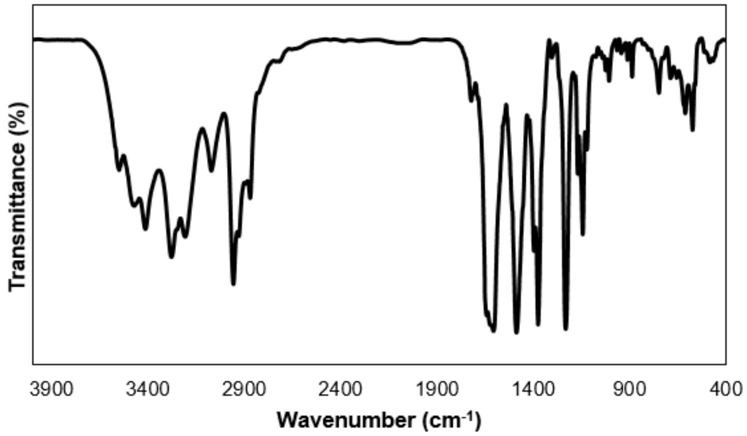

Figure S33: The FT-IR spectrum of 3,3,6,6,9-pentamethyl-3,4,6,7,9,10-hexahydroacridine-1,8(2*H*,5*H*)-dione in KBr

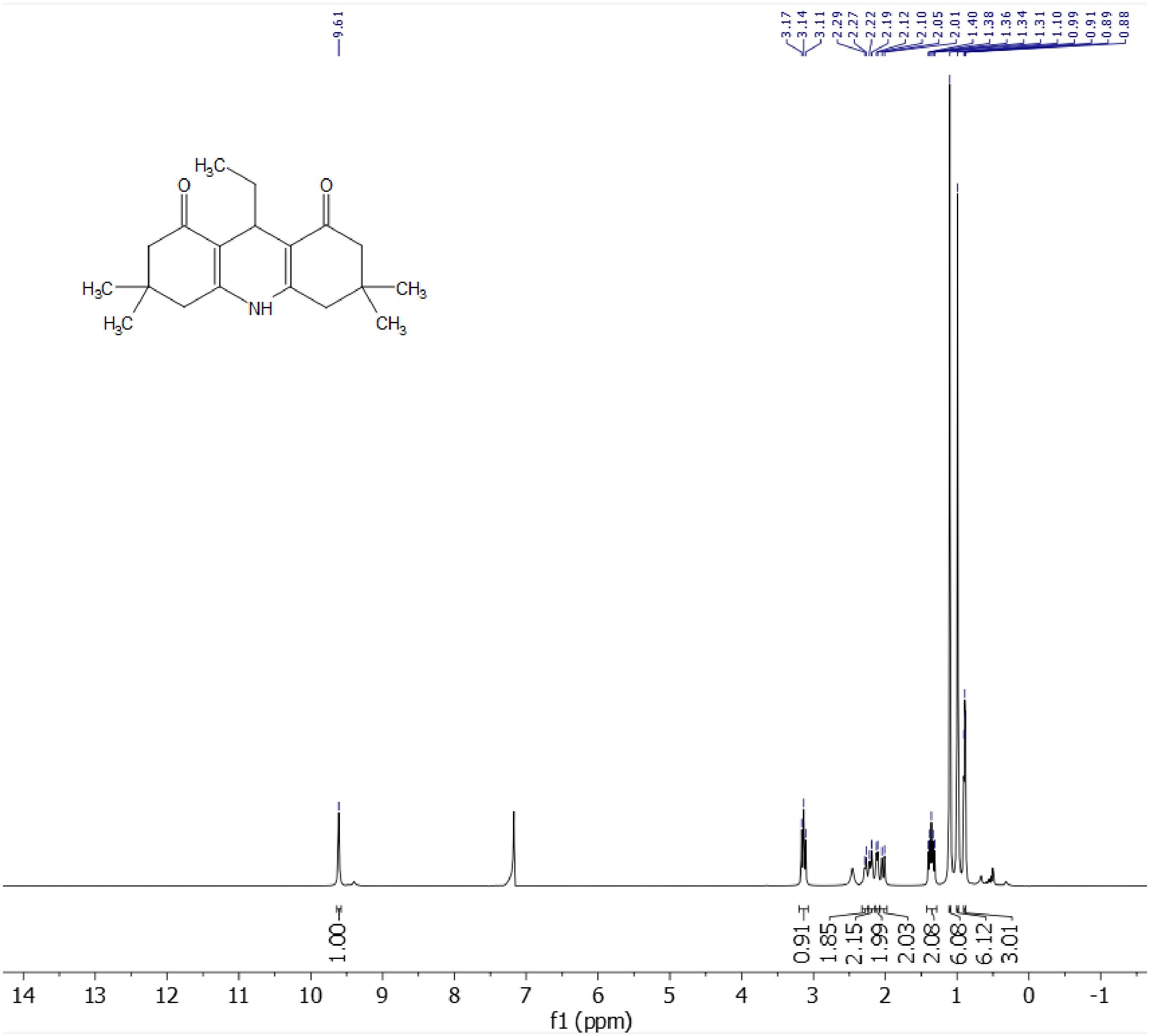

Figure S34: The ^{1}H NMR spectrum (400 MHz) of 9-ethyl-3,3,6,6-tetramethyl-3,4,6,7,9,10-hexahydroacridine-1,8(2H,5H)-dione in $CDCl_3$

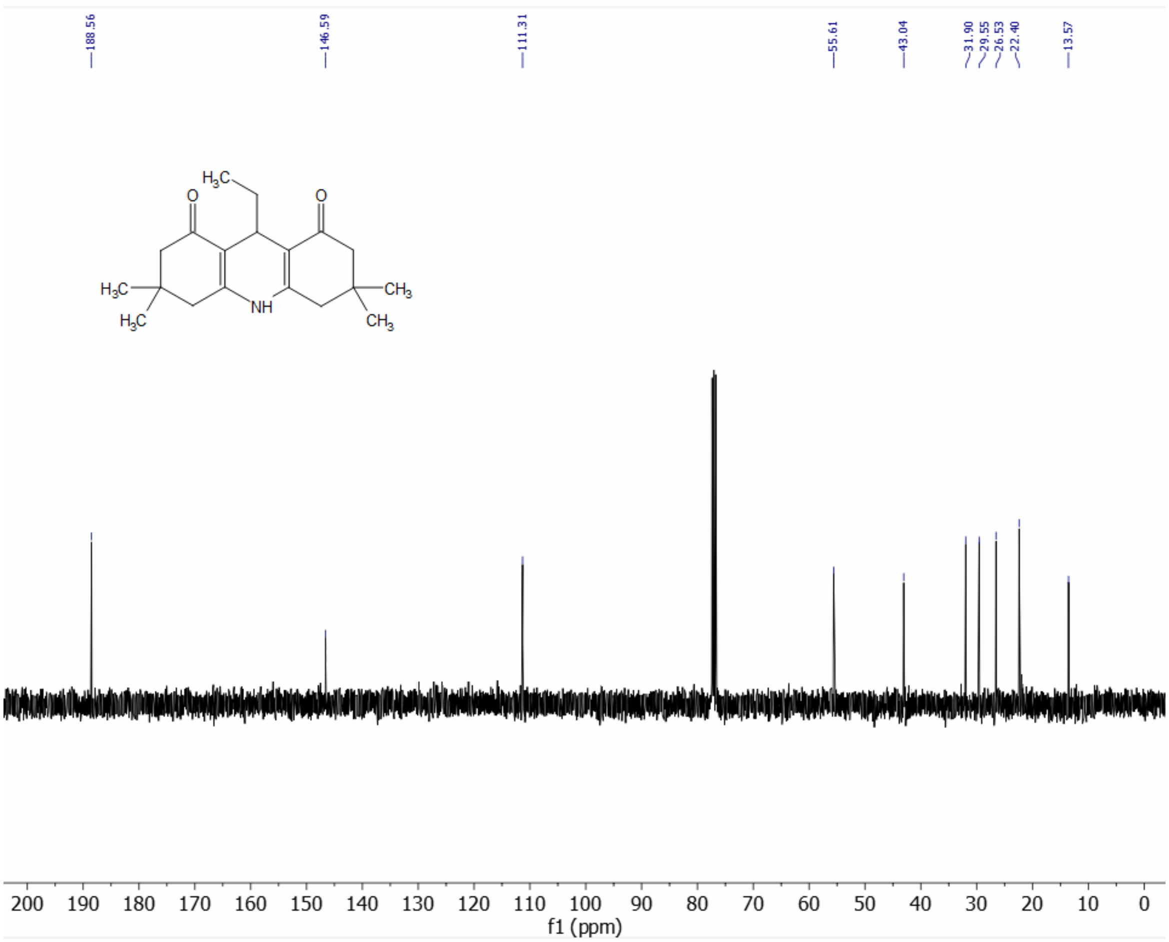

Figure S35: The ^{13}C NMR spectrum (101 MHz) of 9-ethyl-3,3,6,6-tetramethyl-3,4,6,7,9,10-hexahydroacridine-1,8(2H,5H)-dione $CDCl_3$

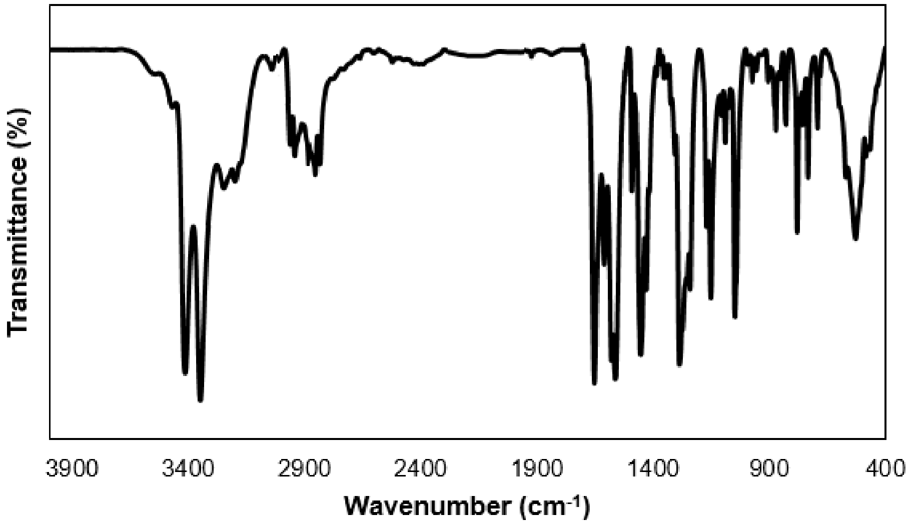

Figure S36: The FT-IR spectrum of 9-ethyl-3,3,6,6-tetramethyl-3,4,6,7,9,10-hexahydroacridine-1,8(2H,5H)-dione in KBr

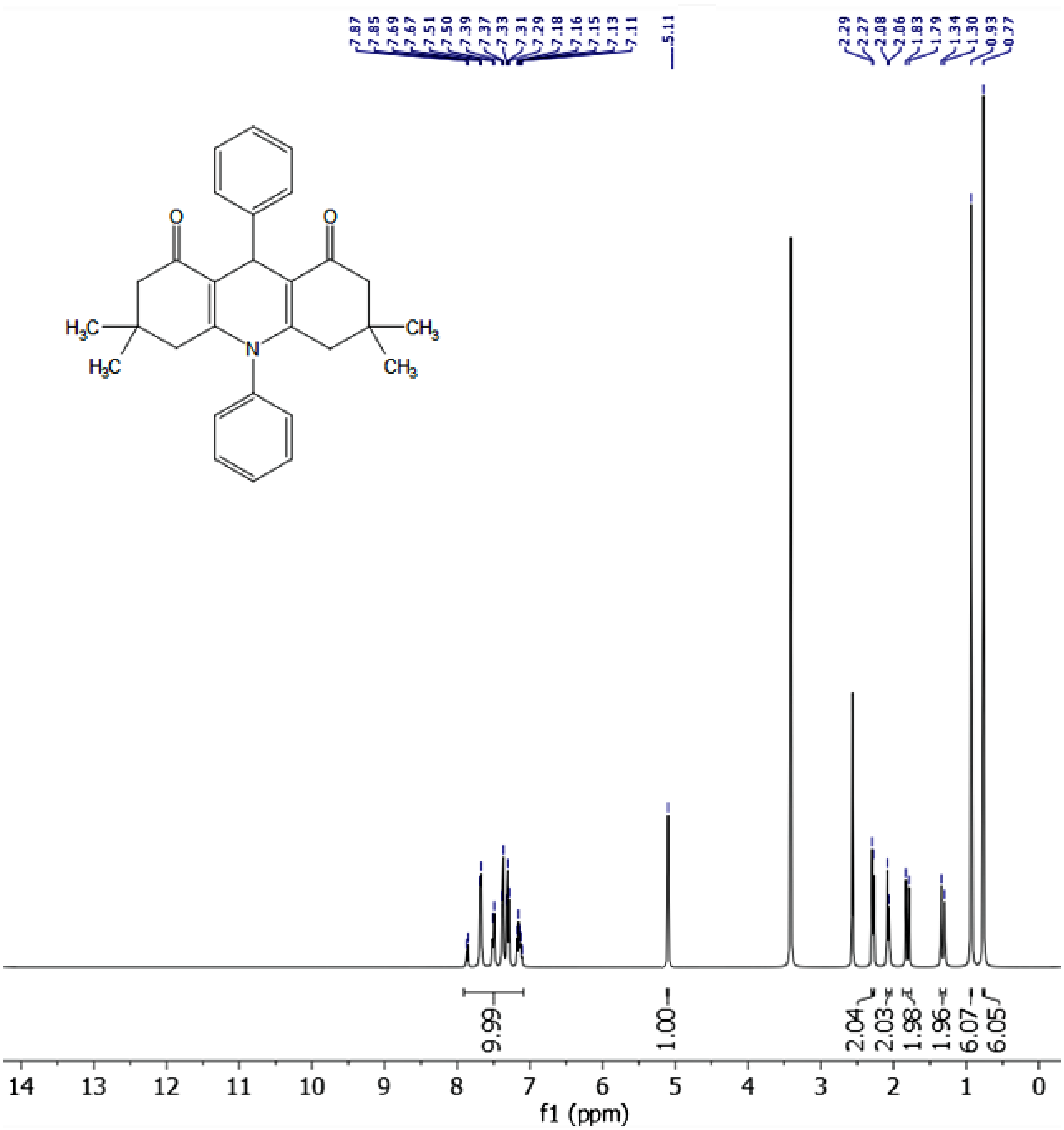

Figure S37: The ^{1}H NMR spectrum (400 MHz) of 3,3,6,6-tetramethyl-9,10-diphenyl-3,4,6,7,9,10-hexahydroacridine-1,8(2*H*,5*H*)-dione in DMSO-d_6

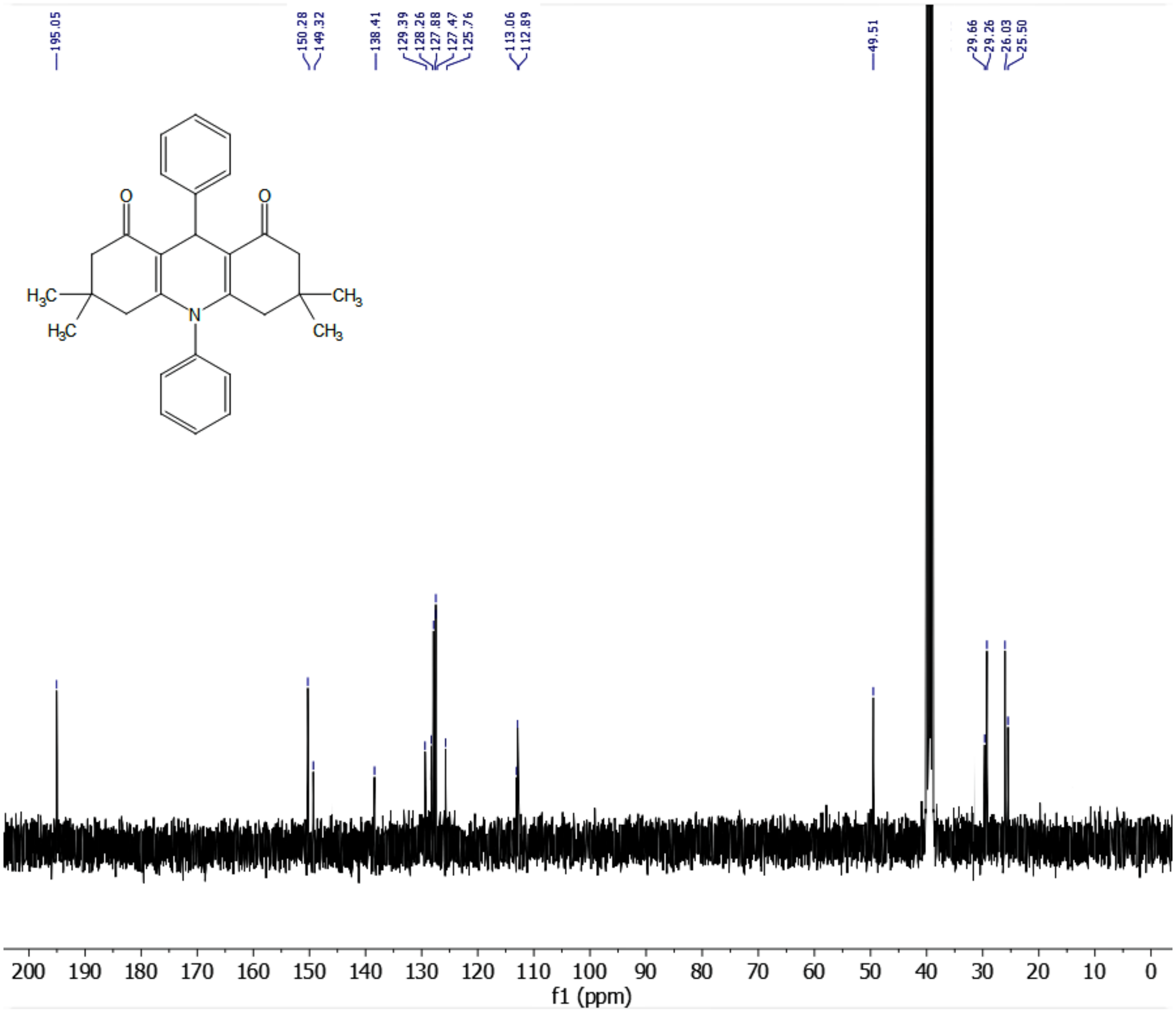

Figure S38: The ^{13}C NMR spectrum (101 MHz) of 3,3,6,6-tetramethyl-9,10-diphenyl-3,4,6,7,9,10-hexahydroacridine-1,8(2*H*,5*H*)-dione in DMSO-d_6

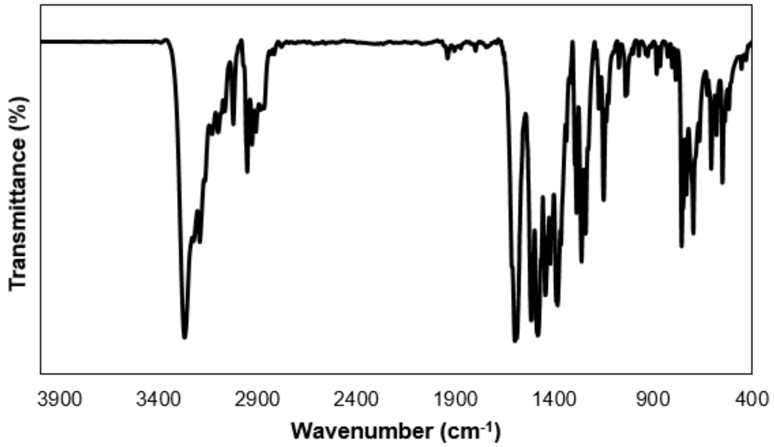

Figure S39: The FT-IR spectrum of 3,3,6,6-tetramethyl-9,10-diphenyl-3,4,6,7,9,10-hexahydroacridine-1,8(2*H*,5*H*)-dione in KBr

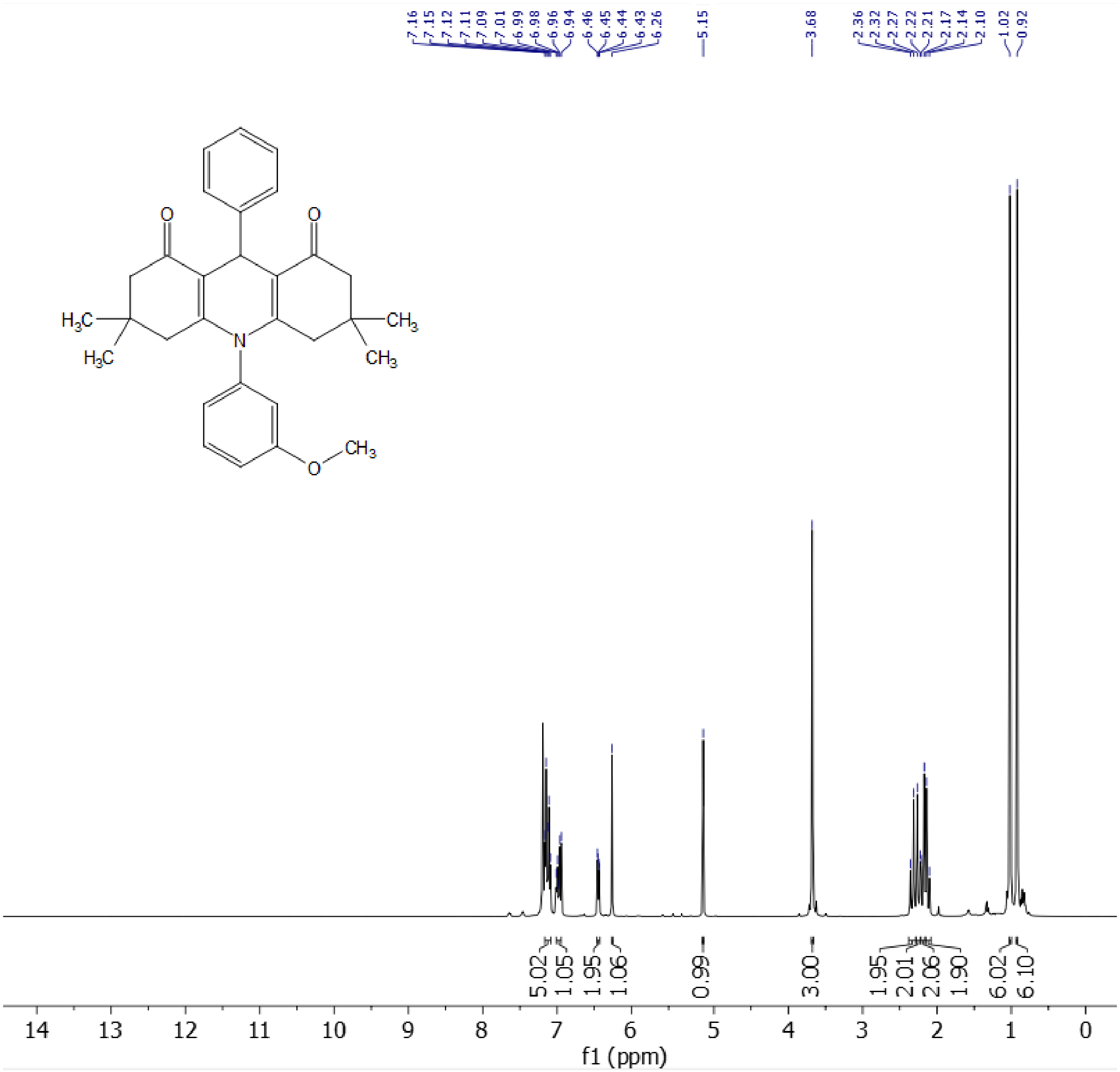

Figure S40: The ^{1}H NMR spectrum (400 MHz) of 10-(3-methoxyphenyl)-3,3,6,6-tetramethyl-9-phenyl-3,4,6,7,9,10-hexahydroacridine-1,8(2*H*,5*H*)-dione in $CDCl_3$

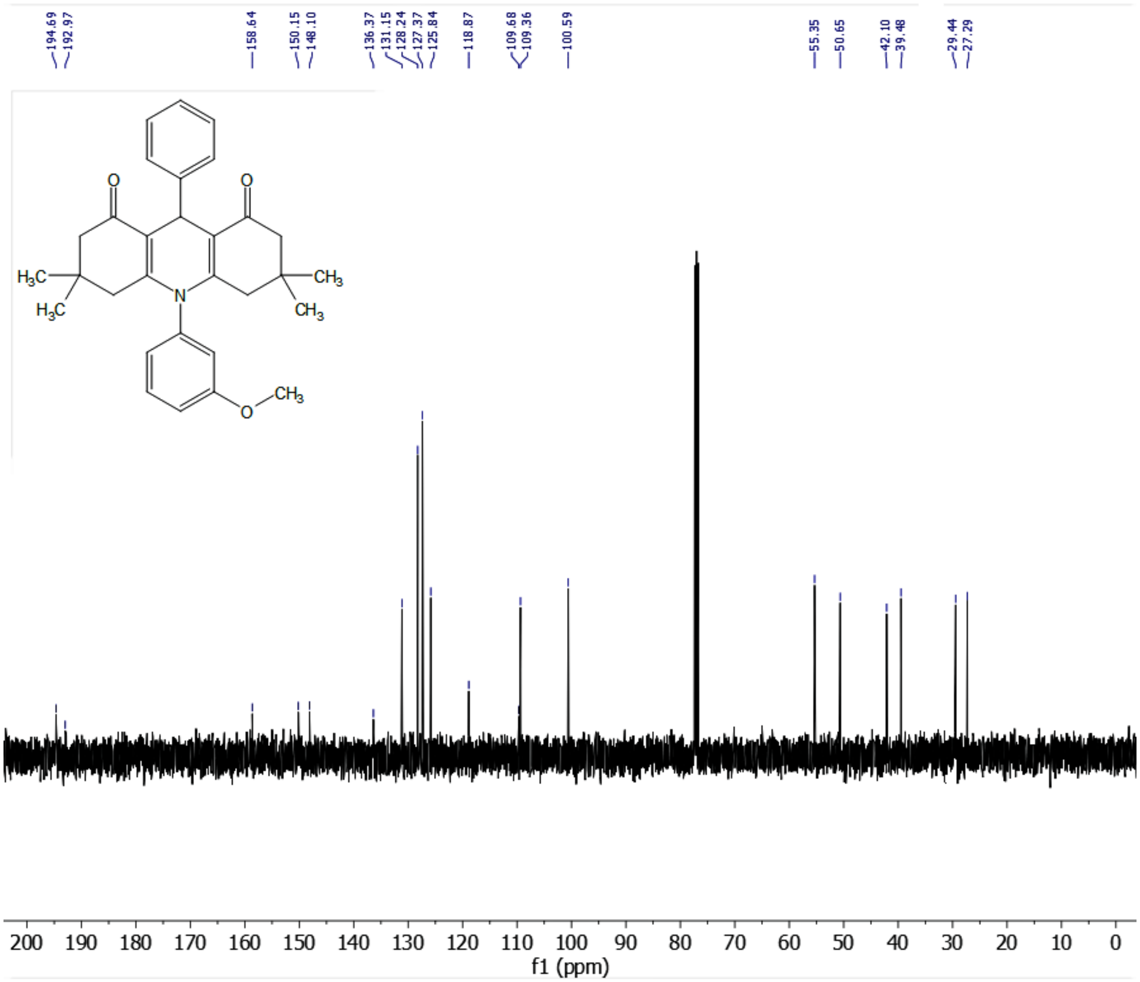

Figure S41: The ^{13}C NMR spectrum (101 MHz) of 10-(3-methoxyphenyl)-3,3,6,6-tetramethyl-9-phenyl-3,4,6,7,9,10-hexahydroacridine-1,8(2*H*,5*H*)-dione in $CDCl_3$

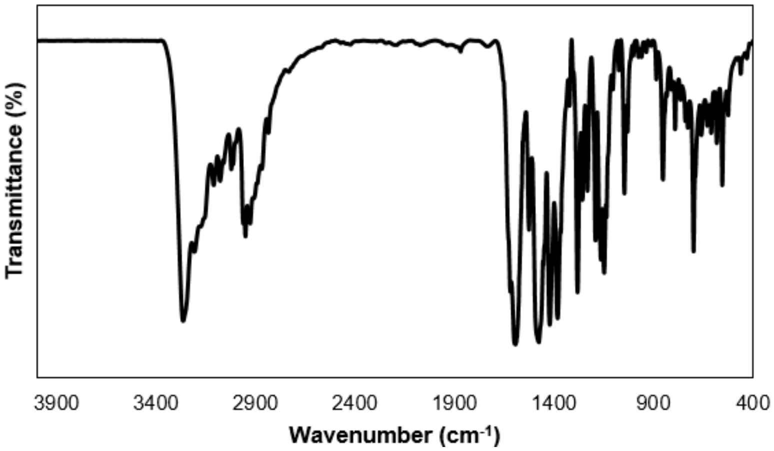

Figure S42: The FT-IR spectrum of 10-(3-methoxyphenyl)-3,3,6,6-tetramethyl-9-phenyl-3,4,6,7,9,10-hexahydroacridine-1,8(2*H*,5*H*)-dione in KBr

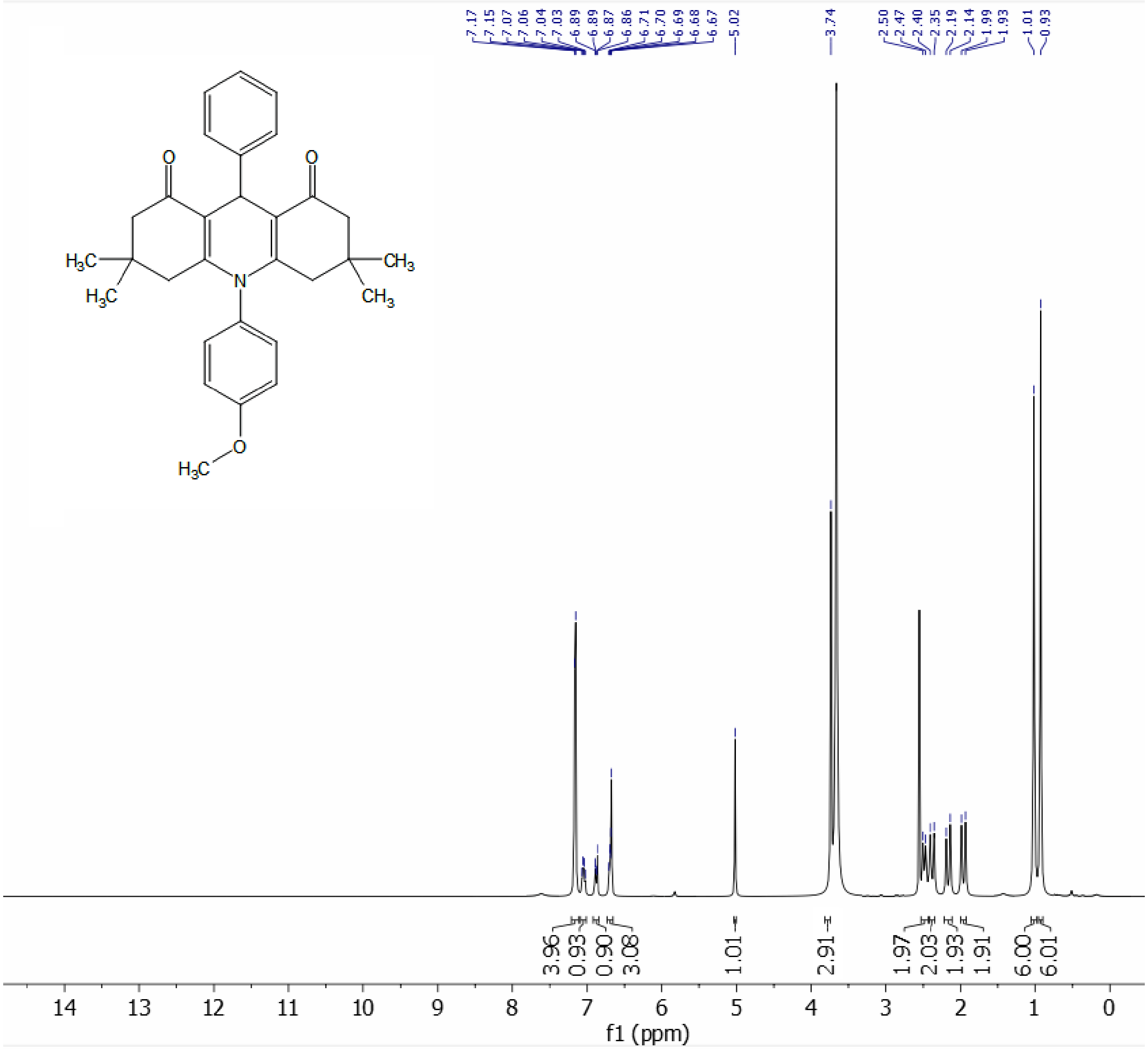

Figure S43: The ^{1}H NMR spectrum of (400 MHz) 10-(4-methoxyphenyl)-3,3,6,6-tetramethyl-9-phenyl-3,4,6,7,9,10-hexahydroacridine-1,8(2*H*,5*H*)-dione in DMSO-d_6

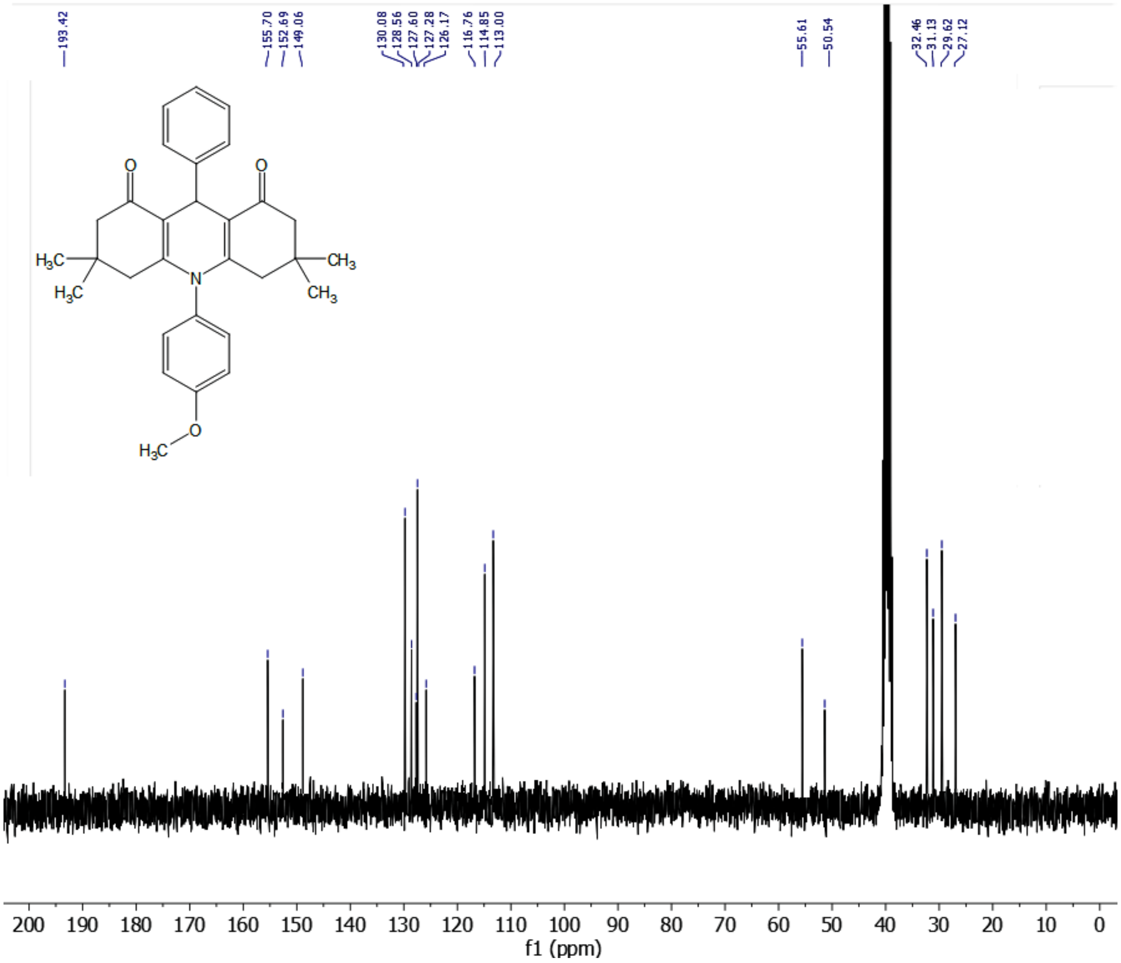

Figure S44: The ^{13}C NMR spectrum (101 MHz) of 10-(4-methoxyphenyl)-3,3,6,6-tetramethyl-9-phenyl-3,4,6,7,9,10-hexahydroacridine-1,8(2*H*,5*H*)-dione in DMSO-d_6

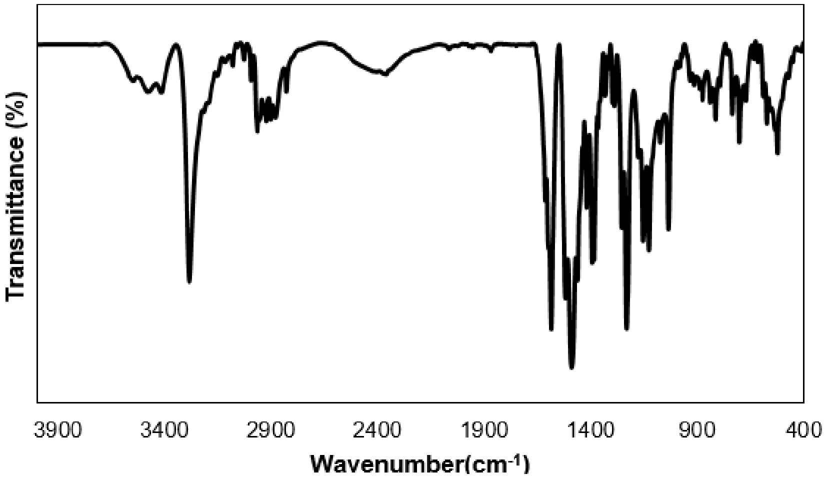

Figure S45: The FT-IR spectrum of 10-(4-methoxyphenyl)-3,3,6,6-tetramethyl-9-phenyl-3,4,6,7,9,10-hexahydroacridine-1,8(2*H*,5*H*)-dione in KBr

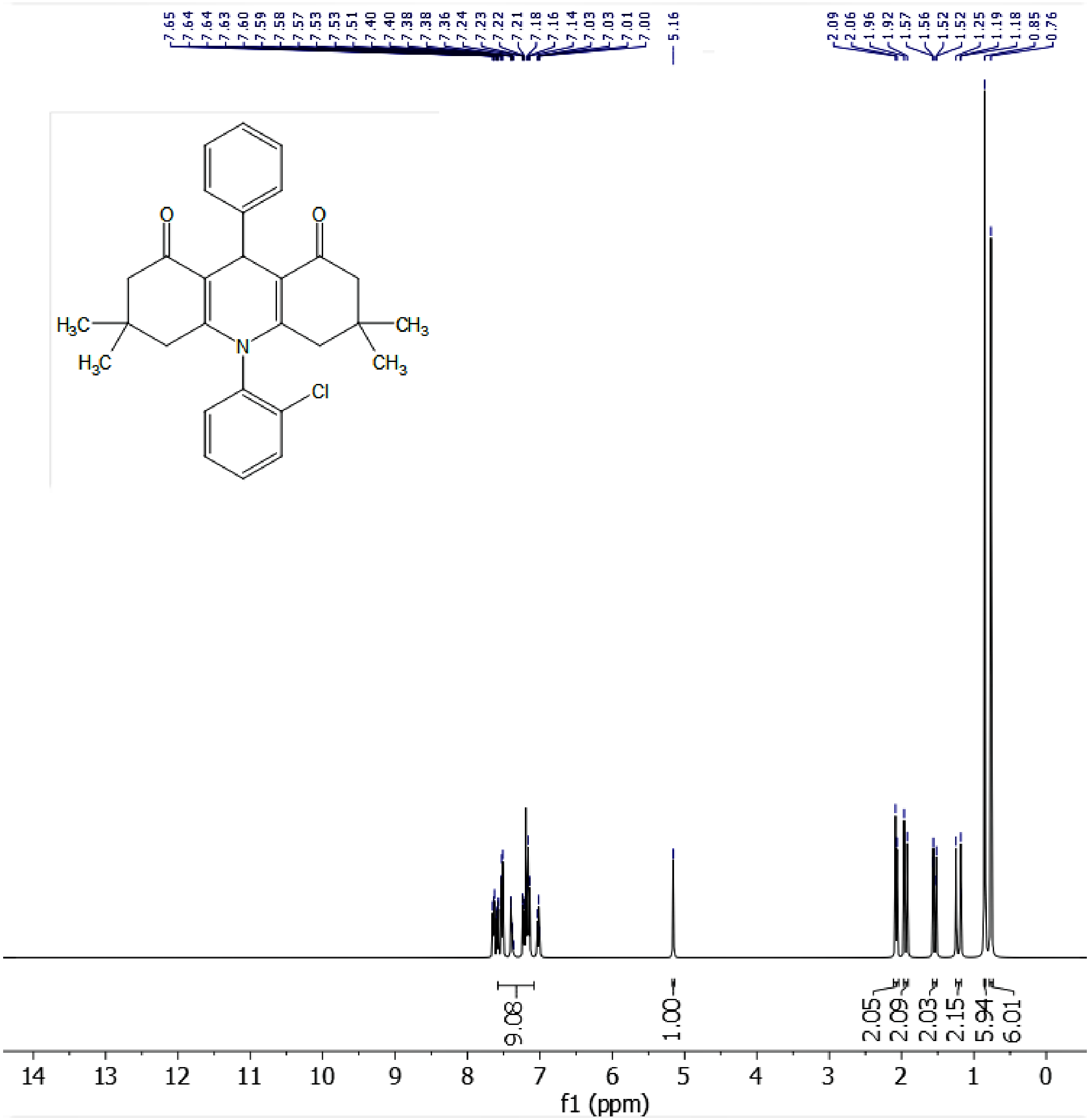

Figure S46: The ^{1}H NMR spectrum (400 MHz) of 10-(2-chlorophenyl)-3,3,6,6-tetramethyl-9-phenyl-3,4,6,7,9,10-hexahydroacridine-1,8(2*H*,5*H*)-dione in $CDCl_3$

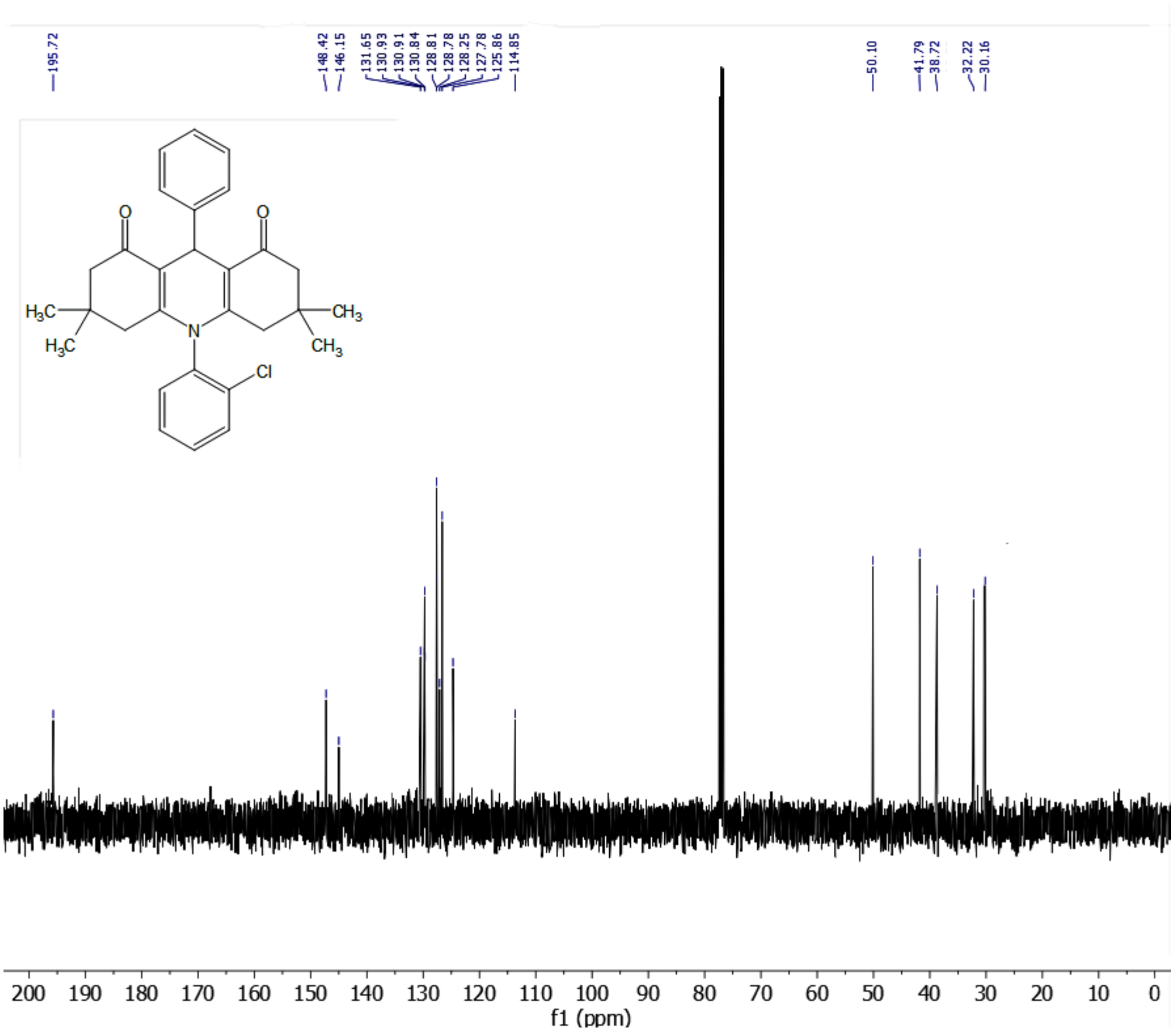

Figure S47: The ^{13}C NMR spectrum (101 MHz) of 10-(2-chlorophenyl)-3,3,6,6-tetramethyl-9-phenyl-3,4,6,7,9,10-hexahydroacridine-1,8(2*H*,5*H*)-dione in $CDCl_3$

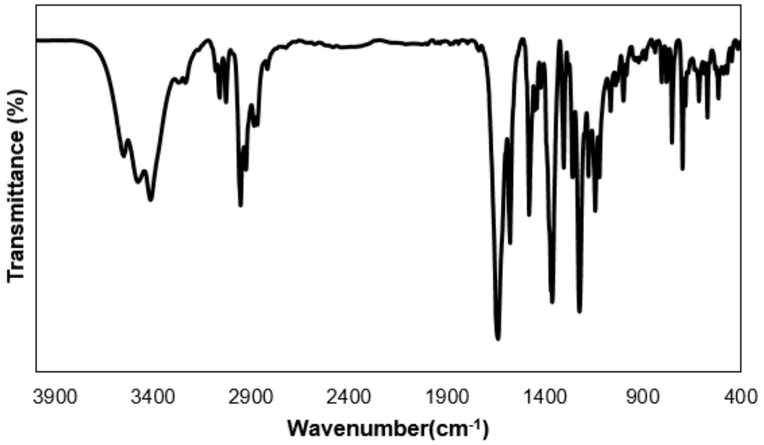

Figure S48: The FT-IR spectrum of 10-(2-chlorophenyl)-3,3,6,6-tetramethyl-9-phenyl-3,4,6,7,9,10-hexahydroacridine-1,8(2*H*,5*H*)-dione in KBr

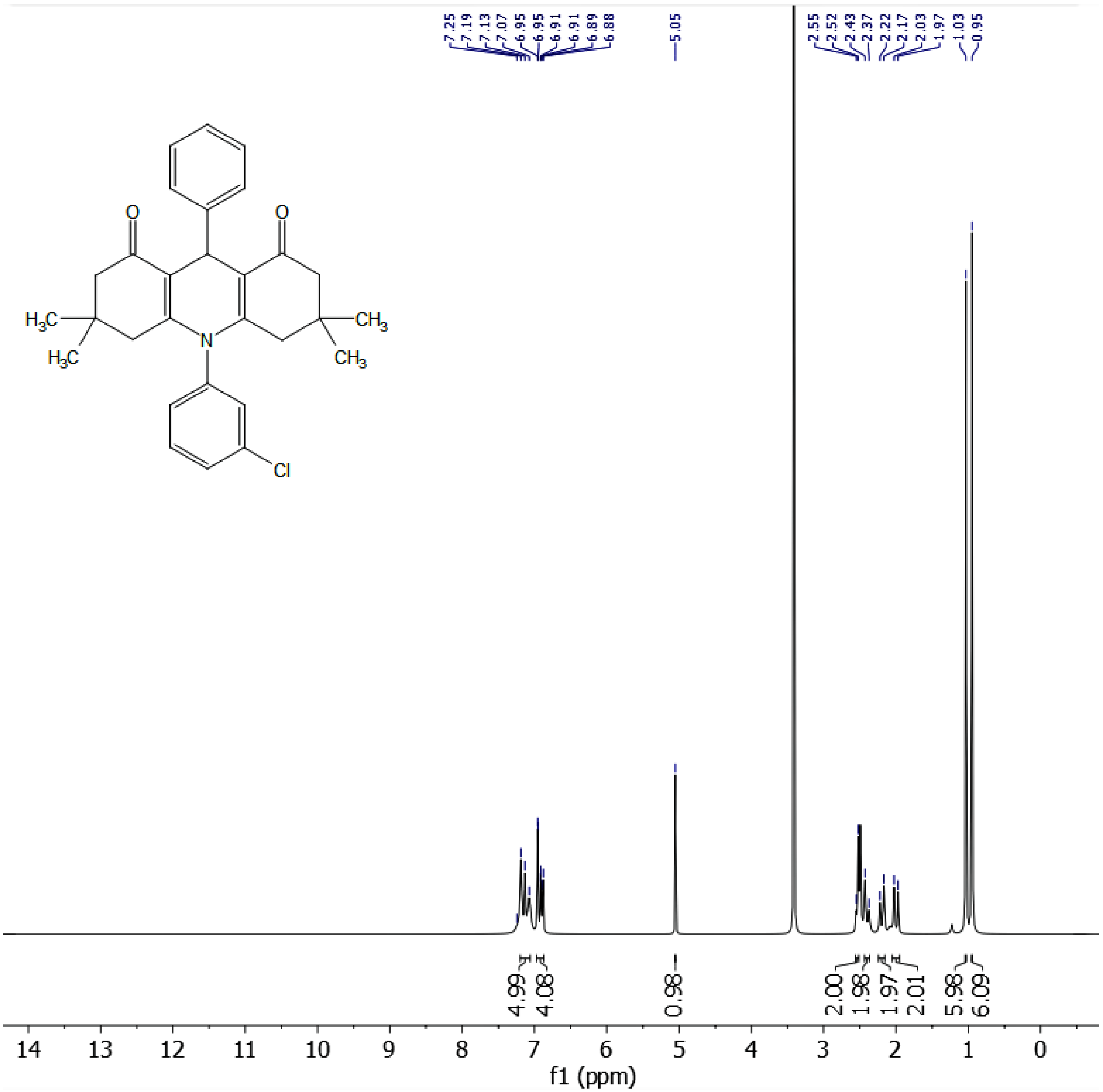

Figure S49: The ^{1}H NMR spectrum of (300 MHz) 10-(3-chlorophenyl)-3,3,6,6-tetramethyl-9-phenyl-3,4,6,7,9,10-hexahydroacridine-1,8(2*H*,5*H*)-dione in DMSO-d_6

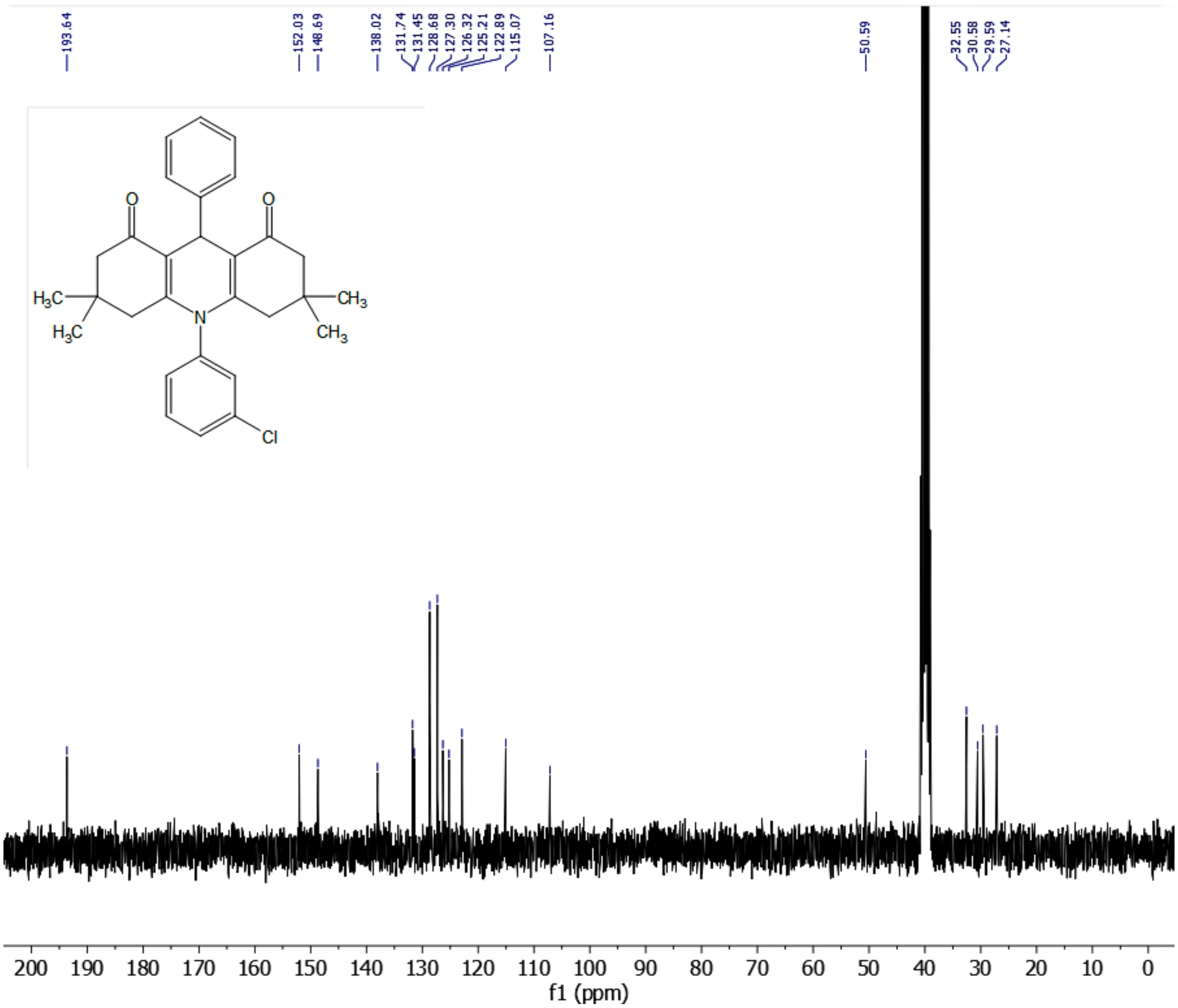

Figure S50: The ^{13}C NMR spectrum (76 MHz) of 10-(3-chlorophenyl)-3,3,6,6-tetramethyl-9-phenyl-3,4,6,7,9,10-hexahydroacridine-1,8(2*H*,5*H*)-dione in DMSO-d_6

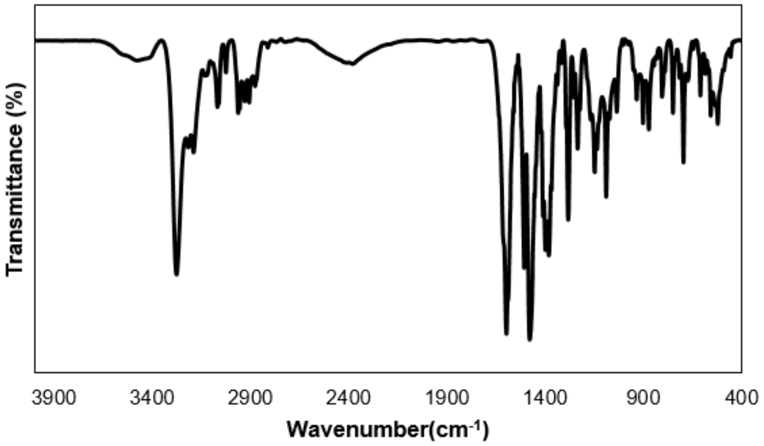

Figure S51: The FT-IR spectrum of 10-(3-chlorophenyl)-3,3,6,6-tetramethyl-9-phenyl-3,4,6,7,9,10-hexahydroacridine-1,8(2*H*,5*H*)-dione in KBr

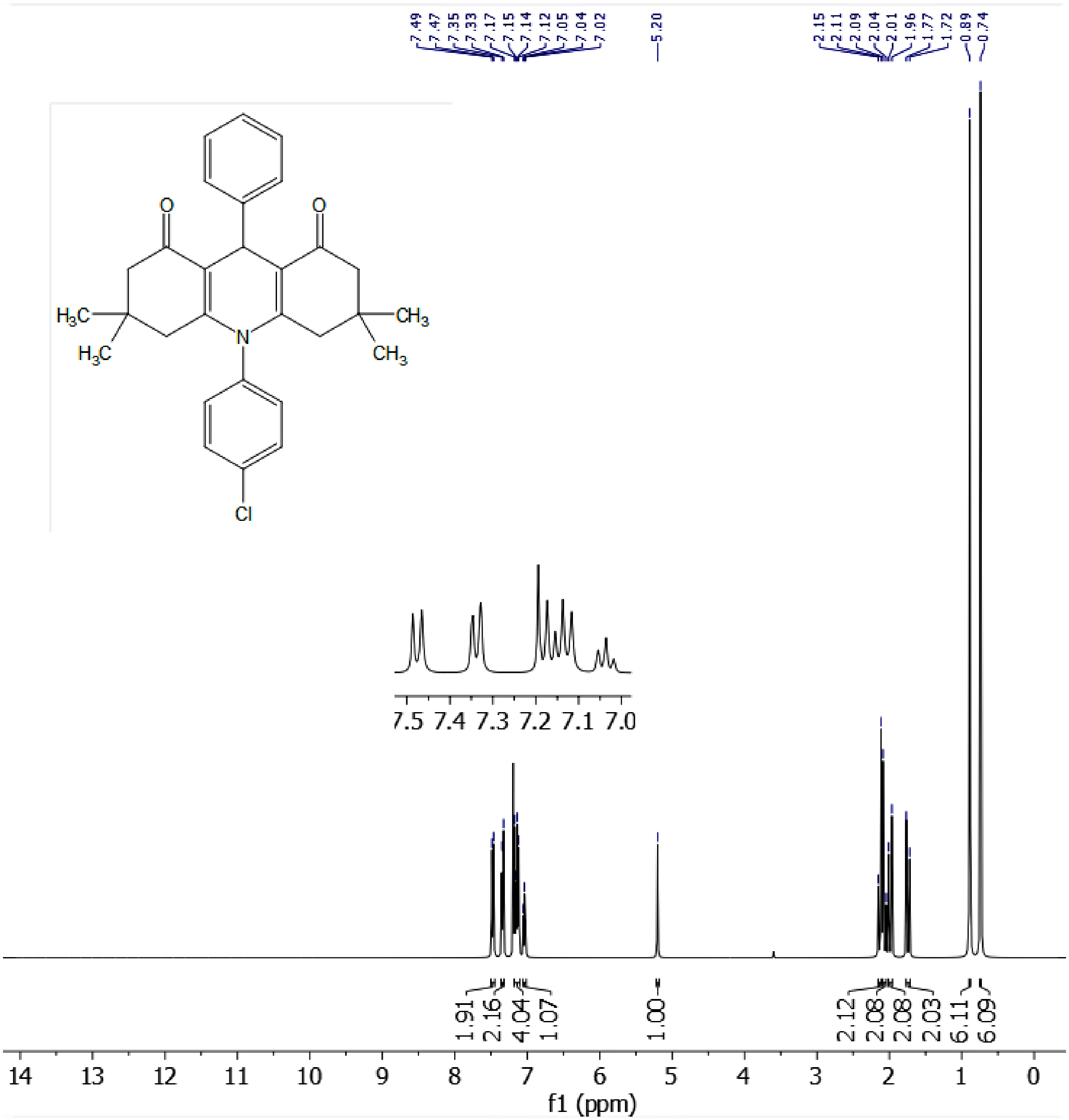

Figure S52: The ^1H NMR spectrum (400 MHz) of 10-(4-chlorophenyl)-3,3,6,6-tetramethyl-9-phenyl-3,4,6,7,9,10-hexahydroacridine-1,8(2*H*,5*H*)-dione in $CDCl_3$

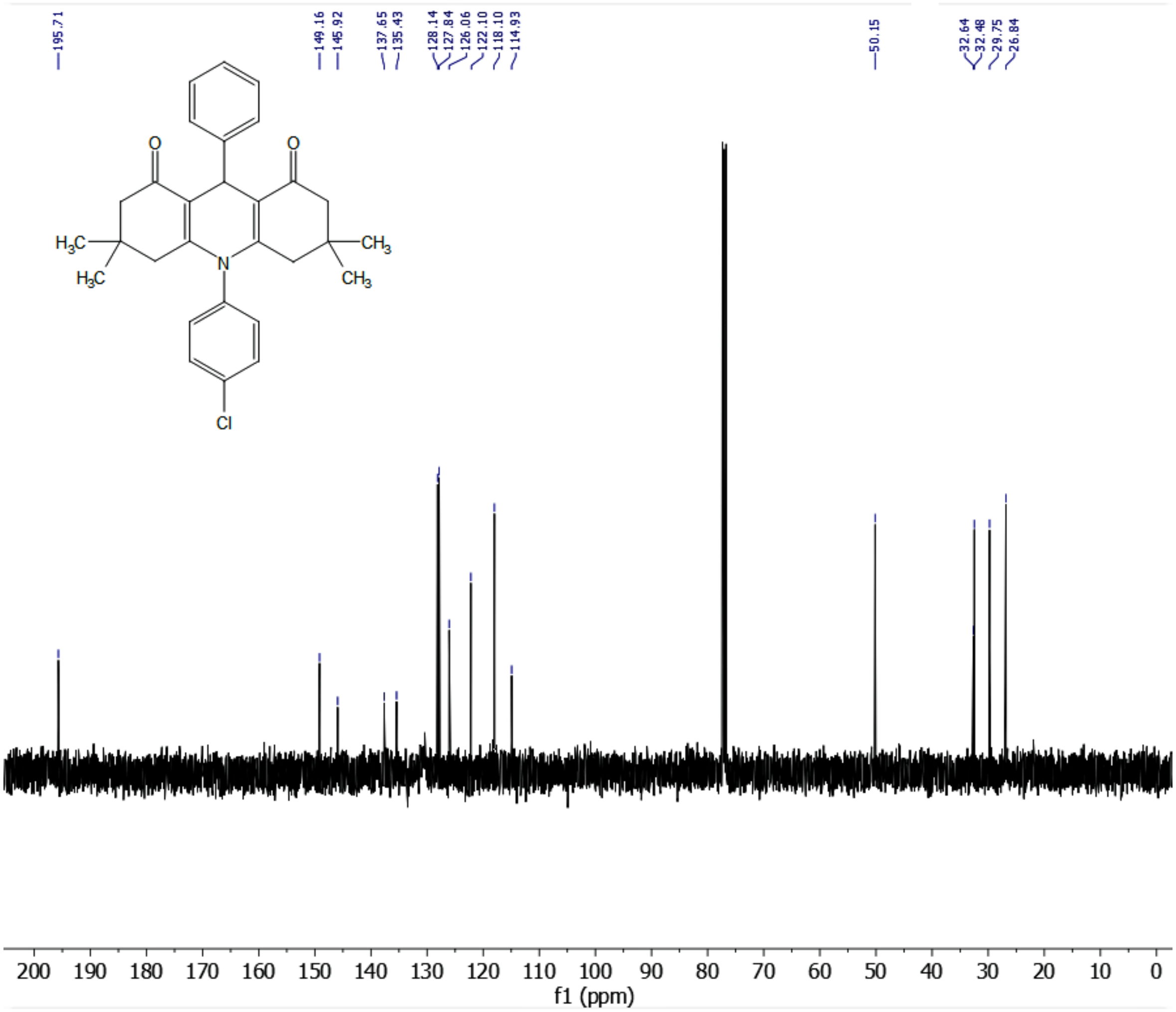

Figure S53: The ^{13}C NMR spectrum (101 MHz) of 10-(4-chlorophenyl)-3,3,6,6-tetramethyl-9-phenyl-3,4,6,7,9,10-hexahydroacridine-1,8(2*H*,5*H*)-dione in $CDCl_3$

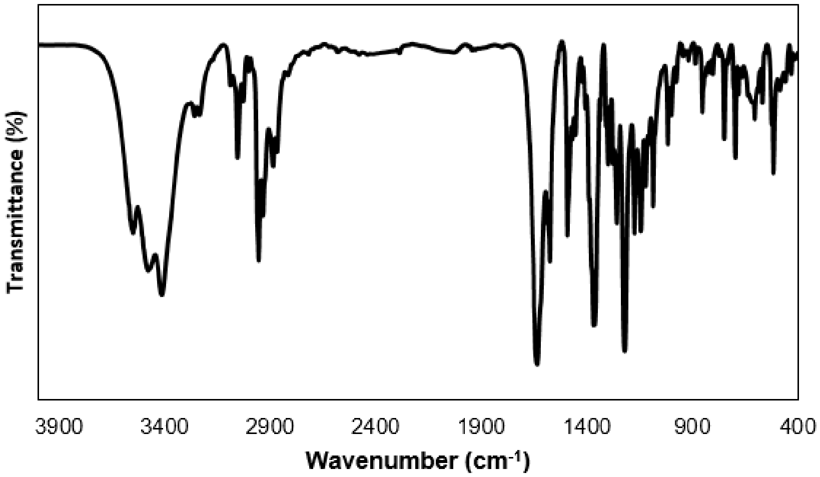

Figure S54: The FT-IR spectrum of 10-(4-chlorophenyl)-3,3,6,6-tetramethyl-9-phenyl-3,4,6,7,9,10-hexahydroacridine-1,8(2*H*,5*H*)-dione in KBr

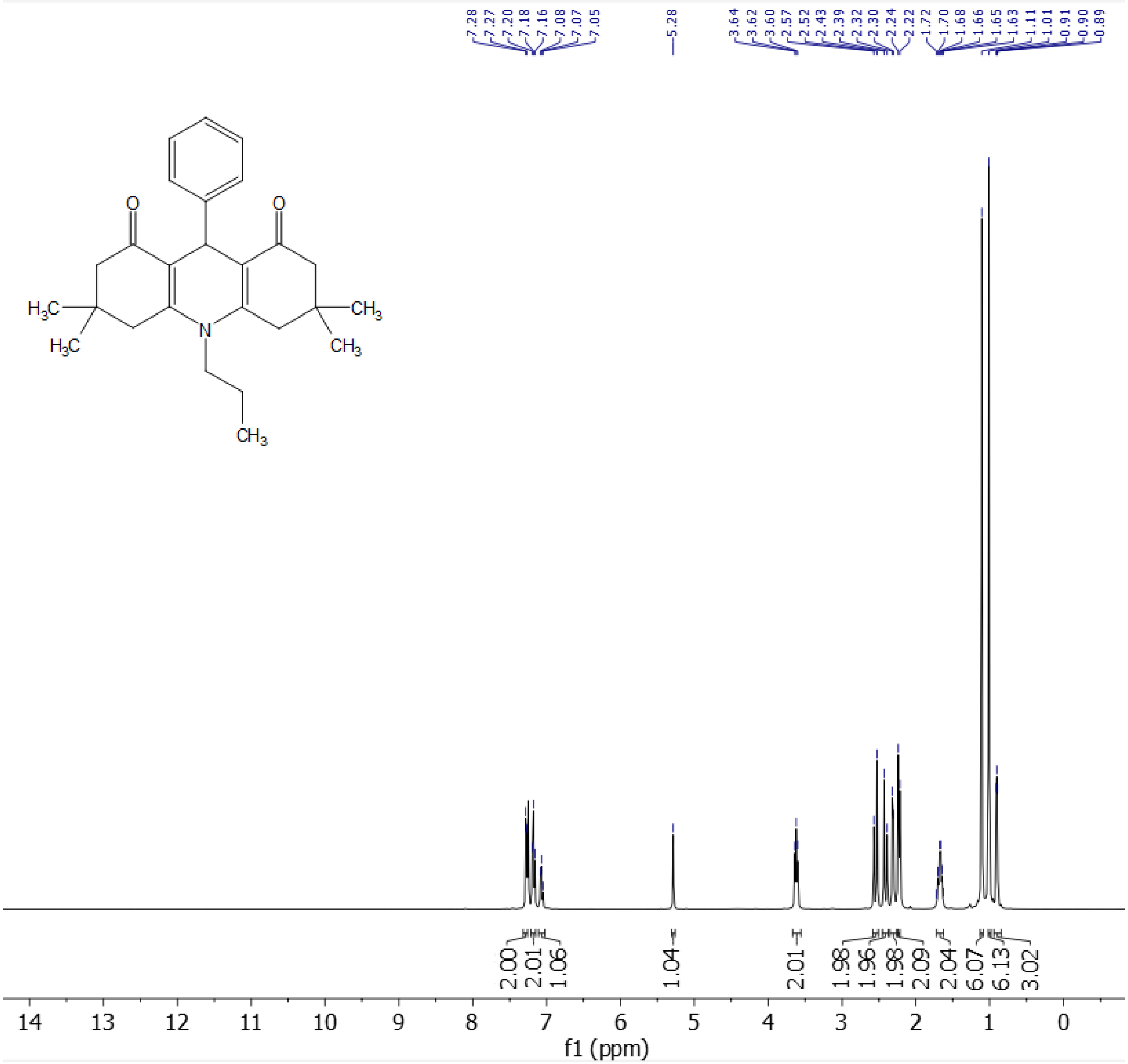

Figure S55: The ^{1}H NMR spectrum (400 MHz) of 3,3,6,6-tetramethyl-9-phenyl-10-propyl-3,4,6,7,9,10-hexahydroacridine-1,8(2H,5H)-dione in $CDCl_3$

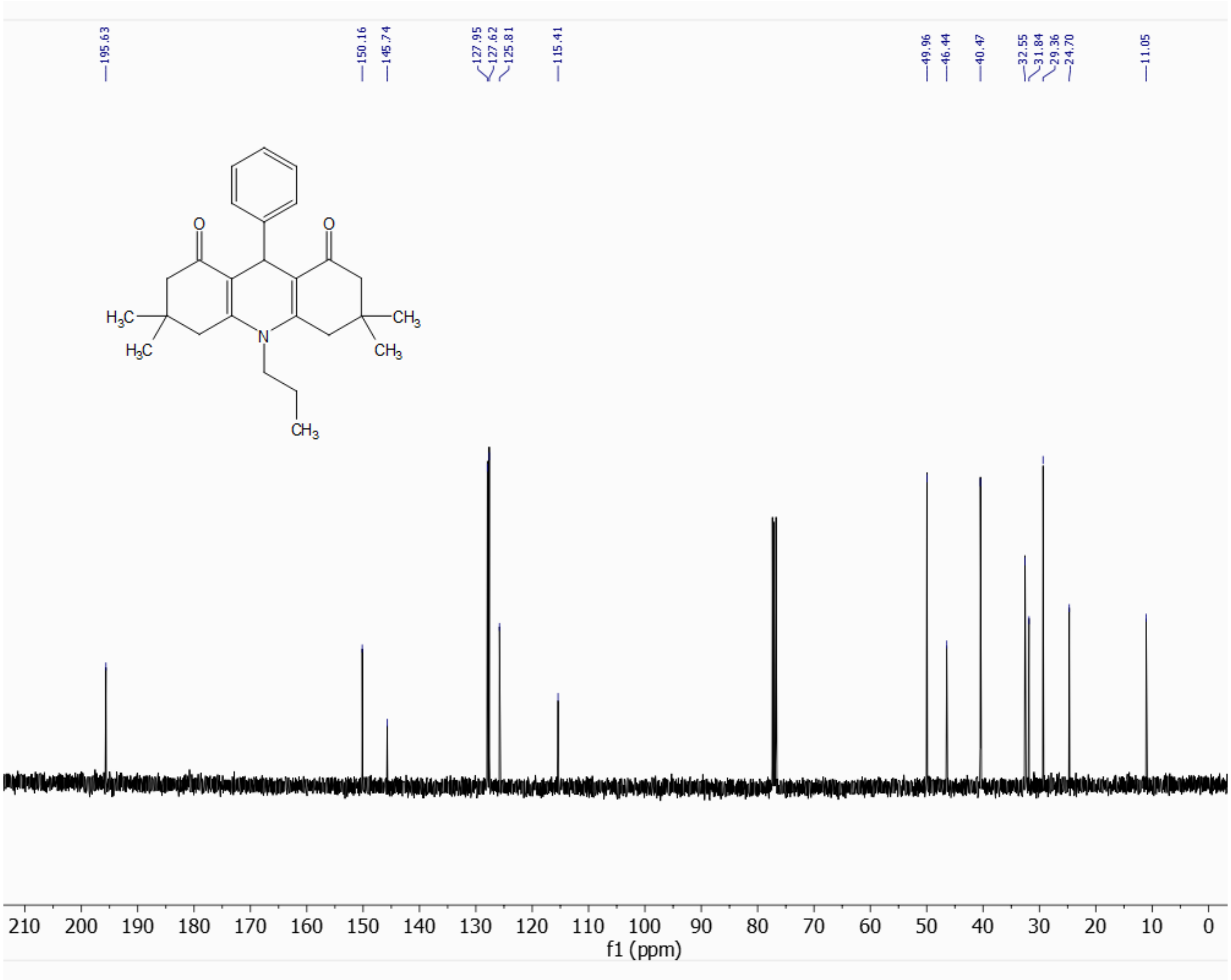

Figure S56: The ^{13}C NMR spectrum (101 MHz) of 3,3,6,6-tetramethyl-9-phenyl-10-propyl-3,4,6,7,9,10-hexahydroacridine-1,8(2H,5H)-dione in $CDCl_3$

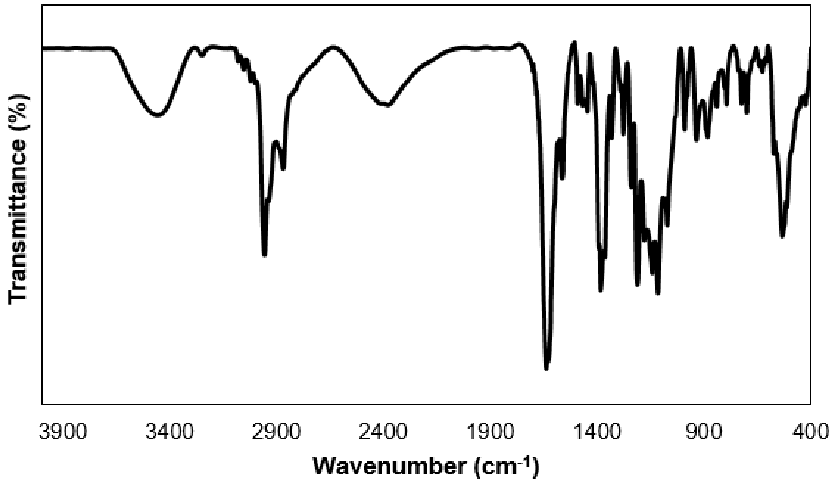

Figure S57: The FT-IR spectrum of 3,3,6,6-tetramethyl-9-phenyl-10-propyl-3,4,6,7,9,10-hexahydroacridine-1,8(2H,5H)-dione in KBr

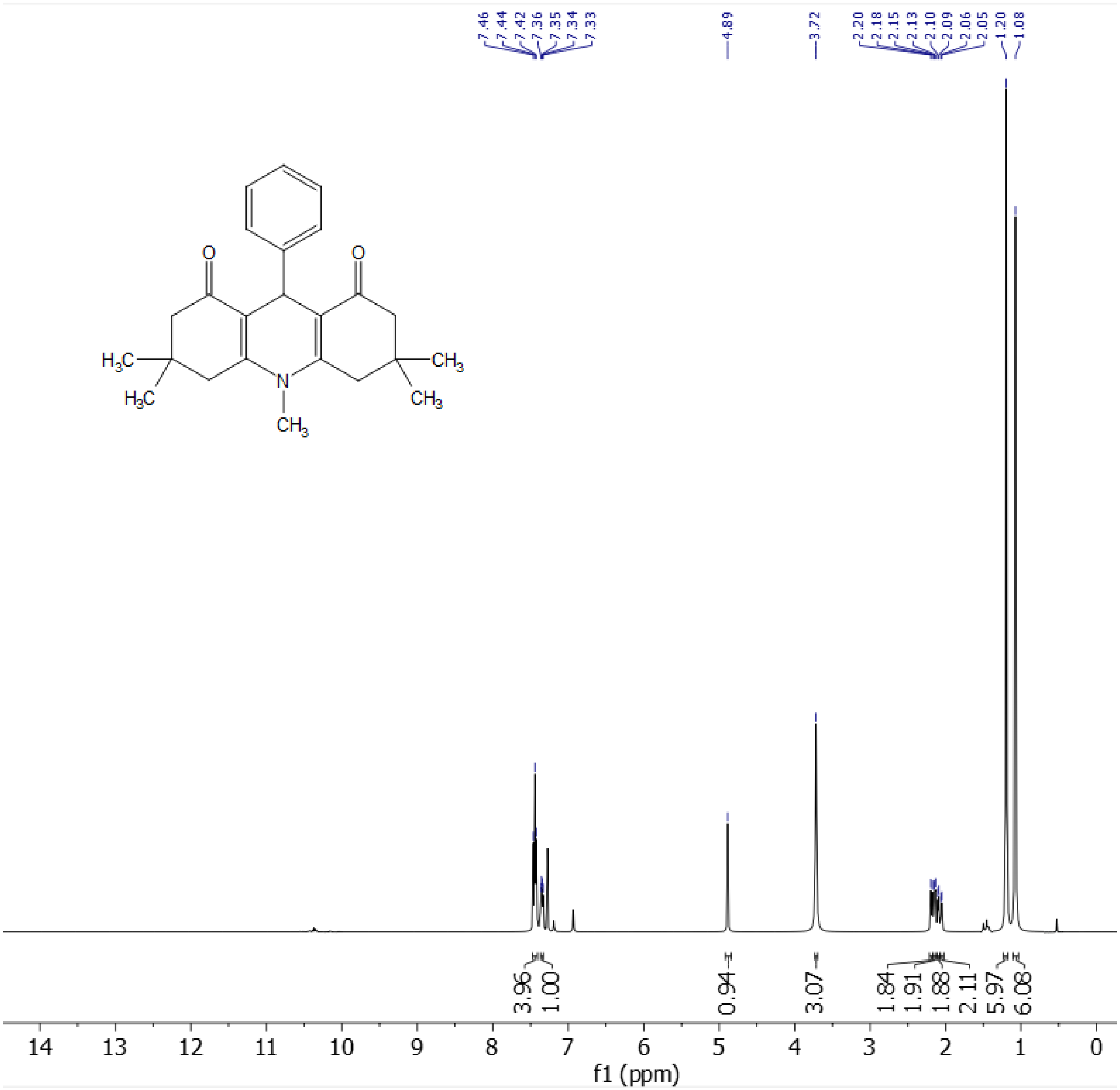

Figure S58: The ^{1}H NMR spectrum (400 MHz) of 3,3,6,6,10-pentamethyl-9-phenyl-3,4,6,7,9,10-hexahydroacridine-1,8(2H,5H)-dione in $CDCl_3$

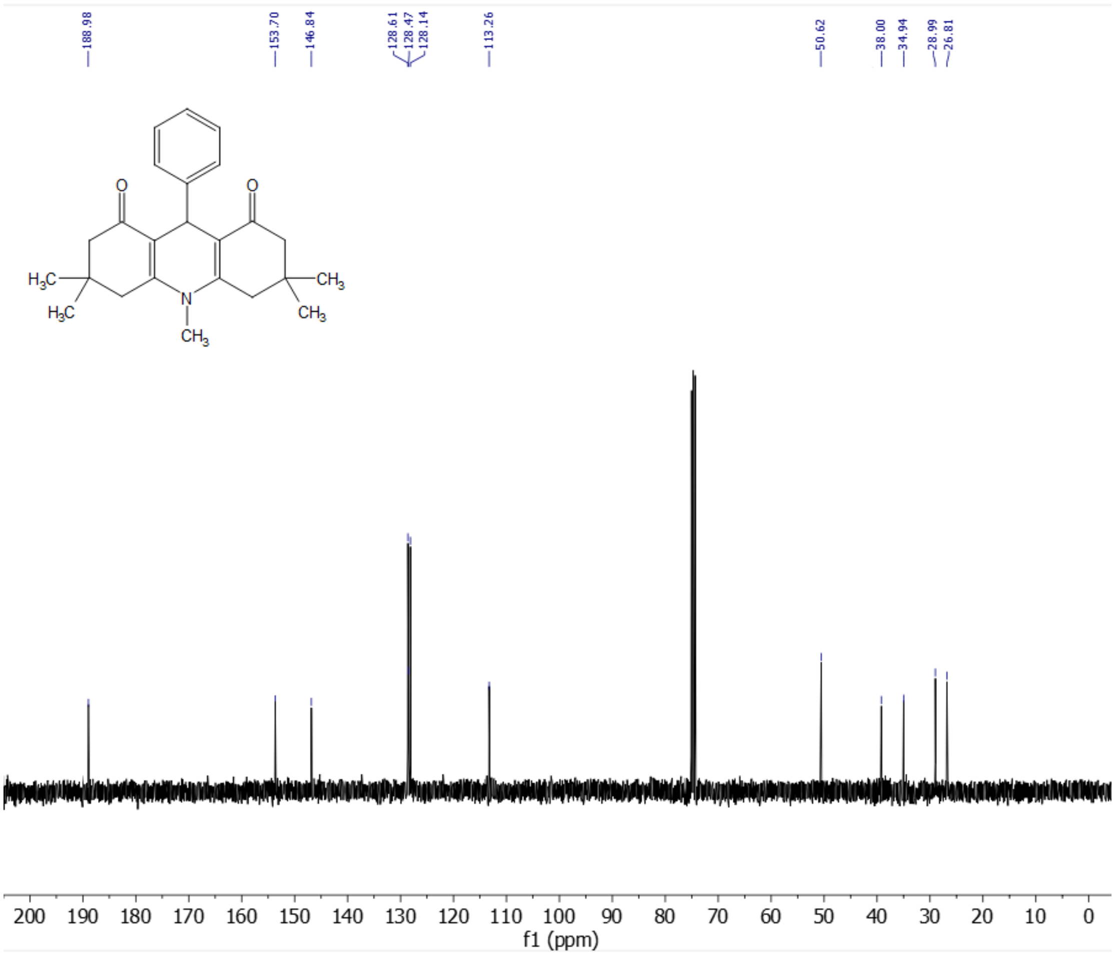

Figure S59: The ^{13}C NMR spectrum (101 MHz) of 3,3,6,6,10-pentamethyl-9-phenyl-3,4,6,7,9,10-hexahydroacridine-1,8(2H,5H)-dione in $CDCl_3$

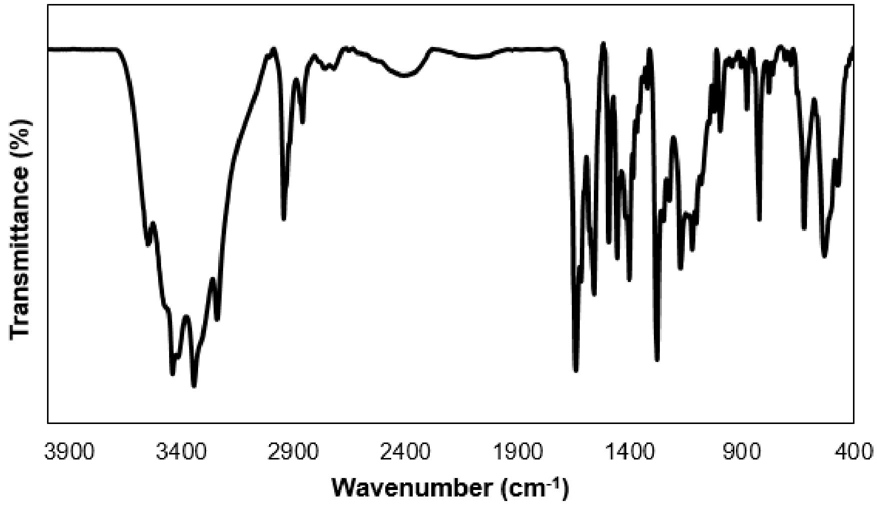

Figure S60: The FT-IR spectrum of 3,3,6,6,10-pentamethyl-9-phenyl-3,4,6,7,9,10-hexahydroacridine-1,8(2H,5H)-dione in KBr

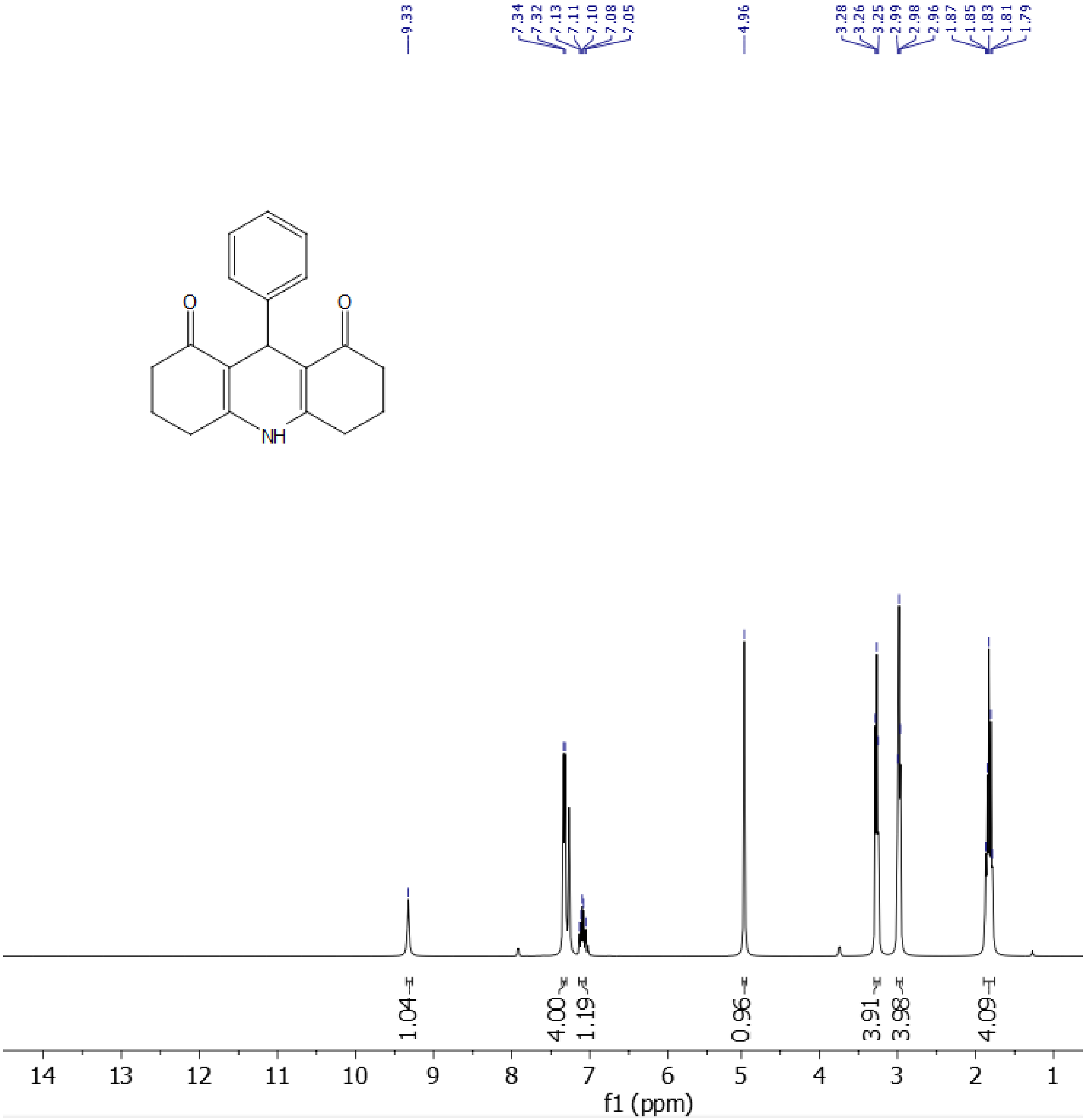

Figure S61: The ^{1}H NMR spectrum (400 MHz) of 9-phenyl-3,4,6,7,9,10-hexahydroacridine-1,8(2H,5H)-dione in $CDCl_3$

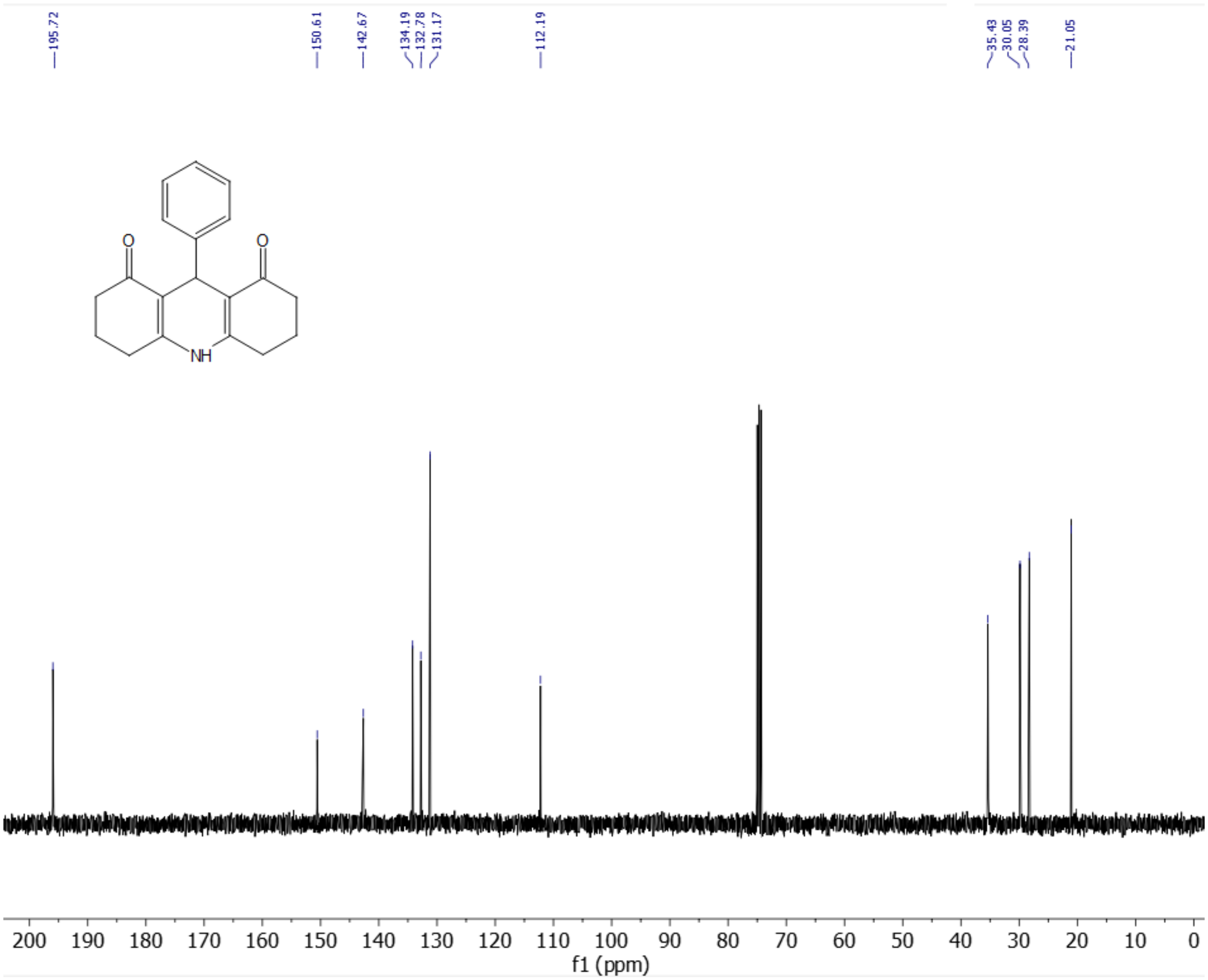

Figure S62: The ^{13}C NMR spectrum (101 MHz) of 9-phenyl-3,4,6,7,9,10-hexahydroacridine-1,8(2H,5H)-dione in $CDCl_3$

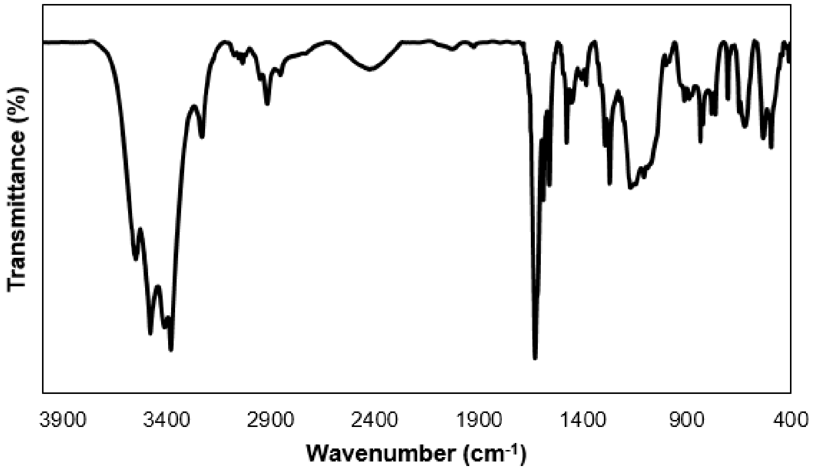

Figure S63: The FT-IR spectrum of 9-phenyl-3,4,6,7,9,10-hexahydroacridine-1,8(2H,5H)-dione in KBr

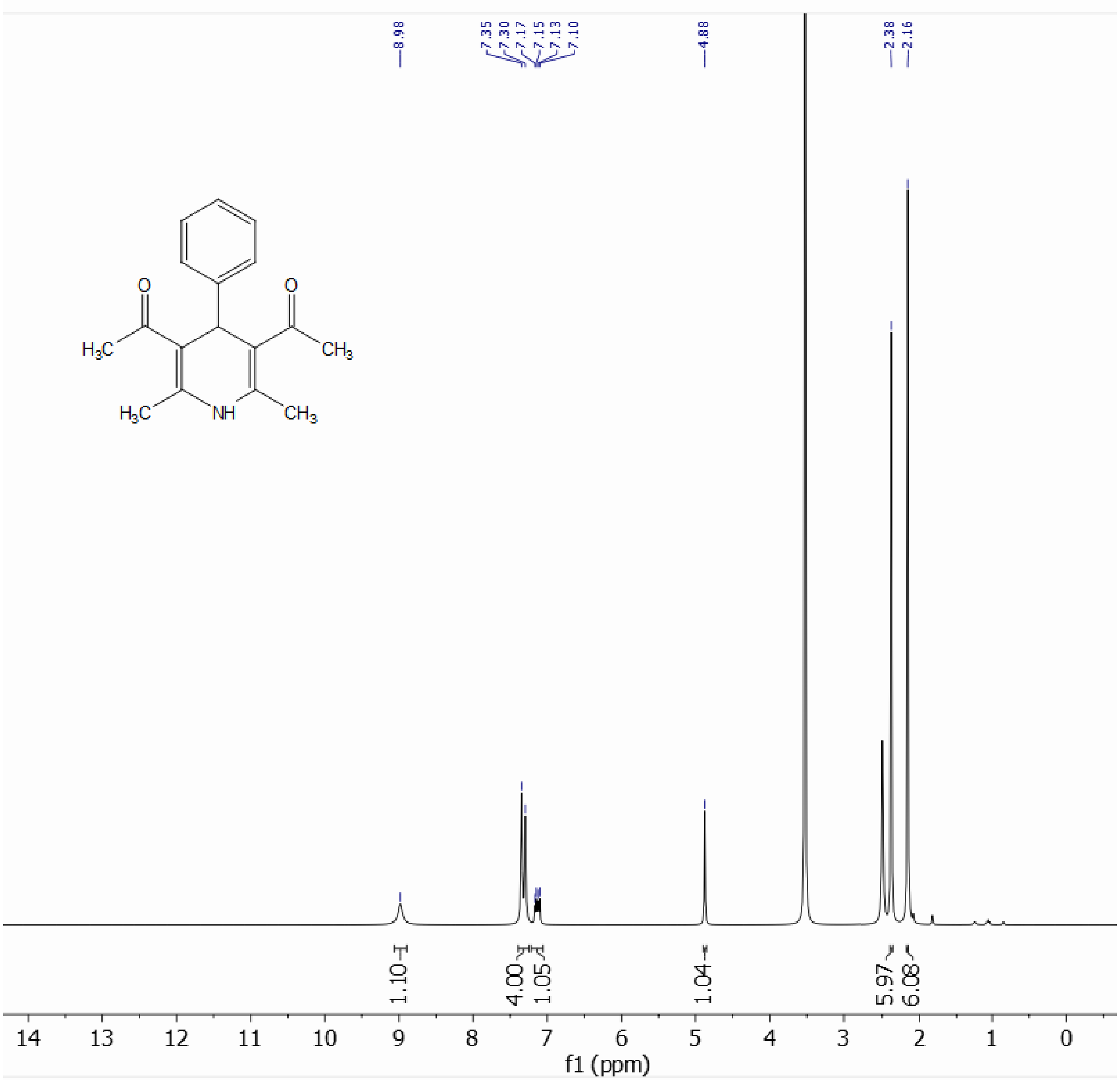

Figure S64: The ^{1}H NMR spectrum (400 MHz) of 1,1'-(2,6-dimethyl-4-phenyl-1,4-dihydropyridine-3,5-diyl)bis(ethan-1-one) in DMSO-d_6

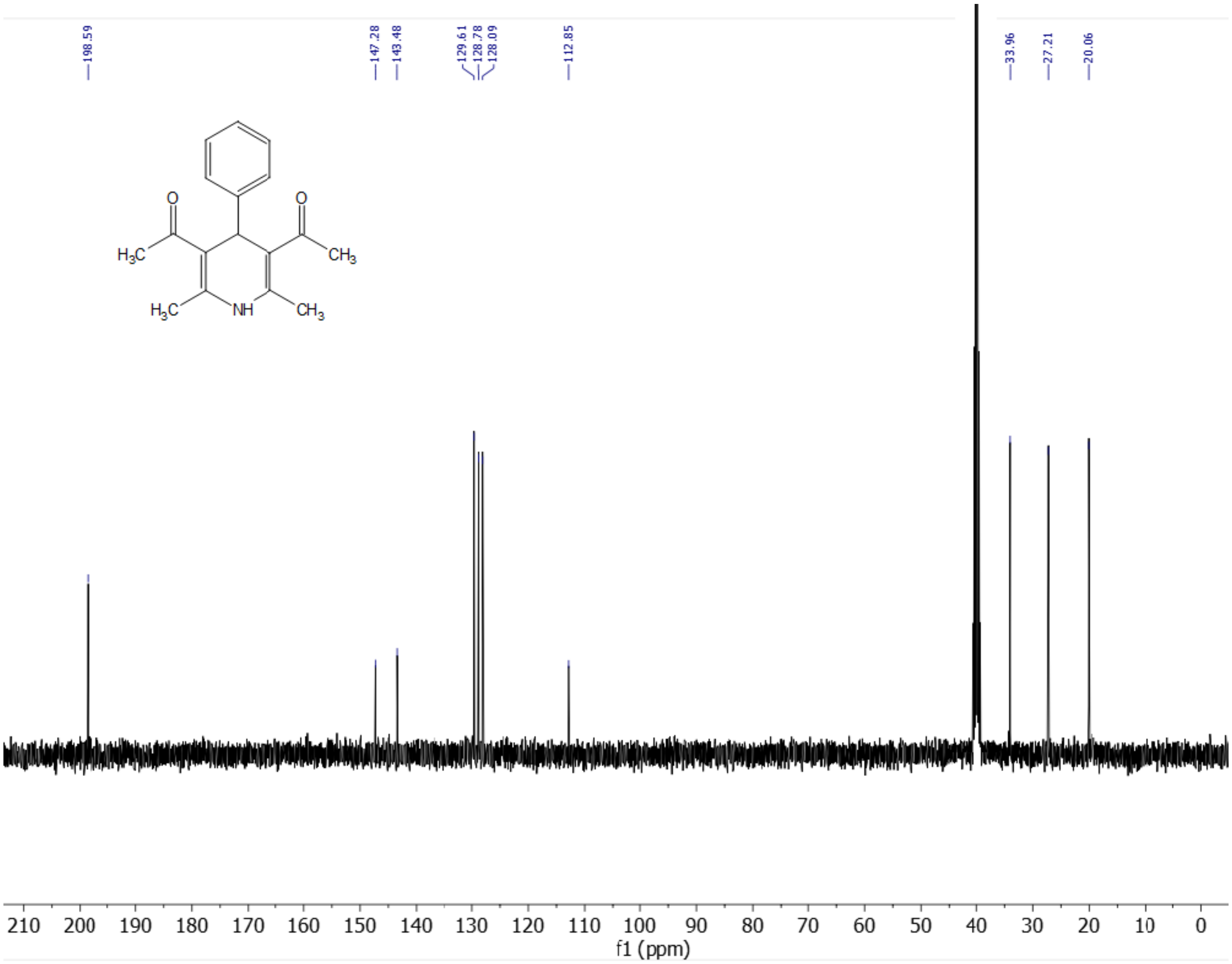

Figure S65: The ^{13}C NMR spectrum (101 MHz) of 1,1'-(2,6-dimethyl-4-phenyl-1,4-dihydropyridine-3,5-diyl)bis(ethan-1-one) in DMSO-d_6

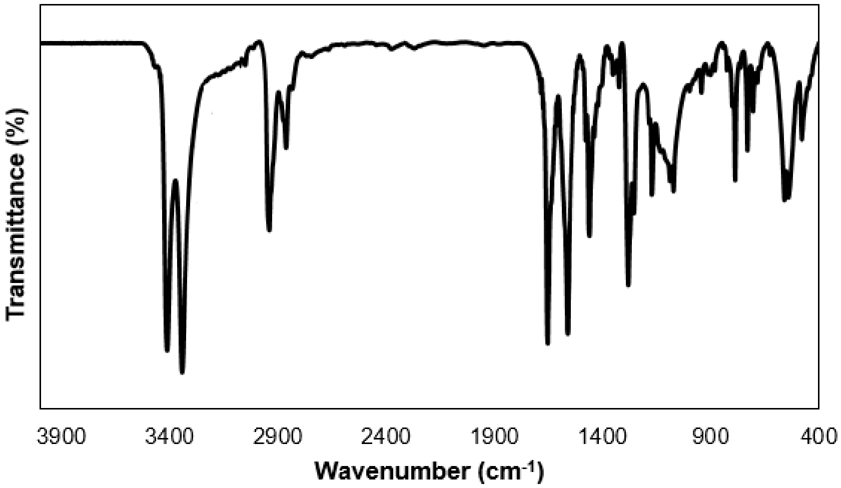

Figure S66: The FT-IR spectrum of 1,1'-(2,6-dimethyl-4-phenyl-1,4-dihydropyridine-3,5-diyl)bis(ethan-1-one) in KBr

References

[1] Aher, D. S.; Khillare, K. R.; Shankarwar, S. G. Incorporation of Keggin-Based $H_3PW_7Mo_5O_{40}$ into Bentonite: Synthesis, Characterization and Catalytic Applications. *RSC Advances* **2021**, *11* (19), 11244-11254. https://doi.org/10.1039/d1ra01179k.

[2] Zolfigol, M. A.; Karimi, F.; Yarie, M.; Torabi, M. Catalytic Application of Sulfonic acid-functionalized titana-coated Magnetic Nanoparticles for the Preparation of 1,8-dioxodecahydroacridines and 2,4,6-triarylpyridines via anomeric-based Oxidation. *Applied Organometallic Chemistry* **2017**, *32* (2). https://doi.org/10.1002/aoc.4063.

[3] Zhu, A.; Liu, R.; Du, C.; Li, L. Betainium-Based Ionic Liquids Catalyzed Multicomponent Hantzsch Reactions for the Efficient Synthesis of Acridinediones. *RSC Advances* **2017**, *7* (11), 6679-6684. https://doi.org/10.1039/c6ra25709g.

[4] Navarro, C. A.; Sierra, C. A.; Ochoa-Puentes, C. Evaluation of Sodium Acetate trihydrate–urea DES As a Benign Reaction Media for the Biginelli Reaction. Unexpected Synthesis of methylenebis(3-Hydroxy-5,5-Dimethylcyclohex-2-enones), Hexahydroxanthene-1,8-Diones and Hexahydroacridine-1,8-Diones. *RSC Advances* **2016**, *6* (70), 65355-65365. https://doi.org/10.1039/c6ra13848a.

[5] Tiwari, K. N.; Uttam, M. R.; Kumari, P.; Vatsa, P.; Prabhakaran, S. M. Efficient Synthesis of Acridinediones in Aqueous Media. *Synthetic Communications* **2017**, *47* (10), 1013-1019. https://doi.org/10.1080/00397911.2017.1304556.

[6] Eidi, E.; Kassaee, M. Z.; Nasresfahani, Z. Nanocrystalline TiO_2, via Green Combustion Synthesis, As an Efficient and Reusable Catalyst for the Preparation of 1,8-Dioxooctahydroxanthenes and 1,8-Dioxodecahydroacridines. *Applied Organometallic Chemistry* **2015**, *29* (12), 793-797. https://doi.org/10.1002/aoc.3370.

[7] Taheri-Ledari, R.; Esmaeili, M. S.; Varzi, Z.; Eivazzadeh-Keihan, R.; Maleki, A.; Shalan, A. E. Facile Route to Synthesize Fe_3O_4@acacia–SO_3H Nanocomposite As a Heterogeneous Magnetic System for Catalytic Applications. *RSC Advances* **2020**, *10* (66), 40055-40067. https://doi.org/10.1039/d0ra07986c.

[8] Kumar, A.; Madderla, S.; Dharavath, R.; Nalaparaju, N.; Katta, R.; Gundu, S.; Thumma, V.; Prashanth, B.; Ashok, D. Microwave assisted synthesis of N-substituted acridine-1, 8-dione derivatives: Evaluation of antimicrobial activity. *Journal of Heterocyclic Chemistry* **2022**, *59* (7), 1180-1190.

[9] Tu, S., Miao, C., Gao, Y., Fang, F., Zhuang, Q., Feng, Y., & Shi, D. (2004). A Novel Cascade reaction of aryl aldoxime with dimedone under microwave irradiation: The synthesis of N-hydroxylacridine. *Synlett*, *2004*(02), 0255-0258.

[10] Patil, D., Chandam, D., Mulik, A., Patil, P., Jagadale, S., Kant, R., ... & Deshmukh, M. (2014). Novel Brønsted acidic ionic liquid ([CMIM][CF_3COO]) prompted multicomponent hantzsch reaction for the eco-friendly synthesis of acridinediones: An efficient and recyclable catalyst. *Catalysis letters*, *144*, 949-958.

[11] Gholami Dehbalaei, M.; Foroughifar, N.; Pasdar, H.; Khajeh-Amiri, A. *N*-Propyl Benzoguanamine Sulfonic Acid Supported on Magnetic Fe_3O_4 Nanoparticles: A Novel and Efficient Magnetically Heterogeneous Catalyst for the Synthesis of 1,8-Dioxo-Decahydroacridine Derivatives. *New Journal of Chemistry* **2018**, *42* (1), 327-335. https://doi.org/10.1039/c7nj03508j.

[12] Chate, A. V.; Sukale, S. B.; Ugale, R. S.; Gill, C. H. Baker's Yeast: An Efficient, Green, and Reusable Biocatalyst for the One-Pot Synthesis of Biologically Important *N*-Substituted Decahydroacridine-1,8-Dione Derivatives. *Synthetic Communications* **2017**, *47* (5), 409-420. https://doi.org/10.1080/00397911.2016.1266501.

[13] Zarei, Z.; Akhlaghinia, B. Zn^{II} Doped and Immobilized on Functionalized Magnetic Hydrotalcite (Fe_3O_4/HT-SMTU-Zn^{II}): A Novel, Green and Magnetically Recyclable Bifunctional Nanocatalyst for the One-Pot Multi-Component Synthesis of Acridinediones under Solvent-Free Conditions. *New Journal of Chemistry* **2017**, *41* (24), 15485-15500. https://doi.org/10.1039/c7nj03281a.

[14] Srividya, N., Ramamurthy, P., Shanmugasundaram, P., & Ramakrishnan, V. T. (1996). Synthesis, characterization, and electrochemistry of some acridine-1, 8-dione dyes. *The Journal of Organic Chemistry*, *61*(15), 5083-5089.

[15] Wang, J. L., Liu, B. K., Yin, C., Wu, Q., & Lin, X. F. (2011). Candida antarctica lipase B-catalyzed the unprecedented three-component Hantzsch-type reaction of aldehyde with acetamide and 1, 3-dicarbonyl compounds in non-aqueous solvent. *Tetrahedron*, *67*(14), 2689-2692.
